# Supplementary material for: Measuring coordination between women’s self-help groups and local health systems in rural India: a social network analysis
Source: BMJ Open. 2019 Aug 8;9(8):e028943. doi: 10.1136/bmjopen-2019-028943 (PMC6701569; doi:10.1136/bmjopen-2019-028943)
Supplement: Author's manuscript [file bmjopen-2019-028943.draft_revisions.pdf]

BMJ Open is committed to open peer review. As part of this commitment we make the peer review history of every article we publish publicly available.

When an article is published we post the peer reviewers' comments and the authors' responses online. We also post the versions of the paper that were used during peer review. These are the versions that the peer review comments apply to.

The versions of the paper that follow are the versions that were submitted during the peer review process. They are not the versions of record or the final published versions. They should not be cited or distributed as the published version of this manuscript.

BMJ Open is an open access journal and the full, final, typeset and author-corrected version of record of the manuscript is available on our site with no access controls, subscription charges or pay-per-view fees (http://bmjopen.bmj.com).

If you have any questions on BMJ Open's open peer review process please email info.bmjopen@bmj.com

BMJ Open

Measuring Coordination between Women's Self-Help Groups and Local Health Systems in Rural India: A Social Network Analysis

Journal:	*BMJ Open*
Manuscript ID	bmjopen-2019-028943
Article Type:	Research
Date Submitted by the Author:	09-Jan-2019
Complete List of Authors:	Ruducha, Jenny; Braintree Global Health<br>Hariharan, Divya; Institute for Financial Management and Research<br>Potter, James; Harvard T.H. Chan School of Public Health, Global Health and Population<br>Ahmad, Danish; University of Canberra, Centre for Research and Action in Public Health; Public Health Foundation of India, Indian Institute of Public Health<br>Kumar, Sampath; Rajiv Gandhi Mahila Vikas Pariyojana<br>Mohanan, P.S.; Rajiv Gandhi Mahila Vikas Pariyojana<br>Irani, Laili; Population Council<br>Long, Katelyn ; Boston University School of Public Health
Keywords:	Social Network Analysis, Health Services Coordination Network, Multisectoral Coordination, Demand Side Intervention, Bridging Social Capital, Self Help Groups

SCHOLARONE™
Manuscripts

Measuring Coordination between Women's Self-Help Groups and Local Health Systems in Rural India: A Social Network Analysis

Jenny Ruducha[1], Divya Hariharan[2], James Potter[3], Danish Ahmad[4], Sampath Kumar[5], P.S. Mohanan[5], Laili Irani[6] and Katelyn N.G. Long[7]

1. Braintree Global Health, Lead Partner, Cambridge, MA USA; jenny@braintreeglobalhealth.org
2. Institute for Financial Management and Research, Chennai, India; divya.hariharan@ifmr.ac.in
3. Department of Global Health and Population, Harvard T.H. Chan School of Public Health, Harvard University, Boston, MA; jpotter@g.harvard.edu
4. Centre for Research and Action, Faculty of Health, University of Canberra, Bruce, Canberra, ACT 2601, Canberra; Public Health Foundation of India and Indian Institute of Public Health, Gandhinagar, India; danish.ahmad@canberra.edu.au
5. Rajiv Gandhi Mahila Vikas Pariyojana, Raibareli, Uttar Pradesh, India; sampath97@gmail.com; psmohanan@rgmvp.org
6. Population Council, New Delhi, India; laili.ir@gmail.com
7. School of Public Health, Boston University, Boston, MA; knlong@bu.edu

Correspondence: Dr. Jenny Ruducha, 4559 West 1st Ave, Vancouver, BC, V6R1H7, Canada; jenny@braintreeglobalhealth.org; Tel: 617-480-0158

ABSTRACT WORD COUNT: 300
ARTICLE WORD COUNT: 3,991

KEYWORDS: Social Network Analysis, Health Services Coordination Network, Multisectoral Coordination, Demand Side Intervention, Bridging Social Capital, Self Help Groups

For peer review only - http://bmjopen.bmj.com/site/about/guidelines.xhtml

Measuring Coordination between Women's Self-Help Groups and Local Health Systems in Rural India: A Social Network Analysis

Abstract

Objectives To assess how the health coordination and referral networks between women's Self Help Groups (SHGs) and local health systems have changed over the course of a two-year Learning Phase of the Uttar Pradesh Community Mobilization Project (UPCMP), India.

Design A pre-test, post-test program evaluation using social network survey and qualitative in-depth interviews in the post-test period to analyze changes in network structure and connectivity between key individuals and groups.

Setting The study was conducted in 18 villages located in 3 districts in Uttar Pradesh, India.

Intervention To improve linkages and coordination between SHGs and government health providers by building capacity in leadership, management and community mobilization skills of the SHG federation.

Participants A purposeful sampling that met inclusion criteria. 316 respondents at baseline and 280 respondnets at endline including: SHG members, village and block level government health workers, and other key members of the community (traditional birth attendants, drug sellers, unqualified rural medical providers, pradhans or elected village heads, and religious leaders).

For peer review only - http://bmjopen.bmj.com/site/about/guidelines.xhtml

Main outcome measures Social network analysis measured degree centrality, density, and centralization to assess changes in health services coordination networks at the village and block levels.

Results The health services coordination and referrals networks increased in their density and the number of connections between respondents as measured by average degree centrality have increased along with more diversity of interaction between groups. The network expanded relationships at the village and block levels reflecting the rise of bridging social capital. The ASHA village health worker occupied the central position in the network and her role expanded to sharing information and coordinating services with the SHG members.

Conclusions The creation of new partnerships between traditionally underrepresented communities and local government can serve as vehicle for building social capital that can lead to a more accountable and accessible community health delivery system.

Strengths and limitations of this study

- Original data to study and measure multisectoral coordination intervention between women's SHGs and local health systems
- A detailed examination of health coordination and referral networks in rural India across 18 villages and 3 districts
- Contributes to literature as studies focusing on how economically marginalized women engage through SHGs in coordinating with government departments are not common
- Limitations of social network analysis affects the ability to assess causality and generalizability

For peer review only

For peer review only - http://bmjopen.bmj.com/site/about/guidelines.xhtml

- Limited duration of the linkages intervention during the Learning Phase may reduce ability to detect major changes, as a capacity building interventions take time

Introduction

Background and Study objective

Microfinance institutions comprised of Self-Help Groups (SHGs) are increasingly recognized as promising avenues for expanding health and social services to vulnerable populations. [1][2][3] Activating demand-side interventions through SHGs by providing information on key health practices, strengthening coordination with community health services, and rolling-out schemes and services is an important strategy in reaching vulnerable groups.

Other research demonstrates how increasing poor women's access to working capital can result in improvements in education and health [4][2] as SHGs tend to use their savings and credit for the family's well-being. Enhancing a woman's agency sets into motion new abilities to "exercise bargaining power as well as develop a sense of self-worth, a belief in one's ability to secure desired changes, and the right to control one's life".[5] As women in SHGs build social capital, they also act as an instrument for providing meaningful input into local governance to address deficits in government health systems. [6][7]

Building on the strengths of SHGs, a five-year Uttar Pradesh Community Mobilization Project (UPCMP) India was established in India in 2012 to implement a package of interventions through SHGs aimed at improving the practice of healthy maternal and child

For peer review only - http://bmjopen.bmj.com/site/about/guidelines.xhtml

behaviors and facilitate coordination with the local government health system to improve maternal and newborn health. The project was based on activating an SHG model developed by, Rajiv Gandhi Mahila Vikas Pariyojana (RGMVP), a non-governmental organisation(NGO) to expand SHGs into federated networks of Village Organisations (VOs) and Block Organisations (BOs). Through capacity building and leadership training, women who were poor and from lower castes were encouraged to access financial products, as well as expand their knowledge and ability to advocate for government provided entitlements. The objective of our study was to assess how health services coordination and referral networks between SHGs and local health systems along with other key stakeholders changed over the course of a two-year Learning Phase of the project using social network analysis.

Relevance of coordination networks

Coordination among community institutions is achieved through partnerships to improve responses to public and social issues [8] and are built around norms of reciprocity and trustworthiness. [9] The effectiveness of social networks is dependent on the density of community connections and the vibrancy of associations [10] to expand the relationships between diverse groups and to obtain the full range of knowledge, skills, and resources that the community needs to solve complex problems. Bridging social ties are most effective between people and organisations from typically under-represented community members and people with various kinds of expertise that provide access to schemes and services (such as frontline workers, doctors, etc.). [11]

For peer review only - http://bmjopen.bmj.com/site/about/guidelines.xhtml

SHG's role in coordination networks

SHG platforms have reached 57.9% of villages in India resulting in 4.8 million credit-linked groups in 2010, which demonstrates the broad potential for generating an empowered community voice to demand accountability from government functionaries. [1] Many social service models, such as the UPCMP aim to promote coordination with government health and social services to expand the exchange of resources and generate social capital. [12] [13] [14] [15] [16] When community networks such as SHGs expand by linking with government programs and providers, more resources are available and together, these groups can tackle issues that no one group can resolve by itself. [17]

Participatory policies and community participatory initiatives have been well studied and critiqued. [18][19][20] However, studies focusing on how economically marginalized women engage through SHGs in coordinating with government departments are difficult to find in the published literature. Our study aims to contribute to this research gap by examining the efforts of SHGs in coordinating the delivery of health services with the local government health system, to build and sustain effective networks that enable the flow of resources and services to the poorest communities.

Methods

Setting

Uttar Pradesh (UP), at approximately 200 million population (India Census, 2011) [21] is one of the largest states in India constituting 16.5% of the country's total population. UPCMP

For peer review only - http://bmjopen.bmj.com/site/about/guidelines.xhtml

began in 2012 with a Learning Phase in 100 administrative villages called gram panchayats (GPs) located in ten blocks and eight districts with a goal to scale-up the intervention to 120 blocks over the five year project period. During the Learning Phase of the project, and after reviewing the characteristics of each district, we selected one block from three districts that represented different parts of the state (Hardoi, Mirzapur, and Banda) (**Figure 1**). In each of the selected blocks, we then selected 6 out of the 10 GPs, for a total of 18 GPs in our analysis.

Linkages intervention

The program's linkages strategy aimed to improve coordination between SHGs and government health and social services by expanding the leadership, management and community mobilization skills of the SHG federation at the Village Organisation (VO) and Block Orangisation (BO) levels (Box 1). The UPCMP linkages interventions focused on: 1) facilitation of regular meetings of VO members with AAA workers at village level public health forums as well as SHG/VO meetings; 2) exchange of lists of pregnant and recently delivered women to promote early entry into pre-natal and post-natal care; 3) joint planning of activities at village and block levels; 4) identification and dissemination of information about entitlements and emergency health facilities to all SHG members; and 5) facilitation of regular meetings and feedback between the SHG Block Organisation and with block level government health functionaries.

For peer review only - http://bmjopen.bmj.com/site/about/guidelines.xhtml

Box 1: Structure and Roles of Federated Self Help Groups

As SHGs form in a village, two members from each SHG are voted into a Village Organization (VO), whose members represent on average about 150-250 SHG women and subsequently, two members from each VO across many villages are elected to the BO at the block level representing 5,000-7,000 women.

At the Village level, VO members' main focus was to establish functional relationships between SHG federation members and the three local government health worker cadres designated as "AAAs": the Accredited Social Health Activist (ASHA), a community health worker who gets paid based on her ability to mobilize pregnant and recently delivered women to seek recommended health services and promote institutional deliveries; the Auxillary Nurse Midwife (ANM), a trained midwife who organizes monthly health clinics in each village and supervises the ASHA; and the Anganwadi Worker (AWW), a local nutrition worker who operates a day care center and distributes supplementary food for eligible children and pregnant and lactating women.

At the Block level, SHG members voted into BO leadership roles were tasked with developing relationships with the supervisors of the village level health workers, medical staff working at the primary health care centers and other block level government functionaries and elected office holders.

Study design

We used a pre-test, post-test social network survey and added qualitative in-depth interviews in the post-test period. The study objective was to assess how the health coordination and referral networks between women's Self Help Groups (SHGs) and local health systems have changed over the course of a two-year Learning Phase of the Uttar Pradesh Community Mobilization Project (UPCMP). The survey instruments were based on a validated SNA survey design [22] and questions were adapted to capture aspects of village and block level connections that would be relevant for assessing the program. Qualitative interviews were developed in the endline to complement the SNA in understanding the changing roles and relationships in village health activities that occurred during the implementation period.

For peer review only - http://bmjopen.bmj.com/site/about/guidelines.xhtml

Study sample

The sociometric data was collected through a purposeful sampling methodology that included SHG members and those participating at the federated VO and BO levels, government and private health workers, RGMVP staff and other key stakeholders who were involved in referrals and provision of health advice and services. Block level respondents were only interviewed about their relationship with respondents from two of the six GPs included in the study to reduce the length of the interviews for block level respondents. The total sample was 596, with 316 respondents in the baseline and 280 in the endline for response rates of 94% and 82%, respectively.

Table 1 presents the complete list of respondents along with their acronyms used in SNA visual plot construction, village or block location, and a brief summary of their respective roles. The respondents were grouped into four broad categories corresponding to their affiliation. They included: SHG Structure, RGMVP Staff, Government Health & Nutrition, and "Other" key stakeholders. This approach incorporates elements of both a relational and positional models for examining networks of relations with the network structure. [23]

Table 1: Study Respondents Defined

Acronym (used in SNA plots)	Respondent	Definition
**SHG Structure**		
GM	SHG member	Part of SHG group, involved in saving and borrowing, weekly SHG meetings
G1SS & G2SS	SHG Swasthya Sakhi (two per village)	SHG member who has received special training in health
VOSS	Village Organisation Swasthya Sakhi	SHG leader at village level trained in health and linkages strategy to coordinate health activities
VOM & VOM2	Village Organisation member (two per village)	Leader from 2 village VOs trained in linkages strategy to coordinate health activities

For peer review only - http://bmjopen.bmj.com/site/about/guidelines.xhtml

Acronym (used in SNA plots)	Respondent	Definition
VOB	Village Organisation office bearer	SHG member who holds a post at the federation of SHGs at the village level called Village Organisation
MS	Meeting Sakhi	SHG member who organizes weekly SHG meetings
BOB	Block Organisation office bearer	SHG and Village Organisation member who holds a post at the federation level called Block Organisation
BOR	Block Organisation representative from VO	A member of the Bock Organisation (BO) representing her village organisation
BOPR	Block Organisation Poverty Reduction Committee Member	A dedicated committee in the Block Organisation responsible for development strategies on mobilizing women from poor households into SHGs
**RGMVP Staff**		
RG	RGMVP	Full time field staff who works for the NGO (RGMVP) at block level
RGF	RGMVP Field Officer	Full time field staff who acts as a link between RGMVP and SHGs
RGV	RGMVP Volunteer (Community Volunteer)	A part-time volunteer from the community, who supports the full time field staff in implementation activities
RGT	RGMVP Trainer	RGMVP professional trainer who conducts field trainings for SHGs
ISC	Internal Social Capital	Nominated SHG member who is trained to support health implementation in villages by facilitating discussions in various SHG meetings
**Government Health & Nutrition**		
ASHA	Accredited Social Health Activist	Community health volunteer paid honorarium to promote basic preventative maternal and child services and is supervised by ANM
ANM	Auxiliary Nurse Midwife	Government trained community health worker who conducts monthly outreach clinics providing Maternal and Child care. ANM supervises ASHA and works with AWW
AWW	Anganwadi Worker	Government community worker providing food supplements to young children, adolescent girls, and lactating women along with preschool child education services in government operated village creches
ANMs/LHV	Auxiliary Nurse Midwife Supervisor/ Lady health visitor	ANMs who have been promoted to oversee six sub-centers covering a population of 30,000
ICDSs	Integrated Child Development Scheme Supervisor	Government worker appointed who supervises 25 village level creches called Anganwadi Centers (AWCs) where the AWW works
CDPO	Community Development Program Officer	Government worker in-charge of the ICDS Project at block level who oversees the ICDS supervisor at block level
BDO	Block Development Officer	Government social worker appointed under the ICDS scheme and entrusted with the overall responsibility of the ICDS at block level.
PHM	Primary Health Center Medical Officer	Government Medical Doctor & primary administrator of Primary Health Center (PHC)
PHN	Primary Health Center Nurse	Government Nurse providing nursing services in PHC
PHC	PHC staff	Approximately 13 Government staff appointed to administer primary care services at PHC
PHS	PHC Other	Extra support staff appointed at high delivery load PHCs
CHN	Community Health Center Nurse	Government Nurse appointed at Community Health Centers (CHCs) which constitute the secondary level of health care
CHM	Community Health Center Medical Officer	Government Medical officer who works at CHC to provide clinical services
CHC	CHC Staff	The total strength of staff (approx. 46) appointed by the government to administer specialist and referral care services at CHC
CMO	Chief Medical Officer-in-Charge	Government Medical Doctor who works as the Program director at the District level
CHS	CHC Other	Extra staff appointed at high patient load CHCs
DHN	District Hospital Nurse	Government Nurse working in 300 bed District Hospital for providing comprehensive secondary health care
DHM	District Hospital Medical Officer	Government Medical officer who works at District hospital to provide clinical services
DHOB	District hospital OB/GYN	Government Medical Specialist appointed at District hospital to provide specialist Obstetrics and Gynecology care for women

For peer review only

Acronym (used in SNA plots)	Respondent	Definition
RKS	Rogi Kalyan Samiti	A patient welfare committee set up at government hospital to ensure accountability and protection of patients' rights
**Other Stakeholders**		
PRI	Pradhan or Village Head	Elected head of a village-level constitutional body of local self-government called the panchayat who acts as focal point of contact between government officers and the village community
RL	Religious Leader	Leader of a religion recognized at the community (village) level
RMP	Rural Medical Practitioner (unqualified)	An unqualified medical practitioner who is not formally trained in providing health care services in villages. RMPs are culturally recognized and often first point of health care in communities
Dr	Medical Doctor (qualified)	Formally trained & accredited Medical Doctor that provides health care
PF	Private Health Facility Provider	Accredited medical doctor providing health services in a private health facility
DS	Drug Shop Owner	Owner of a private chemist shop in villages who dispenses drugs and health advice
BPRI	Block Panchayat Raj Institution	The middle tier of the system of local self-government in India operating at administrative level of block that links villages with Districts

* Technical Support Unit (TSU) project workers: TSUC – TSU Community Specialist; TSUN – TSU Nurse Mentor; TSUB – TSU Block Coordinator

Data collection

The baseline data was collected between November 2013 - January 2014 and endline surveys were adminstered between October - November 2015. Surveys were developed in CSPro 6.0 and adminstered using electronic tablets. During both data collection periods, the survey team spent two weeks in each of the three survey districts. Each team had a supervisor that monitored data quality and a UPCMP project representative that provided logistical support.

Analytic structure and network measures

In consideration of the models to guide network measurement and analysis at the individual and whole network level, our work fits into Mays and Scutchfield's (2010) categorization including: degree centrality, density, and centralization. [11] These measures

For peer review only - http://bmjopen.bmj.com/site/about/guidelines.xhtml

are used to assess changes in health services coordination networks within the GP (village) level and between key players comprising GP-block relationships. *Degree centrality* measures the number of connections or ties that each respondent maintains [24] and our analysis is based on a mutual confirmation process. In other words, if one person acknowledges a relationship and the other person does not, that relationship is dropped from the network. *Density is* often used as a measure of social capital [9] and is defined as a ratio of existing relationships or ties in comparison to the potential number of linkages. [25] *Centralization* is an expression of how tightly the network structure is organized around its most central point. The general procedure is to calculate the differences between the centrality scores of the most central point and those of all other points to generate a ratio of the actual sum of differences to the maximum possible sum of differences. [25]

Data analysis

Social network analysis methods have been used to study the structural makeup of cooperation that can lead to stronger collaborative relationships. [26][27] The analysis utilized a combination of two software tools: "R" Version 3 (www.r-project.org) [28] and UCINET Version 6. [29] The plot visualization was developed by using NetDraw. [30]

Results

Descriptive

The characteristics of study respondents for the two study periods are presented in **Table 2**. There were no significant differences between the study respondents for baseline and

For peer review only - http://bmjopen.bmj.com/site/about/guidelines.xhtml

endline in the sociodemographic characteristics except for the affiliation category. There was a decrease of 21 respondents in the SHG group in the endline as reflected in the reduction from 38% of overall respondents to 35%. The median age of the respondents was approximately 40 years. About a quarter to a third of all respondents were not educated. In both rounds of the survey, more than 50% of SHG-level respondents reported having no formal education, while all government health workers reported some education and approximately one third reported post-graduate studies. There was a reduction of scheduled caste and scheduled tribe and an increase in backward caste representation in the endline (see Table 2 footnote). Four district level respondent roles were added in the endline.

Table 2: Comparison of Respondent Background Information

Characteristic	UP CMP Baseline 2013 (Banda, Mirzapur, Hardoi) N (%)	UP CMP Endline 2015 (Banda, Mirzapur, Hardoi) N (%)
**Median Age**	41	40
**Education**		
- No Education	100 (32)	74 (26)
- Some Primary (up to 8th standard)	63 (20)	74 26)
- Some Secondary (up to 11th standard)	40 (13)	40 (14)
- Secondary Completed (12th standard)	2 (1)	3 (1)
- Post-graduate (at least some college completed)	111 (35)	89 (32)
**Caste[1]**		
- Scheduled Caste / Scheduled Tribe	172 (55)	106 (38)
- Other Backward Castes	71 (22)	112 (40)
- General Caste	71 (22)	62 (22)
**Affiliation***		
- SHG Structure	119 (38)	98 (35)
- RGMVP Staff	23 (7)	24 (9)
- Gov Health	77 (24)	77 (28)
- Other	97 (30)	81 (29)

For peer review only - http://bmjopen.bmj.com/site/about/guidelines.xhtml

**Location**		
- Purwa (Hamlet)	34 (11)	33 (12)
- Gram Panchayat (Village)	220 (70)	203 (73)
- Block	62 (20)	34 (12)
- District	0	10 (4)
**TOTAL**	**316**	**280**

The classification of castes is formalized by the Government of India into these categories and we used standard definitions to create these categories. Some of these lower caste designations enable caste groups to receive specific government benefits and subsidies.
* Chi Square test: p=0.05

Respondents were also asked whether they had friends or neighbors who were SHG members, and these results are presented **in Table 3**. The percentage of SHGs in the federated structure that knew other SHG members between the baseline and endline increased significantly from 87% to 100%. The majority of RGMVP staff respondents knew SHG members in their GP, or village. Only about one third of government health workers knew an SHG member, while almost two thirds of respondents from the "Other" category (including the Pradhan, Drug Shop Owner, Traditional Birth Attendant etc.) knew an SHG member in their GP.

Table 3: Respondent with Friends or Neighbors who are SHG Members by "Affiliation" (row percentages)

	UP CMP – 2013 (Banda, Mirzapur, Hardoi) N=316			UP CMP – 2015 (Banda, Mirzapur, Hardoi) N=280		
**Affiliation**	**No (%)**	**Yes (%)**	**DK (%)**	**No (%)**	**Yes (%)**	**DK (%)**
**SHG Structure***	16 (13)	103 (87)	0 (0)	0 (0)	98 (100)	0 (0)
**RGMVP Staff**	4 (17)	18 (78)	1 (4)	1 (4)	23 (96)	0 (0)
**Gov Health**	45 (58)	23 (30)	9 (12)	46 (60)	26 (34)	5 (6)
**Other**	33 (34)	58 (60)	6 (6)	22 (27)	53 (65)	6 (7)
**Total**	95 (31)	202 (64)	16 (5)	69 (25)	200 (71)	11 (4)

* Fischer's Exact Test: p-value <0.0001. All other rows insignificant.

For peer review only - http://bmjopen.bmj.com/site/about/guidelines.xhtml

Social Network Analysis

A fixed set of GP and block level providers and other key community members involved in health care delivery were included in the analysis. Network measures for density, centralization and average degree centrality are presented individually for all 18 villages (6 per district) as well as the average scores across each district for the baseline and endline (**Table 4**). However, since block level respondents were asked about relationships with only two GPs, we confirmed whole GP-block relationships for these two GP-block dyads in each of the three blocks along with results for baseline and endline (**Table 5**).

Network metrics

Density of the overall village health services and referral networks in 18 GPs (villages) were low but increased from baseline to endline in two out of three districts: Banda (7.5% to 10.1%) and Mirzapur (4.7% to 12.6%). Hardoi had a higher density at baseline compared to the other two blocks and had a slight reduction at endline (13.6% to 11.4%). While the average pattens of density changes in blocks during the two year intervention period increased, variability was noted beween individual villages in each of the blocks related to their baseline characteristics, and the degree of change that occurred between baseline and endline. Although Mirzapur's density was the lowest in the GPs measured, they had the greatest growth, as noted by tripling of their scores during the two year UPCMP intervention. Hardoi had a reduction in density in half of GPs but remained within a close range to the other two blocks at endline.

For peer review only - http://bmjopen.bmj.com/site/about/guidelines.xhtml

The GP-block level health services and referral networks take into account the relationships between two villages and block level respondents within each block **(Table 5)**. The baseline densities were lower overall than village level networks but increased after two years of project implementation, although the level of increase was less than in the village level networks. Similar to village level network patterns, Hardoi's GP-block level health services and referral network remained at the highest level in comparison to the other two blocks in both baseline and endline, but decreased slightly in the endline (12.4 to 10.5).

The centralization measures increased two to three-fold during the same two year intervention period, with the highest level of increase in Mirzapur GPs, which doubled on average (15.1% to 29.2%).

"Earlier, one wouldn't get it (information), or we didn't have that much information. Now, on all topics there are no obstacles. If there is stock (medical supplies) at the block, there is no obstacle". - ASHA, Mirzapur

Within the overall average increase in all blocks, GP-level variation was noted. This included a decrease in centralization in two Banda villages and four Hardoi villages. Centralization in the GP-Block networks also increased in all three blocks with the largest gains in Mirzapur (8.9% to 23.9%), while Banda increased from 18.5% to 29.0% and Hardoi from 28.9% to 30.1%.

Mirzapur and Banda had the highest level of increase in average degree centrality but endline

"We organise a meeting where ASHA, ANM and I are present and during that meeting we have discussions on various topics. There have been a lot of changes, children can be taken to the hospital very easily, the ambulance/vehicles arrives and we are able to receive all kinds of services - medicines, vaccination, maintaining weight record." – Banda, BO President

For peer review only - http://bmjopen.bmj.com/site/about/guidelines.xhtml

results suggesting similar levels of provider, SHG and key health provider and community contacts in all three blocks with 3.1 to 3.6 contacts per person. Mirzapur started at the lowest level with only 1.5 confirmed contacts compared to Banda with 2.3 and Hardoi at 4.1 with variability at the individual village level ranging from 0.8 to 6.5 across the three blocks. All GP-block level measures had higher levels of contacts at baseline compared to GP levels with Hardoi reporting a high of 7.7 contacts. Endline average degree centrality measures in GP-block dyads in Banda, Mirzapur and Hardoi were 5.0, 5.2 and 7.2, respectively.

Table 4: Sociometric GP Level Coordination and Referral Networks: Confirmed Density, Centralization and Average Degree Centrality

**District** (1 Block per District)	**Confirmed Density (%)**		**Confirmed Centralization (%)**		**Avg. Degree Centrality**	
	Baseline	Endline	Baseline	Endline	Baseline	Endline
**Banda GPs**						
1	10.0	7.5	17.8	13.3	3.0	2.3
2	5.8	8.3	15.1	33.8	1.8	2.5
3	10.8	16.7	31.1	39.1	3.3	5.0
4	4.2	5.8	16.9	15.1	1.3	1.8
5	7.5	13.3	20.4	28.4	2.3	4.0
6	6.7	9.2	14.2	18.7	2.0	2.8
Average	*7.5*	*10.1*	*19.3*	*24.7*	*2.3*	*3.1*
**Hardoi GPs**						
7	11.7	4.4	23.1	8.6	3.5	1.4
8	19.2	19.1	36.4	32.8	5.8	6.1
9	21.7	15.4	26.7	23.4	6.5	4.9
10	6.7	8.1	14.2	37.9	2.0	2.6
11	11.7	6.6	16.0	19.5	3.5	2.1
12	10.8	14.7	31.1	30.9	3.3	4.7
Average	*13.6*	*11.4*	*24.6*	*25.5*	*4.1*	*3.6*
**Mirzapur GPs**						
13	5.8	11.7	15.1	30.2	1.8	3.5
14	2.5	8.3	11.6	33.8	0.8	2.5

For peer review only - http://bmjopen.bmj.com/site/about/guidelines.xhtml

**District** (1 Block per District)	**Confirmed Density (%)**		**Confirmed Centralization (%)**		**Avg. Degree Centrality**	
15	5.8	12.5	15.1	22.2	1.8	3.8
16	3.3	19.2	10.7	50.7	1.0	5.8
17	8.3	8.3	26.7	19.6	2.5	2.5
18	2.5	15.8	11.6	18.7	0.8	4.8
Average	*4.7*	*12.6*	*15.1*	*29.2*	*1.5*	*3.8*

Table 5: Sociometric GP-Block Level Coordination and Referral Networks: Confirmed Density, Centralization and Average Degree Centrality

**District** (1 Block per District)	**Confirmed Density (%)**		**Confirmed Centralization (%)**		**Avg. Degree Centrality**	
	Baseline	Endline	Baseline	Endline	Baseline	Endline
**Banda**						
GP 2 + Block	7.1	7.4	12.7	34.3	4.4	4.7
GP 5 + Block	5.4	8.3	24.3	23.6	3.4	5.3
Average	6.3	7.9	18.5	29.0	3.9	5.0
**Hardoi**						
GP 8 + Block	11.1	10.9	25.2	34.2	6.9	7.4
GP 9 + Block	13.7	10.1	32.5	26.0	8.5	6.9
Average	12.4	10.5	28.9	30.1	7.7	7.2
**Mirzapur**						
GP 13 + Block	3.4	8.1	9.8	23.8	2.1	5.2
GP 14 + Block	2.0	8.0	7.9	24.0	1.3	5.1
Average	2.7	8.1	8.9	23.9	1.7	5.2

Health services and referral networks

The structures of the health services coordination and referral networks were visualized for all 18 GPs (villages) and for the six GP-Block dyads. We have selected one example from each GP-Block network to illustrate the diversity of changes that occurred in structure and network connectivity. The nodes are labelled by respondent, color-coded to reflect the type of respondent group, and shaped according to the location of the respondent's usual residence or work location **(see Plot Guide**). The nodes are sized by betweeness centrality

For peer review only - http://bmjopen.bmj.com/site/about/guidelines.xhtml

with larger nodes signifying the increasing importance of that respondent in linking others that may not be directly connected to each other.

The structure and the relationships between different individuals and groups changed between baseline and after two years of UP CMP implementation. At baseline, the Mirzapur GP-Block health services and referral network had a “kite-like” structure with only two of the four respondent groups in the network (government health and social workers and the SHG structure) having relationships with no block level connections (**Figure 2a**). At one end, a triad of the three village health and social workers (ASHA, ANM and AWW) were connected together. The AWW had a relationship with an SHG member but had no direct connections with the SHG federated structure. The SHG structures themselves (at the tail end of the kite-like structure) were not integrated together. At endline (**Figure 2b**), the network structure expanded into a series of increasingly connected sets of relationships with the expansion of the number of participants and the inclusion of the traditional birth attendant (TBA) and RGMVP’s Block level Internal Social Capital worker (ISC). The shift from the AWW, a role more focused on nutrition and child development, to the ASHA as the main connector to the SHG structure at two critical points (directly to an SHG member as well as the SHG Village Organisation’s Swasthya Sakhii (VOSS)), expanded the potential for better health services coordination. The SHG structure also became better integrated with the VO level functionaries linked together with the exception of the VOSS.

For peer review only

At baseline, the Banda GP-Block level health services and referral network was organized into two small worlds, with the SHG structure and the RGMVP SHG support structure in one cluster while the government health system and a few other key village respondents were in another separate cluster (**Figure 3a**). Within the SHG small world cluster, RGMVP was at the center of a centralized system resembling a "spoke and wheel" structure with members of the SHG structure at the village and block levels connecting to RGMVP but not to each other.

For peer review only

In the second cluster, the ASHA was a leading connector of village level respondents (AWW, the rural medical practitioner (RMP), an unqualified provider and an SHG member) to the broader government health system at the Block level (ANM supervisor, primary health center medical officer (PHM) and the village oriented ANM provider. By the endline, the two separate clusters formed one network (**Figure 3b**). The ASHA became the main connector between the government health providers and the SHG structure. The SHG Village Organisation members also increased coordination within their own network and RGMVP. RGMVP scaled back from their centralized role at baseline and moved to the periphery of the network while SHG enagagment increased.

> *"We did not have any discussion about these things (Emergency services, C-Section, JSY, BPL card)... If I need(ed) anything then I (would) talk to ANM over the phone. In case of an emergency I tell the woman to go to the hospital... since ASHA has been deputed she takes care of all these things, she gives them information and also goes with them..."- AWW, Banda*

> *"Yes… there are lot of changes, previously we did not know what a meeting is and [now we do and] because of that the connections have become stronger. Sisters from the SHG come, ASHA, AWW and Doctors also come for these meetings and this has helped in spreading awareness. Not just from our block but from our village… women who are more aware and have new information come from far to attend these meetings and enlighten their other sisters by give them more information."* – HW Pradhan Hardoi

In Hardoi, the structure remained similar since they started with one whole network, however an additional project, the Technical Support Unit (TSU), a supply side intervention working with the health system, was just starting during the endline collection period (**Figures 4a – 4b**). We interviewed TSU workers during the endline, colored yellow in the Plot Guide. The ANM held the central position in the endline heath services and referral coordination network and acted as an intermediary between the government health functionaries at the block level and the SHG federated structure. The ASHA also stood out as more direct connectivity was established with different members of the SHGs. Within the SHG platform itself, there were more channels of direct communication between different members and women in designated leadership positions.

For peer review only

Limitations

Our study has limitations related to the constraints of social network analysis, limited prior research on coordination network between SHGs and government and limited duration of testing of the full effects of the lingages intervention. SNA has been mainly used for descriptive purposes and it has been less frequently used for evaluating interventions [31] especially in settings that aim to understand the connections between marginalized groups and the government. Therefore this study cannot be generalized. Sources of bias may include using purposive sampling to identify the respondnet roles in the villages and blocks. Additionally, there are no established criteria for evaluating networks [32] as SNA represents a relatively new multi-disciplinary methodology with limited empirical studies. There is evidence that density and network centrality constructs may influence partnership functioning that affects coordination, but their magnitudes and mechanisms of effect in public health are still largely unknown. [11]

For peer review only

Discussion

After two years of UPCMP project implementation, the health services coordination and referrals networks increased in their density but absolute levels remained low. This was a positive direction as higher density is considered an overall indicator of cohesion and interaction within a network and is often associated with faster rates of information

For peer review only - http://bmjopen.bmj.com/site/about/guidelines.xhtml

diffusion within a community. [9] As information is more quickly shared, processed and appropriated, it can lead to shared decision making. [33]

Our study demonstrated an increase in connections not only between individuals but also an expansion of relationships between groups. Poor and traditionally lower-caste women formed SHGs and developed skills to cross boundaries and develop relationships with health providers. A strategy based on increasing diversity in relationships while actively working to reduce redundancy can lead to improved levels of bridging social capital in a network. Such an approach that connects essential groups can lead to better ways of coordinating and collaborating. [9]

As proxies for bridging social capital, the average degree centrality increased by creating new connections within the two years of UPCMP implementation. The growth in the number of ties from the GP health system to the SHG platform signaled a visible shift to improved communication to coordinate community health services and referrals. However, simply increasing the number of ties may not necessarily result in strong relationships, which is in line with the strength of weak ties theory. [34] Although it is common to surround ourselves with strong ties that include people similar to us in beliefs, values, and access to resources, it is through weak ties that we begin to diversify our networks and create avenues for accessing more varied resources. [9] It is probable that building SHG leadership and coordination skills, which was a major UPCMP intervention focus, facilitated a pathway to faciltate engagement through creation of

For peer review only - http://bmjopen.bmj.com/site/about/guidelines.xhtml

weak ties with other health providers and health system actors. These patterns reflect opportunities for diverse stakeholders to engage in the planning and production of change. [35] The development of a system to monitor coordination at the village level, such as through the Village Health Nutrition and Sanitation Committee (VHNSC) and the SHG federated Village Organisation structure could further direct these efforts to become more systematic and sustainable.

Our analysis showed that the ASHA occupied the central position in the network and her role expanded to coordinating services with the VO Swasthya Sakhi and others in the SHG federation. The ASHA is a community health volunteer and her role evolved from an “activist” and advocate of her village to being part of the government health system and being accountable to the ANM, or nurse midwife as well as the Pradhan in the Village, Health, Nutrition and Sanitation Committee (VHNSC). As an important and recognized member of the community, the ASHA’s modeling of cross-caste and class relationships can influence the governance dynamics in the community as building collaborative networks are considered a more democratic means of developing public policy.[36][37][38][39]

Conclusions

The SHG platform and its federated structure was developed by RGMVP to scale-up and galvanize SHG development to reduce poverty, support women’s empowerment and

For peer review only - http://bmjopen.bmj.com/site/about/guidelines.xhtml

break caste based hierarchies to encourage comprehensive rural development. As such, the SHG platform formed a stronger foundation for the delivery of health systems linkages interventions. The endline Social Network Analysis reveals an expansion of pathways to coordinate health services and referrals for poor, illiterate, and low caste women through the added voices of SHG members. The ability of women with multi-generational social and economic deprivations to broaden their exposure to social networks through SHG membership is a major step forward towards gaining self-confidence in participatory community development.

The increasing success of SHG members and their federatations to forge linkages with the GP health system led to greater coordination for health services delivery while stimulating a more centralized structure of core village health workers, especially the ASHA for increasing accountability. To strengthen their capacity to solve problems that affect the health and well-being of their residents, communities need collaborative processes that include individual empowerment, bridging social ties, and synergy. [40] These ideas feed into the concept of state-society syngery where a mobilized civil society and an active government can work together to build social capital and enhance each other's development efforts. [41][9]

If successful, these partnerships can serve as vehicles for transforming public health practice from a diverse collection of activities and organisations into an organized and accountable delivery system for public health interventions. [11] The challenge will be in

For peer review only

maintaining and continuing to invest in these networks, so that the networks remain dynamic and can adapt to meet new situations and offer new benefits to partners. [22] Future studies that include longitudinal data can provide deeper understanding of the mechanisms by which intersectoral partnerships and community mobilization can lead to effective coordination networks to tackle health problems and their socioeconomic determinants.

For peer review only

Citations/References

[1] Microfinance Summit Campaign. Mapping Pathways out of Poverty: The State of the Microcredit Summit Campaign Report 2015. Online. Microcredit Summit Campaign. 2015.

[2] Leatherman S, Metcalfe M, Geissler K, Dunford C. Integrating microfinance and health strategies: examining the evidence to inform policy and practice. Health policy and planning. 2011 Feb 22;27(2):85-101.
Link: https://academic.oup.com/heapol/article/27/2/85/582611

[3] Ruducha J, Jadhav M. A review of organizational arrangements in microfinance and health programs. Journal of Global Health Reports. 2018 Jul 25;2.
Link: http://www.joghr.org/Article/joghr-02-e2018024

[4] Garikipati S. The impact of lending to women on household vulnerability and women's empowerment: evidence from India. World Development. 2008 Dec 1;36(12):2620-42.
Link: https://www.sciencedirect.com/science/article/abs/pii/S0305750X0800137X

[5] Unifem. Progress of the World's Women 2000. Biennial Report, United Nations Development Fund for Women, New York. 2000.

[6] IFAD, Enabling the Rural Poor to Overcome Poverty, 2003.
Link: https://www.ifad.org/documents/38714170/39309792/e_1.pdf/c75a166d-ecee-46af-abd3-0da951504970

[7] Biscayne P, True Z, Clark C, Harris KP, Anderson CL, Gugerty MK. (2014) Self Help Groups in Development: A Review of the Evidence from South Asia and Sub-Saharan Africa. Evans School of Public Affairs, University of Washington. Prepared for the Strategy, Measurement and Evaluation Team of the Bill and Melinda Gates Foundation.
Link:
https://evans.uw.edu/sites/default/files/EPAR_283_SHG%20Evidence%20Review%20Brief_12.5.14_0.pdf

[8] Nowell B. Profiling capacity for coordination and systems change: The relative contribution of stakeholder relationships in interorganizational collaboratives. American Journal of Community Psychology. 2009 Dec 1;44(3-4):196-212.

For peer review only - http://bmjopen.bmj.com/site/about/guidelines.xhtml

Link: https://link.springer.com/article/10.1007/s10464-009-9276-2

[9] Varda DM. A network perspective on state-society synergy to increase community-level social capital. Nonprofit and voluntary sector quarterly. 2011 Oct;40(5):896-923.
Link: https://journals.sagepub.com/doi/abs/10.1177/0899764010378171

[10] Putnam RD. The prosperous community. The American prospect. 1993 Mar 21;4(13):35-42.
Link: http://staskulesh.com/wp-content/uploads/2012/11/prosperouscommunity.pdf

[11] Mays GP, Scutchfield FD. Improving public health system performance through multiorganizational partnerships. Preventing chronic disease. 2010 Nov;7(6).
Link: https://www.ncbi.nlm.nih.gov/pmc/articles/PMC2995603/

[12] Gulati R, Gargiulo M. Where do interorganizational networks come from?. American journal of sociology. 1999 Mar;104(5):1439-93.
Link: https://www.journals.uchicago.edu/doi/abs/10.1086/210179

[13] Isett KR, Provan KG. The evolution of dyadic interorganizational relationships in a network of publicly funded nonprofit agencies. Journal of Public Administration Research and Theory. 2005 Jan 1;15(1):149-65.
Link: https://academic.oup.com/jpart/article-abstract/15/1/149/971263

[14] Mandell MP, editor. Getting results through collaboration: Networks and network structures for public policy and management. ABC-CLIO; 2001.

[15] Monge, P. R., Fulk, J., Kalman, M. E., Flanagin, A. J., Parnassa, C., & Rumsey, S. (1998). Production of collective action in alliance-based interorganizational communication and information systems. Organization Science, 9, 411-433.
Link: https://pubsonline.informs.org/doi/abs/10.1287/orsc.9.3.411

[16] Westley, F., & Vredenburg, H. (1997). Interorganizational collaboration and the preservation of global biodiversity. Organization Science, 8, 381-403.
Link: https://pubsonline.informs.org/doi/abs/10.1287/orsc.8.4.381

[17] Witte, J. M., Reinicke, W., & Benner, T. (2002, March 24-27). Networked governance: Developing a research agenda. Paper presented at the Annual Convention of the International Studies Association Meetings, New Orleans, LA.

For peer review only - http://bmjopen.bmj.com/site/about/guidelines.xhtml

[18] Jones PS. Urban regeneration's poisoned chalice: is there an impasse in (community) participation-based policy?. Urban Studies. 2003 Mar;40(3):581-601.
Link: https://journals.sagepub.com/doi/abs/10.1080/0042098032000053932

[19] Karim L. Demystifying micro-credit: the Grameen Bank, NGOs, and neoliberalism in Bangladesh. Cultural Dynamics. 2008 Mar;20(1):5-29.
Link: https://journals.sagepub.com/doi/abs/10.1177/0921374007088053

[20] Kothari U. Power, knowledge and social control in participatory development. Participation: The new tyranny. 2001:139-52.

[21] Registrar General I. Census of India 2011: provisional population totals-India data sheet. Office of the Registrar General Census Commissioner, India. Indian Census Bureau. 2011.

[22] Provan KG, Veazie MA, Staten LK, Teufel-Shone NI (2005) The Use of Network Analysis to Strengthen Community Partnerships. Public Administration Review 65: 603–613.
Link: https://onlinelibrary.wiley.com/doi/abs/10.1111/j.1540-6210.2005.00487.x

[23] Burt RS. Brokerage and closure: An introduction to social capital. Oxford university press; 2005.

[24] Freeman LC. Centrality in social networks conceptual clarification. Social networks. 1978 Jan 1;1(3):215-39.
Link:
https://www.bebr.ufl.edu/sites/default/files/Centrality%20in%20Social%20Networks.pdf

[25] Hanneman R, Riddle M. Introduction to social network analysis. Riverside CA: University of California. 2005.

[26] Scott J. Social network analysis. Sage; 2017

[27] Wasserman S, Faust K. Social network analysis: Methods and applications. Cambridge University Press; 1994.

For peer review only - http://bmjopen.bmj.com/site/about/guidelines.xhtml

[28] Team RC. R: A language and environment for statistical computing.
Link: www.r-project.org

[29] Borgatti, S. P., Everett, M. G., & Freeman, L. C. (2002). UCINET for Windows: Software for social network analysis [computer software]. Natick, MA: Analytic Technologies.

[30] Borgatti, S.P., 2002. NetDraw Software for Network Visualization. Analytic Technologies: Lexington, KY.

[31] De Bruin A, McAuliffe E. (2018). Social Network Analysis as a methodological approach to explore health systems: A case study exploring support among senior managers/executives in a hospital network. International Journal of Environmental Research and Public Health. 15;511: 1-11.
Link: https://www.mdpi.com/1660-4601/15/3/511

[32] Balkundi, P.; Kilduff, M. The ties that lead: A social network approach to leadership. Leadersh. Q. 2006, 17, 419–439.
Link: https://www.sciencedirect.com/science/article/pii/S1048984306000038

[33] Wensing M, Van Lieshout J, Koetsenruiter J, Reeves D. Information exchange networks for chronic illness care in primary care practices: an observational study. Implementation Science. 2010 Dec;5(1):3.
Link: https://implementationscience.biomedcentral.com/articles/10.1186/1748-5908-5-3

[34] Granovetter MS. The strength of weak ties. In Social networks 1977 (pp. 347-367).
Link: https://www.sciencedirect.com/science/article/pii/B9780124424500500250

[35] Butterfoss FD, Goodman RM, Wandersman A. Community coalitions for prevention and health promotion: Factors predicting satisfaction, participation, and planning. Health education quarterly. 1996 Feb;23(1):65-79.
Link: https://journals.sagepub.com/doi/abs/10.1177/109019819602300105

[36] Powell WW, Koput KW, Smith-Doerr L. Interorganizational collaboration and the locus of innovation: Networks of learning in biotechnology. Administrative science quarterly. 1996 Mar 1:116-45.
Link: https://www.jstor.org/stable/2393988?seq=1#page_scan_tab_contents

For peer review only - http://bmjopen.bmj.com/site/about/guidelines.xhtml

[37] Jennings Jr ET, Ewalt JA. Interorganizational coordination, administrative consolidation, and policy performance. Public Administration Review. 1998 Sep 1:417-28.
Link: https://www.jstor.org/stable/977551?seq=1#page_scan_tab_contents

[38] Milward HB, Provan KG. Measuring network structure. Public administration. 1998;76(2):387-407.
Link: https://onlinelibrary.wiley.com/doi/abs/10.1111/1467-9299.00106

[39] Provan KG, Milward HB. A preliminary theory of interorganizational network effectiveness: A comparative study of four community mental health systems. Administrative science quarterly. 1995 Mar 1:1-33.
Link: https://www.jstor.org/stable/2393698?seq=1#page_scan_tab_contents

[40] Lasker RD, Weiss ES. Broadening participation in community problem solving: a multidisciplinary model to support collaborative practice and research. Journal of Urban Health. 2003 Mar 1;80(1):14-47.
Link: https://link.springer.com/article/10.1093/jurban/jtg014

[41] Evans P. Government action, social capital and development: reviewing the evidence on synergy. World development. 1996 Jun 1;24(6):1119-32.
Link: https://www.sciencedirect.com/science/article/abs/pii/0305750X96000216

FOOTNOTES

Contributers: Conceptualization and Project Design, JR; Methodology, JR and JP; Network Analysis, JP; Qualitative Analysis: JP, JR, DH; Interpretation: JR, JP, DH; Interviewer Training, JP, DH, JR; Field Investigation, DH, DA, SK, PSM, JP; Draft preparation, JR; Literature Review, JR; Revising and Editing, JR, DH, LI, JP, KNGL, DA.

Acknowledgements: The authors would like to thank Dileep Mavalankar for his leadership of the Uttar Pradesh Community Mobilization Project, Deborah Maine, formerly at Boston University Center for Global Health and Development (BU CGHD) who supported the study's new methods and ME Khan, formerly of the Population Council for his knowledge and open discussions and that led to many ideas about how to study rural health systems. Many staff members of the Rajiv Gandhi Mahila Vikas Pariyojana including Gyanendra Verma and Kapil Patil and the Public Health Foundation

For peer review only - http://bmjopen.bmj.com/site/about/guidelines.xhtml

of India (Anuraag Chaturvedi, Sanjit Sarkar and Srinivasan Soundararajan) assisted in the process of implementing this research project. We also acknowledge the administrative support and data collection oversight provided by Ariel Falconer, formerly at BU CGHD for the baseline and Steven Crimaldi of BU CGHD, for the endline. Lastly, we would like to thank the study respondents who offered their time and information to make this research possible.

Funding: This work was supported by the Bill and Melinda Gates Foundation grant number OPP 1033910.

Data Availability: Additonal unpublished data are available from the corresponding author upon request.

Competing Interests: None declared.

Participant Consent: Consent form (approved by Boston University Ethics Review Board) was read to particiapnts and required a verbal approval.

Ethics Approval: The surveys were approved by the Institutional Review Board of Boston University Medical Campus.

Patient and Public Involvement: No patients or members of the public were involved in the development of research questions, the design of the study, or the development of outcome measures. No patients were asked to advise on interpretation or writing up of results.

Provenance and Peer Review: Not commissioned

For peer review only

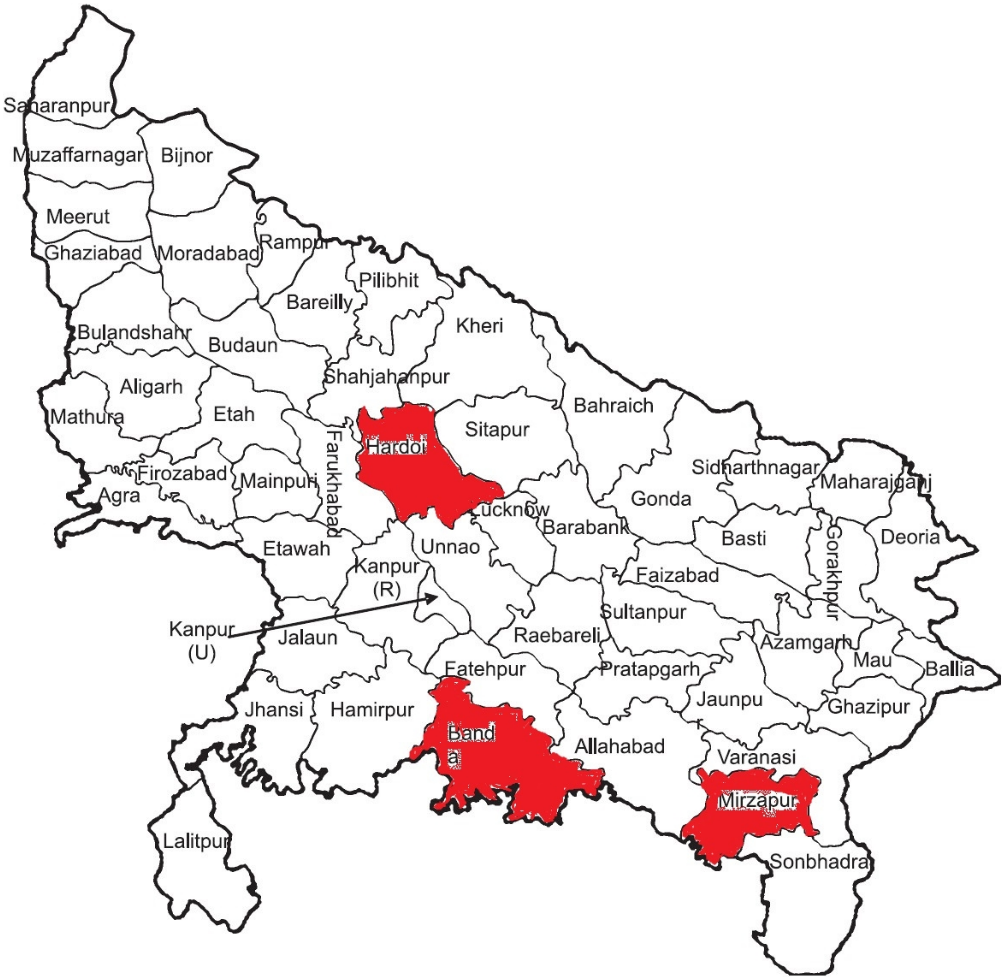

Figure 1: Map of three study districts in Uttar Pradesh, India

296x289mm (100 x 100 DPI)

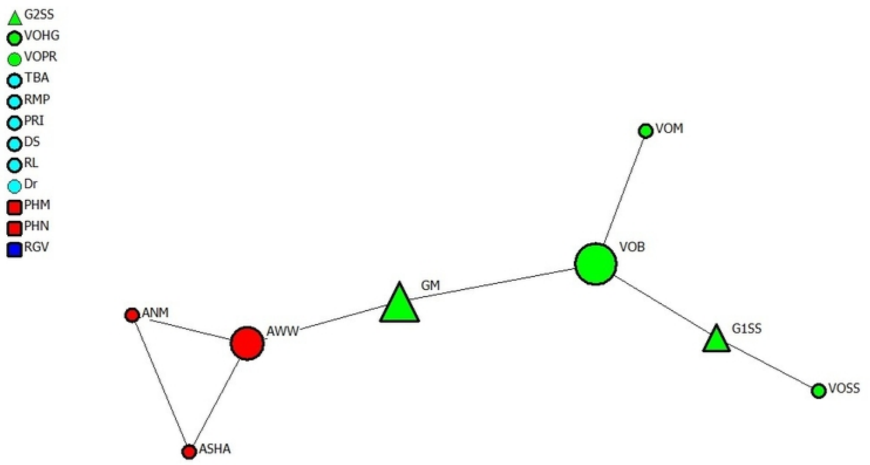

Figure 2a: Mirzapur GP-Block Coordination Network – Baseline

36x22mm (600 x 600 DPI)

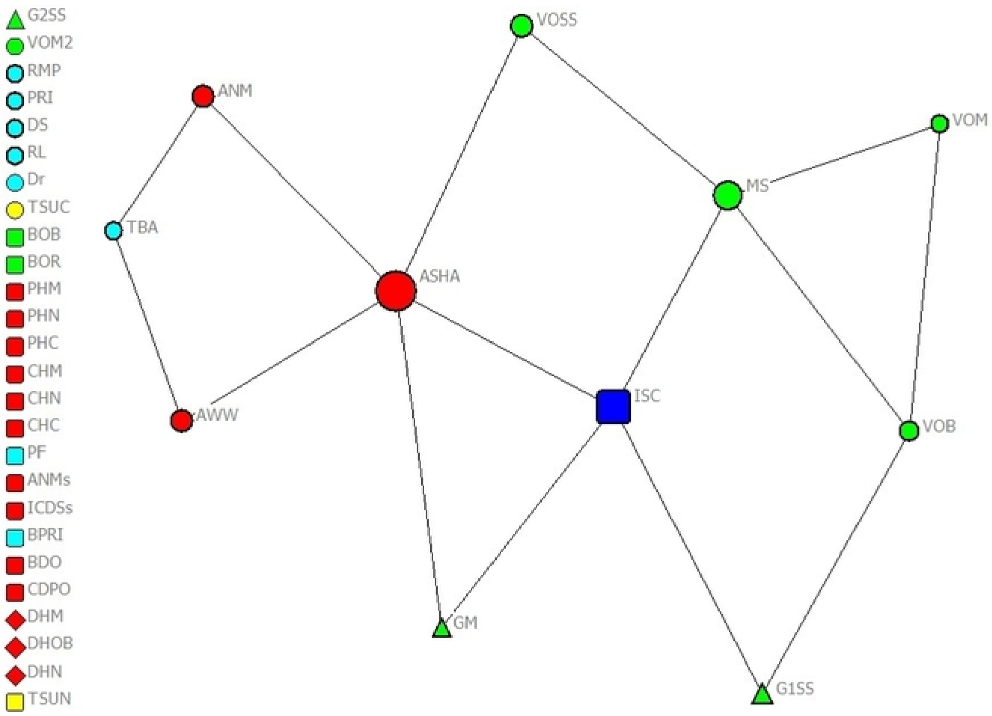

Figure 2b: Mirzapur GP-Block Coordination Network - Endline

28x20mm (600 x 600 DPI)

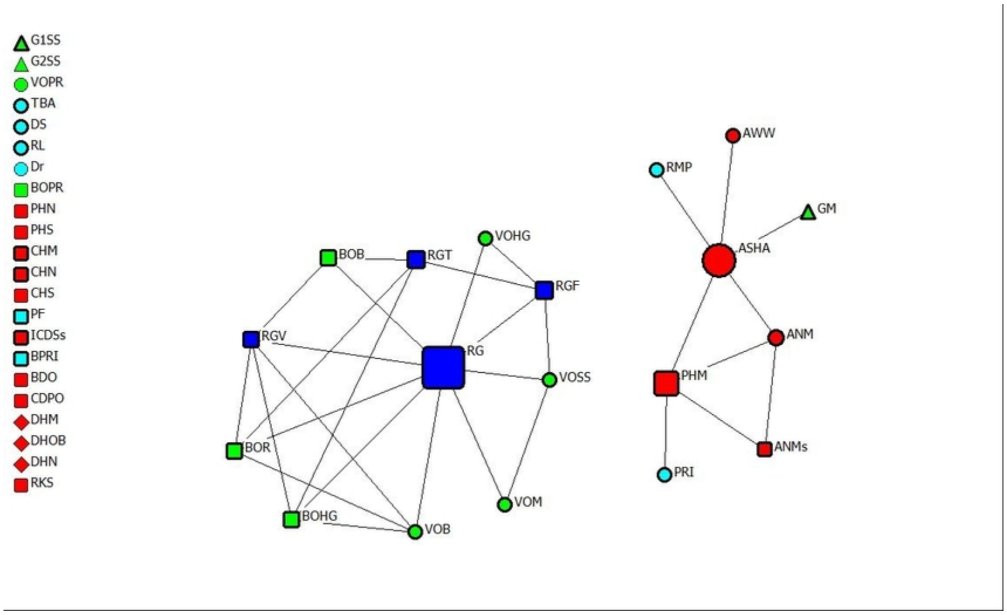

Figure 3a: Banda GP-Block Coordination Network - Baseline

36x21mm (600 x 600 DPI)

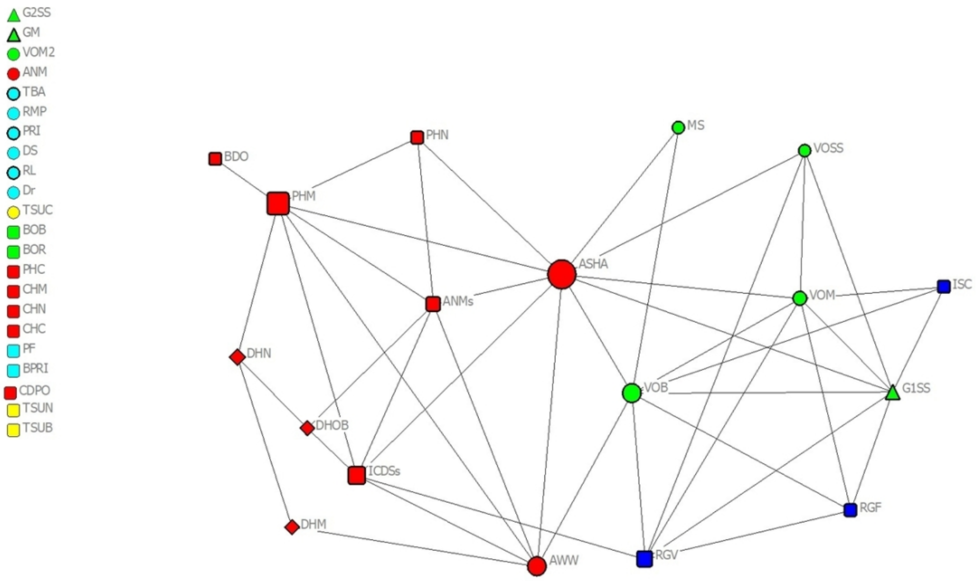

Figure 3b: Banda GP-Block Coordination Network - Endline

52x33mm (600 x 600 DPI)

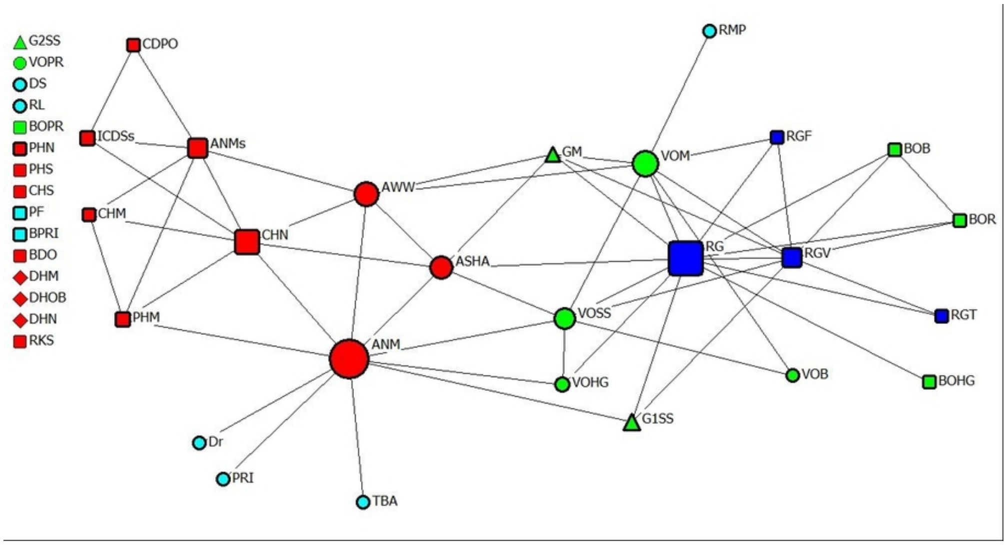

Figure 4a: Hardoi GP-Block Coordination Network - Baseline

38x20mm (600 x 600 DPI)

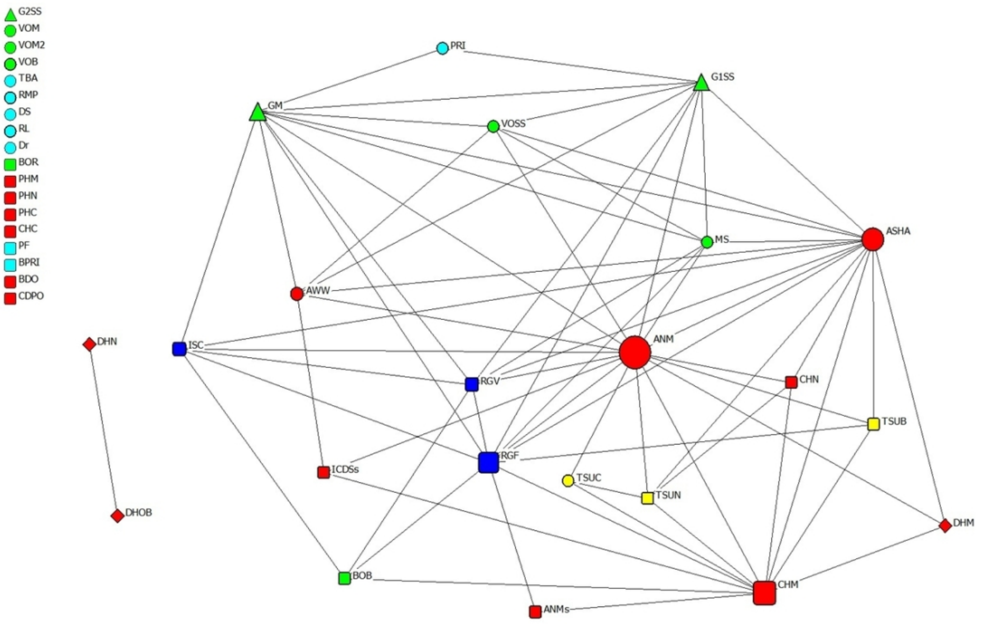

Figure 4b: Hardoi GP-Block Coordination Network - Endline

52x34mm (600 x 600 DPI)

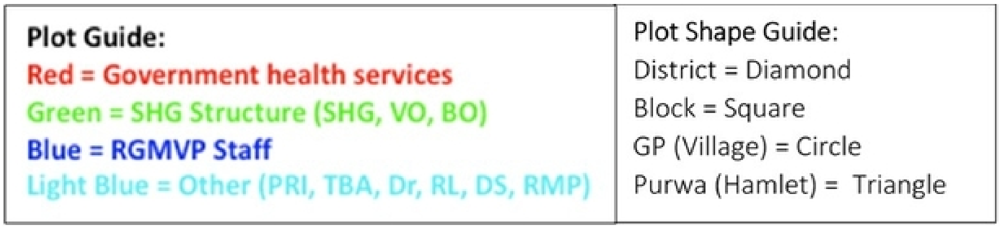

Plot Guide

25x5mm (600 x 600 DPI)

Reporting checklist for cross sectional study.

Based on the STROBE cross sectional guidelines.

Instructions to authors

Complete this checklist by entering the page numbers from your manuscript where readers will find each of the items listed below.

Your article may not currently address all the items on the checklist. Please modify your text to include the missing information. If you are certain that an item does not apply, please write "n/a" and provide a short explanation.

Upload your completed checklist as an extra file when you submit to a journal.

In your methods section, say that you used the STROBE cross sectional reporting guidelines, and cite them as:

von Elm E, Altman DG, Egger M, Pocock SJ, Gotzsche PC, Vandenbroucke JP. The Strengthening the Reporting of Observational Studies in Epidemiology (STROBE) Statement: guidelines for reporting observational studies.

		Reporting Item	Page Number
Title	#1a	Indicate the study's design with a commonly used term in the title or the abstract	1
Abstract	#1b	Provide in the abstract an informative and balanced summary of what was done and what was found	2-3
Background / rationale	#2	Explain the scientific background and rationale for the investigation being reported	4-6
Objectives	#3	State specific objectives, including any prespecified hypotheses	5 & 8
Study design	#4	Present key elements of study design early in the paper	8
Setting	#5	Describe the setting, locations, and relevant dates, including periods of recruitment, exposure, follow-up, and data collection	9 & 11
Eligibility criteria	#6a	Give the eligibility criteria, and the sources and methods of selection of participants.	9-11

For peer review only

	#7	Clearly define all outcomes, exposures, predictors, potential confounders, and effect modifiers. Give diagnostic criteria, if applicable	11-12
Data sources / measurement	#8	For each variable of interest give sources of data and details of methods of assessment (measurement). Describe comparability of assessment methods if there is more than one group. Give information separately for for exposed and unexposed groups if applicable.	12
Bias	#9	Describe any efforts to address potential sources of bias	25
Study size	#10	Explain how the study size was arrived at	11-12
Quantitative variables	#11	Explain how quantitative variables were handled in the analyses. If applicable, describe which groupings were chosen, and why	9-11
Statistical methods	#12a	Describe all statistical methods, including those used to control for confounding	12 & 15
	#12b	Describe any methods used to examine subgroups and interactions	14, 17-18
	#12c	Explain how missing data were addressed	N/A
	#12d	If applicable, describe analytical methods taking account of sampling strategy	N/A
	#12e	Describe any sensitivity analyses	N/A
Participants	#13a	Report numbers of individuals at each stage of study—eg numbers potentially eligible, examined for eligibility, confirmed eligible, included in the study, completing follow-up, and analysed. Give information separately for for exposed and unexposed groups if applicable.	N/A
	#13b	Give reasons for non-participation at each stage	N/A
	#13c	Consider use of a flow diagram	N/A
Descriptive data	#14a	Give characteristics of study participants (eg demographic, clinical, social) and information on exposures and potential confounders. Give information separately for exposed and unexposed groups if applicable.	12-14

For peer review only

	#14b	Indicate number of participants with missing data for each variable of interest	N/A
Outcome data	#15	Report numbers of outcome events or summary measures. Give information separately for exposed and unexposed groups if applicable.	15-17
Main results	#16a	Give unadjusted estimates and, if applicable, confounder-adjusted estimates and their precision (eg, 95% confidence interval). Make clear which confounders were adjusted for and why they were included	N/A
	#16b	Report category boundaries when continuous variables were categorized	N/A
	#16c	If relevant, consider translating estimates of relative risk into absolute risk for a meaningful time period	N/A
Other analyses	#17	Report other analyses done—e.g., analyses of subgroups and interactions, and sensitivity analyses	N/A
Key results	#18	Summarise key results with reference to study objectives	22-26
Limitations	#19	Discuss limitations of the study, taking into account sources of potential bias or imprecision. Discuss both direction and magnitude of any potential bias.	22
Interpretation	#20	Give a cautious overall interpretation considering objectives, limitations, multiplicity of analyses, results from similar studies, and other relevant evidence.	22-26
Generalisability	#21	Discuss the generalisability (external validity) of the study results	3 & 22
Funding	#22	Give the source of funding and the role of the funders for the present study and, if applicable, for the original study on which the present article is based	32

The STROBE checklist is distributed under the terms of the Creative Commons Attribution License CC-BY. This checklist can be completed online using https://www.goodreports.org/, a tool made by the EQUATOR Network in collaboration with Penelope.ai

For peer review only

BMJ Open

Measuring Coordination between Women's Self-Help Groups and Local Health Systems in Rural India: A Social Network Analysis

Journal:	*BMJ Open*
Manuscript ID	bmjopen-2019-028943.R1
Article Type:	Research
Date Submitted by the Author:	16-May-2019
Complete List of Authors:	Ruducha, Jenny; Braintree Global Health<br>Hariharan, Divya; Institute for Financial Management and Research<br>Potter, James; Harvard T.H. Chan School of Public Health, Global Health and Population<br>Ahmad, Danish; University of Canberra, Centre for Research and Action in Public Health; Public Health Foundation of India, Indian Institute of Public Health<br>Kumar, Sampath; Rajiv Gandhi Mahila Vikas Pariyojana<br>Mohanan, P.S.; Rajiv Gandhi Mahila Vikas Pariyojana<br>Irani, Laili; Population Council<br>Long, Katelyn ; Boston University School of Public Health
**Primary Subject Heading**:	Global health
Secondary Subject Heading:	Public health, Sociology, Health services research
Keywords:	Social Network Analysis, Health Services Coordination Network, Multisectoral Coordination, Demand Side Intervention, Bridging Social Capital, Self Help Groups

SCHOLARONE™
Manuscripts

Measuring Coordination between Women's Self-Help Groups and Local Health Systems in Rural India: A Social Network Analysis

Jenny Ruducha[1], Divya Hariharan[2], James Potter[3], Danish Ahmad[4], Sampath Kumar[5], P.S. Mohanan[5], Laili Irani[6] and Katelyn N.G. Long[7]

1. Braintree Global Health, Lead Partner, Cambridge, MA USA; jenny@braintreeglobalhealth.org
2. Institute for Financial Management and Research, Chennai, India; divya.hariharan@ifmr.ac.in
3. Department of Global Health and Population, Harvard T.H. Chan School of Public Health, Harvard University, Boston, MA; jpotter@g.harvard.edu
4. Centre for Research and Action, Faculty of Health, University of Canberra, Bruce, Canberra, ACT 2601, Canberra; Public Health Foundation of India and Indian Institute of Public Health, Gandhinagar, India; danish.ahmad@canberra.edu.au
5. Rajiv Gandhi Mahila Vikas Pariyojana, Raibareli, Uttar Pradesh, India; sampath97@gmail.com; psmohanan@rgmvp.org
6. Population Council, New Delhi, India; laili.ir@gmail.com
7. School of Public Health, Boston University, Boston, MA; knlong@bu.edu

Correspondence: Dr. Jenny Ruducha, 4559 West 1st Ave, Vancouver, BC, V6R1H7, Canada; jenny@braintreeglobalhealth.org; Tel: 617-480-0158

ABSTRACT WORD COUNT: 299
ARTICLE WORD COUNT: 4,043

KEYWORDS: Social Network Analysis, Health Services Coordination Network, Multisectoral Coordination, Demand Side Intervention, Bridging Social Capital, Self Help Groups

For peer review only

Measuring Coordination between Women's Self-Help Groups and Local Health Systems in Rural India: A Social Network Analysis

Abstract

Objectives To assess how the health coordination and emergency referral networks between women's Self Help Groups (SHGs) and local health systems have changed over the course of a two-year Learning Phase of the Uttar Pradesh Community Mobilization Project (UPCMP), India.

Design A pre-test, post-test program evaluation using social network survey to analyze changes in network structure and connectivity between key individuals and groups.

Setting The study was conducted in 18 villages located in 3 districts in Uttar Pradesh, India.

Intervention To improve linkages and coordination between SHGs and government health providers by building capacity in leadership, management and community mobilization skills of the SHG federation.

Participants A purposeful sampling that met inclusion criteria. 316 respondents at baseline and 280 respondnets at endline including: SHG members, village and block level government health workers, and other key members of the community (traditional birth attendants, drug sellers, unqualified rural medical providers, pradhans or elected village heads, and religious leaders).

For peer review only - http://bmjopen.bmj.com/site/about/guidelines.xhtml

Main outcome measures Social network analysis measured degree centrality, density, and centralization to assess changes in health services coordination networks at the village and block levels.

Results The health services coordination and emergency referral networks increased in density and the number of connections between respondents as measured by average degree centrality have increased along with more diversity of interaction between groups. The network expanded relationships at the village and block levels reflecting the rise of bridging social capital. The Accredited Social Health Activist (ASHA), a village health worker, occupied the central position in the network and her role expanded to sharing information and coordinating services with the SHG members.

Conclusions The creation of new partnerships between traditionally underrepresented communities and local government can serve as vehicle for building social capital that can lead to a more accountable and accessible community health delivery system.

Strengths and limitations of this study

- Original data to study and measure multisectoral coordination intervention between women's SHGs and local health systems

- A detailed examination of health coordination and emergency referral networks in rural India across 18 villages and 3 districts

For peer review only

- Contributes to literature as studies focusing on how economically marginalized women engage through SHGs in coordinating with government departments are not common

- Limitations of social network analysis affects the ability to assess causality and generalizability

- Limited duration of the linkages intervention during the Learning Phase may reduce ability to detect major changes, as a capacity building interventions take time

For peer review only

Introduction

Background and Study objective

Microfinance institutions comprised of Self-Help Groups (SHGs) are increasingly recognized as promising avenues for expanding health and social services to vulnerable populations.[1-3] In India, the concept of women's SHGs has evolved over the past three decades. Then and now, the basic SHG structure remains defined as informal groups of 10-20 women from similar socio-economic backgrounds living in close proximity.[4] During the 1980s, the objective was to engage women in collective savings activities and provide access to credit. By the early 1990s, the official SHG and Bank Linkage Programme in India was led by the National Agricultural Bank for Rural Development (NABARD) and focused on loans for livelihood activities. To improve scalability, by early

For peer review only - http://bmjopen.bmj.com/site/about/guidelines.xhtml

2000s, the SHG model grew into a key government programme providing financial access to the poor and addressing issues of social justice to improve the welfare of its members. [5]

The evidence for supporting women's SHGs continues to grow as research demonstrates that increasing poor women's access to working capital can result in improvements in education and health [2,6] and that SHGs tend to use their savings and credit for the family's well-being. Enhancing a woman's agency sets into motion new abilities to "exercise bargaining power as well as develop a sense of self-worth, a belief in one's ability to secure desired changes, and the right to control one's life".[7] As SHGs build social capital, they can also be instrumental in addressing deficits in government health systems.[8-9]

Building on the strengths of SHGs, a five-year Uttar Pradesh Community Mobilization Project (UPCMP) was established in India in 2012 to implement a package of interventions to improve the practice of healthy maternal and child behaviors and coordinate with the local government health system to improve maternal and newborn health. The project was based on activating an SHG model developed by Rajiv Gandhi Mahila Vikas Pariyojana (RGMVP), a non-governmental organisation to expand SHGs into federated networks of Village Organisations and Block Organisations. Through capacity building and leadership training, poor and lower caste women were encouraged to access financial products, and advocate for government health services

For peer review only

and entitlements. The objective of our study was to assess how health services coordination and emergency referral networks between SHGs and local health systems along with other key stakeholders changed over the course of a two-year Learning Phase of the project using social network analysis.

Relevance of coordination networks

Coordination among community institutions is achieved through partnerships to improve responses to public and social issues [10] and are built around norms of reciprocity and trustworthiness.[11] The effectiveness of social networks is dependent on the density of community connections and the vibrancy of associations[12] to expand the relationships between diverse groups and to obtain the full range of knowledge, skills, and resources that the community needs to solve complex problems. Bridging social ties are most effective between people and organisations from typically under-represented communities and groups with expertise that can provide access to schemes and services (such as frontline workers, and doctors).[13]

SHG's role in coordination networks

SHG platforms have reached 57.9% of villages in India resulting in 4.8 million credit-linked groups in 2010, which demonstrates the broad potential for empowering communities to demand accountability from government functionaries.[1] Many social service models, such as the UPCMP, aim to promote coordination with government services to expand the exchange of resources and generate social capital.[14-18] When

For peer review only

community networks such as SHGs create linkages with government programs and providers, more resources are available and together, these groups can tackle issues that no one group can resolve by itself.[19]

Participatory policies and community initiatives have been well studied and critiqued.[20-22] However, studies focusing on how economically marginalized women engage through SHGs in coordinating with government departments are difficult to find in the published literature. Our study aims to contribute to this research gap by examining the efforts of SHGs in coordinating the delivery of health services with the local government health system, to build and sustain effective networks that enable the flow of resources to the poorest communities.

Methods

Setting

Uttar Pradesh (UP), at approximately 200 million population [23] is one of the largest states in India constituting 16.5% of the country's total population. UPCMP began in 2012 with a Learning Phase in 100 administrative villages, called gram panchayats, located in ten blocks within eight districts with a goal to scale-up the intervention to 120 blocks over the five year project period. During the Learning Phase of the project, we selected one block from three districts that represented different parts of the state (Hardoi, Mirzapur, and Banda) (**Figure 1**). In each block, we then selected 6 out of the 10 Gram Panchayats, for a total of 18 Gram Panchayats in our analysis.

For peer review only - http://bmjopen.bmj.com/site/about/guidelines.xhtml

Linkages intervention

The linkages strategy aimed to improve coordination between SHGs and government health and social services by expanding the leadership, management and community mobilization skills of the SHG federation at the Village Organisation and Block Organisation levels (Box 1). The interventions focused on: 1) facilitation of regular meetings of Village Organisation members with local government health workers and between the SHG Block Organisation with block level health functionaries (Box 1); 2) exchange of lists of pregnant and recently delivered women to promote early entry into pre-natal and post-natal care; and 3) identification and dissemination of information about entitlements and emergency health facilities.

Box 1: Structure and Roles of Federated Self Help Groups

At Community/Neighborhood Level, 10-20 women from particularly vulnerable and marginalised households are organised into Self-Help Groups. These SHGs meet regularly for the purpose of addressing common problems through mutual-support. In the case of RGMVP, SHGs are responsible for promoting savings among groups, ensuring credit access from banks, and driving community-based social and behaviour change interventions associated with maternal, newborn and child health. As SHGs form in a village, two members from each SHG are voted into a Village Organisation, whose members represent on average about 150-250 SHG women. Subsequently, two members from each Village Organisation across many villages are elected to the Block Organisation at the block level representing 5,000-7,000 women.

At the Village level, Village Organisation members' main focus was to establish functional relationships between SHG federation members and the three local government health worker cadres designated as "AAAs": the Accredited Social Health Activist (ASHA), a community health worker who gets paid based on her ability to mobilize pregnant and recently delivered women to seek recommended health services and promote institutional deliveries; the Auxillary Nurse Midwife (ANM), a trained midwife who organizes monthly health clinics in each village and supervises the ASHA; and the Anganwadi Worker (AWW), a local nutrition worker who operates a day care center and distributes supplementary food for eligible children and pregnant and lactating women.

At the Block level, Village Organization members are voted into the Block Organisation. Their roles were to develop relationships with the supervisors of the village level health workers, medical staff working at the primary health care centres and other block level government functionaries and elected office holders.

For peer review only - http://bmjopen.bmj.com/site/about/guidelines.xhtml

Study design

We developed a pre-test, post-test social network survey. The study objective was to assess how the health coordination and emergency referral networks between women's SHGs and local health systems changed over the course of the two-year Learning Phase of the Uttar Pradesh Community Mobilization Project (UPCMP). The survey instruments were based on a validated survey design structure [24] and questions were developed and then pre-tested in a social and cultural setting similar to the study population to capture aspects of village and block level connections that would be relevant for assessing the program. One of the main survey questions asked every respondent whether they coordinated health services, including emergency referrals with every other respondent type in the survey (Table1).

Study sample

The data was collected through a purposeful sampling methodology that included SHG members and those participating at the federated Village Organisation and Block Organisation levels, government and private health workers, RGMVP staff and other key stakeholders. Block level respondents were only interviewed about their relationship with respondents from two of the six Gram Panchayats to reduce the length of the interviews for block level respondents.

Certain roles are unique in a village or block, such as the ASHA, ANM, Pradhan or village leader and for those roles the inclusion criteria were that the respondent agree to the

For peer review only - http://bmjopen.bmj.com/site/about/guidelines.xhtml

interview, be over age 18, and be the person responsible for that role at its respective geographic level. For roles that have multiple potential representatives at a given level, survey staff made a list of all potential respondents by consulting with staff at health facilities within each Block, RGMVP program staff, and local stakeholders in each village, and attempted to contact them in a random order. The first potential respondent that was successfully contacted was interviewed. The total sample was 596, with 316 respondents in the baseline and 280 in the endline for response rates of 94% and 82%, respectively.

Table 1 presents the complete list of respondents along with their role designations, the acronyms used in SNA visual plot construction, village or block location, and a brief summary of their respective roles. The respondents were grouped into four broad categories corresponding to their affiliation: SHG Structure, RGMVP Staff, Government Health & Nutrition, and "Other" key stakeholders. This approach incorporates elements of both relational and positional models for examining networks of relations with the network structure.[25]

Table 1: Study Respondents Defined

Acronym (used in SNA plots)	Respondent	Definition
**SHG Structure**		
GM	SHG member	Part of SHG group, involved in saving and borrowing, weekly SHG meetings
G1SS & G2SS	SHG Swasthya Sakhi (two per village)	SHG member who has received special training in health
VOSS	Village Organisation Swasthya Sakhi	SHG leader at village level trained in health and linkages strategy to coordinate health activities

For peer review only - http://bmjopen.bmj.com/site/about/guidelines.xhtml

Acronym (used in SNA plots)	Respondent	Definition
VOM & VOM2	Village Organisation member (two per village)	Leader from 2 village VOs trained in linkages strategy to coordinate health activities
VOB	Village Organisation office bearer	SHG member who holds a post at the federation of SHGs at the village level called Village Organisation
MS	Meeting Sakhi	SHG member who organizes weekly SHG meetings
BOB	Block Organisation office bearer	SHG and Village Organisation member who holds a post at the federation level called Block Organisation
BOR	Block Organisation representative from VO	A member of the Bock Organisation (BO) representing her village organisation
BOPR	Block Organisation Poverty Reduction Committee Member	A dedicated committee in the Block Organisation responsible for development strategies on mobilizing women from poor households into SHGs
**RGMVP Staff**		
RG	RGMVP	Full time field staff who works for the NGO (RGMVP) at block level
RGF	RGMVP Field Officer	Full time field staff who acts as a link between RGMVP and SHGs
RGV	RGMVP Volunteer (Community Volunteer)	A part-time volunteer from the community, who supports the full time field staff in implementation activities
RGT	RGMVP Trainer	RGMVP professional trainer who conducts field trainings for SHGs
ISC	Internal Social Capital	Nominated SHG member who is trained to support health implementation in villages by facilitating discussions in various SHG meetings
**Government Health & Nutrition**		
ASHA	Accredited Social Health Activist	Community health volunteer paid honorarium to promote basic preventative maternal and child services and is supervised by ANM
ANM	Auxiliary Nurse Midwife	Government trained community health worker who conducts monthly outreach clinics providing Maternal and Child care. ANM supervises ASHA and works with AWW
AWW	Anganwadi Worker	Government community worker providing food supplements to young children, adolescent girls, and lactating women along with preschool child education services in government operated village creches
ANMs/LHV	Auxiliary Nurse Midwife Supervisor/ Lady health visitor	ANMs who have been promoted to oversee six sub-centers covering a population of 30,000
ICDSs	Integrated Child Development Scheme Supervisor	Government worker appointed who supervises 25 village level creches called Anganwadi Centers (AWCs) where the AWW works
CDPO	Community Development Program Officer	Government worker in-charge of the ICDS Project at block level who oversees the ICDS supervisor at block level
BDO	Block Development Officer	Government social worker appointed under the ICDS scheme and entrusted with the overall responsibility of the ICDS at block level.
PHM	Primary Health Center Medical Officer	Government Medical Doctor & primary administrator of Primary Health Center (PHC)
PHN	Primary Health Center Nurse	Government Nurse providing nursing services in PHC
PHC	PHC staff	Approximately 13 Government staff appointed to administer primary care services at PHC
PHS	PHC Other	Extra support staff appointed at high delivery load PHCs
CHN	Community Health Center Nurse	Government Nurse appointed at Community Health Centers (CHCs) which constitute the secondary level of health care
CHM	Community Health Center Medical Officer	Government Medical officer who works at CHC to provide clinical services
CHC	CHC Staff	The total strength of staff (approx. 46) appointed by the government to administer specialist and referral care services at CHC
CMO	Chief Medical Officer-in-Charge	Government Medical Doctor who works as the Program director at the District level
CHS	CHC Other	Extra staff appointed at high patient load CHCs
DHN	District Hospital Nurse	Government Nurse working in 300 bed District Hospital for providing comprehensive secondary health care

For peer review only - http://bmjopen.bmj.com/site/about/guidelines.xhtml

Acronym (used in SNA plots)	Respondent	Definition
DHM	District Hospital Medical Officer	Government Medical officer who works at District hospital to provide clinical services
DHOB	District hospital OB/GYN	Government Medical Specialist appointed at District hospital to provide specialist Obstetrics and Gynecology care for women
RKS	Rogi Kalyan Samiti	A patient welfare committee set up at government hospital to ensure accountability and protection of patients' rights
**Other Stakeholders**		
PRI	Pradhan or Village Head	Elected head of a village-level constitutional body of local self-government called the panchayat who acts as focal point of contact between government officers and the village community
RL	Religious Leader	Leader of a religion recognized at the community (village) level
RMP	Rural Medical Practitioner (unqualified)	An unqualified medical practitioner who is not formally trained in providing health care services in villages. RMPs are culturally recognized and often first point of health care in communities
Dr	Medical Doctor (qualified)	Formally trained & accredited Medical Doctor that provides health care
PF	Private Health Facility Provider	Accredited medical doctor providing health services in a private health facility
DS	Drug Shop Owner	Owner of a private chemist shop in villages who dispenses drugs and health advice
BPRI	Block Panchayat Raj Institution	The middle tier of the system of local self-government in India operating at administrative level of block that links villages with Districts

* Technical Support Unit (TSU) project workers: TSUC – TSU Community Specialist; TSUN – TSU Nurse Mentor; TSUB – TSU Block Coordinator

Data collection

The baseline data were collected between November 2013 - January 2014 and endline surveys were adminstered between October - November 2015. Surveys were developed in CSPro 6.0 and adminstered using electronic tablets. During both data collection periods, the survey team spent two weeks in each of the three survey districts. Each team had a supervisor that monitored data quality and a UPCMP project representative that provided logistical support.

Analytic structure and network measures

In consideration of the models to guide network measurement and analysis at the individual and whole network level, our work fits into Mays and Scutchfield's (2010) categorization including: degree centrality, density, and centralization.[13] These

For peer review only

measures are used to assess changes in health services coordination networks within the Gram Panchayat level and between key players comprising Gram Panchayat -Block relationships. *Degree centrality* measures the number of connections or ties that each respondent maintains [26] and our analysis is based on a mutual confirmation process. In other words, if one person acknowledges a relationship and the other person does not, that relationship is dropped from the network. Overall, 31% of ties remained after the confirmation process. The high loss of unconfirmed ties signals a weak level of connectivity whereas ties confirmed by both parties have a higher probability of producing collaborative relationships.[26] In India, the caste system exerts barriers to relationship formation and contributes to a reduction in reciprocity of ties. *Density is* often used as a measure of social capital[11] and is defined as a ratio of existing relationships or ties in comparison to the potential number of linkages.[27] *Centralization* is an expression of how tightly the network structure is organized around its most central point. The general procedure is to calculate the differences between the centrality scores of the most central point and those of all other points to generate a ratio of the actual sum of differences to the maximum possible sum of differences.[27]

Data analysis

Social network analysis methods have been used to study the structural makeup of cooperation that can lead to stronger collaborative relationships.[28-29] The analysis utilized a combination of two software tools: "R" Version 3 (www.r-project.org),[30] and UCINET Version 6.[31] The plot visualization was developed by using NetDraw.[32]

For peer review only - http://bmjopen.bmj.com/site/about/guidelines.xhtml

The study was approved by the Institutional Review Board of Boston University Medical Campus: IRB Number H-32603. No patients or members of the public were involved in the development of research questions, the design of the study, or the development of outcome measures. Also, no patients were asked to advise on interpretation or writing up of results. A consent form (approved by Boston University Ethics Review Board) was read to participants and required a verbal approval.

Results

Descriptive

The characteristics of study respondents for the two study periods are presented in **Table 2**. Most demographic indicators were balanced across baseline and endline rounds using a chi-square test, except for caste (p<0.001). For this indicator, Scheduled Caste and Scheduled Tribe accounted for a greater share of respondents in the baseline compared with the endline, while Other Backward Caste representation was greater in the endline. The median age of the respondents was approximately 40 years. About a quarter to a third of all respondents were not educated. In both rounds of the survey, more than 50% of SHG-level respondents reported having no formal education, while all government health workers reported some education and approximately one third reported post-graduate studies. Four district level respondent roles were added in the endline.

For peer review only - http://bmjopen.bmj.com/site/about/guidelines.xhtml

Table 2: Comparison of Respondent Background Information

Characteristic	UP CMP Baseline 2013 (Banda, Mirzapur, Hardoi) N (%)	UP CMP Endline 2015 (Banda, Mirzapur, Hardoi) N (%)
**Median Age**	41	40
**Education**		
- No Education	100 (32)	74 (26)
- Some Primary (up to 8th standard)	63 (20)	74 26)
- Some Secondary (up to 11th standard)	40 (13)	40 (14)
- Secondary Completed (12th standard)	2 (1)	3 (1)
- Post-graduate (at least some college completed)	111 (35)	89 (32)
**Caste[1*]**		
- Scheduled Caste / Scheduled Tribe	172 (55)	106 (38)
- Other Backward Castes	71 (22)	112 (40)
- General Caste	71 (22)	62 (22)
**Affiliation**		
- SHG Structure	119 (38)	98 (35)
- RGMVP Staff	23 (7)	24 (9)
- Gov Health	77 (24)	77 (28)
- Other	97 (30)	81 (29)
**Location**		
- Purwa (Hamlet)	34 (11)	33 (12)
- Gram Panchayat (Village)	220 (70)	203 (73)
- Block	62 (20)	34 (12)
- District	0	10 (4)
**TOTAL**	**316**	**280**

The classification of castes is formalized by the Government of India into these categories and we used standard definitions to create these categories. Some of these lower caste designations enable caste groups to receive specific government benefits and subsidies. Respondents were asked to self-identify into caste categories in the survey.
* Chi Square test: $p<0.001$

Respondents were also asked whether they had friends or neighbors who were SHG members, and these results are presented **in Table 3**. The percentage of SHGs in the federated structure that knew other SHG members between the baseline and endline increased significantly from 87% to 100%. The majority of RGMVP staff respondents knew SHG members in their Gram Panchayat or village. Only about one third of government health workers knew an SHG member, while almost two thirds of

For peer review only

respondents from the “Other” category (including the Pradhan (local leader), Drug Shop Owner, Traditional Birth Attendant etc.) knew an SHG member.

Table 3: Respondent with Friends or Neighbors who are SHG Members by “Affiliation” (row percentages)

	UP CMP – 2013 (Banda, Mirzapur, Hardoi) N=316			UP CMP – 2015 (Banda, Mirzapur, Hardoi) N=280		
**Affiliation**	**No (%)**	**Yes (%)**	**Don’t Know (%)**	**No (%)**	**Yes (%)**	**Don’t Know (%)**
**SHG Structure***	16 (13)	103 (87)	0 (0)	0 (0)	98 (100)	0 (0)
**RGMVP Staff**	4 (17)	18 (78)	1 (4)	1 (4)	23 (96)	0 (0)
**Gov Health**	45 (58)	23 (30)	9 (12)	46 (60)	26 (34)	5 (6)
**Other**	33 (34)	58 (60)	6 (6)	22 (27)	53 (65)	6 (7)
**Total**	95 (31)	202 (64)	16 (5)	69 (25)	200 (71)	11 (4)

* Fischer’s Exact Test: p-value <0.0001. All other rows insignificant.

Social Network Analysis

A fixed set of Gram Panchayat and block level providers and other key community members involved in health care delivery were included in the analysis. Network measures for density, centralization and average degree centrality are presented individually for all 18 villages (6 per district) as well as the average scores across each district for the baseline and endline (**Table 4**). However, since block level respondents were asked about relationships with only two Gram Panchayats, we confirmed whole Gram Panchayat-block relationships for these two Gram Panchayat-block dyads in each of the three blocks along with results for baseline and endline (**Table 5**).

Network metrics

For peer review only - http://bmjopen.bmj.com/site/about/guidelines.xhtml

Density of the overall village health services and referral networks in 18 Gram Panchayats (villages) were low but increased from baseline to endline in two out of three districts: Banda (7.5% to 10.1%) and Mirzapur (4.7% to 12.6%). Hardoi had a higher density at baseline compared to the other two blocks and had a slight reduction at endline (13.6% to 11.4%). While the average pattens of density changes in blocks during the two year intervention period increased, variability was noted beween individual villages in each of the blocks related to their baseline characteristics, and the degree of change that occurred between baseline and endline. Although Mirzapur's density was the lowest in the Gram Panchayats measured, they had the greatest growth, as noted by tripling of their scores during the two year UPCMP intervention. Hardoi had a reduction in density to half of Gram Panchayats but remained within a close range to the other two blocks at endline.

The Gram Panchayat-block level health services and referral networks take into account the relationships between two villages and block level respondents within each block **(Table 5)**. The baseline densities were lower overall than village level networks but increased after two years of project implementation, although the level of increase was less than in the village level networks. Similar to village level network patterns, Hardoi's Gram Panchayat-block level health services and emergency referral network remained at the highest level in comparison to the other two blocks in both baseline and endline, but decreased slightly in the endline (12.4 to 10.5).

For peer review only - http://bmjopen.bmj.com/site/about/guidelines.xhtml

The centralization measures increased two to three-fold during the same two year intervention period, with the highest level of increase in Mirzapur Gram Panchayats, which doubled on average (15.1% to 29.2%). Within the overall average increase in all blocks, Gram Panchayat-level variation was noted. This included a decrease in centralization in two Banda villages and four Hardoi villages. Centralization in the Gram Panchayat-Block networks also increased in all three blocks with the largest gains in Mirzapur (8.9% to 23.9%), while Banda increased from 18.5% to 29.0% and Hardoi from 28.9% to 30.1%.

Mirzapur and Banda had the highest increase in average degree centrality but endline results suggest similar levels of provider, SHG, key health provider, and community contacts in all three blocks with 3.1 to 3.6 contacts per person. Mirzapur started at the lowest level with only 1.5 confirmed contacts compared to Banda with 2.3 and Hardoi at 4.1 with variability at the individual village level ranging from 0.8 to 6.5 across the three blocks. All Gram Panchyat-block level measures had higher levels of contacts at baseline compared to Gram Panchayat levels with Hardoi reporting a high of 7.7 contacts. Endline average degree centrality measures in Gram Panchayat-block dyads in Banda, Mirzapur and Hardoi were 5.0, 5.2 and 7.2, respectively.

Table 4: Sociometric Gram Panchayat Level Coordination and Referral Networks: Confirmed Density, Centralization and Average Degree Centrality

**District** (1 Block per District)	**Confirmed Density (%)**		**Confirmed Centralization (%)**		**Avg. Degree Centrality**	
	Baseline	Endline	Baseline	Endline	Baseline	Endline

For peer review only - http://bmjopen.bmj.com/site/about/guidelines.xhtml

District (1 Block per District)	Confirmed Density (%)		Confirmed Centralization (%)		Avg. Degree Centrality	
**Banda Gram Panchayats**						
1	10.0	7.5	17.8	13.3	3.0	2.3
2	5.8	8.3	15.1	33.8	1.8	2.5
3	10.8	16.7	31.1	39.1	3.3	5.0
4	4.2	5.8	16.9	15.1	1.3	1.8
5	7.5	13.3	20.4	28.4	2.3	4.0
6	6.7	9.2	14.2	18.7	2.0	2.8
Average	*7.5*	*10.1*	*19.3*	*24.7*	*2.3*	*3.1*
**Hardoi Gram Panchayats**						
7	11.7	4.4	23.1	8.6	3.5	1.4
8	19.2	19.1	36.4	32.8	5.8	6.1
9	21.7	15.4	26.7	23.4	6.5	4.9
10	6.7	8.1	14.2	37.9	2.0	2.6
11	11.7	6.6	16.0	19.5	3.5	2.1
12	10.8	14.7	31.1	30.9	3.3	4.7
Average	*13.6*	*11.4*	*24.6*	*25.5*	*4.1*	*3.6*
**Mirzapur Gram Panchayats**						
13	5.8	11.7	15.1	30.2	1.8	3.5
14	2.5	8.3	11.6	33.8	0.8	2.5
15	5.8	12.5	15.1	22.2	1.8	3.8
16	3.3	19.2	10.7	50.7	1.0	5.8
17	8.3	8.3	26.7	19.6	2.5	2.5
18	2.5	15.8	11.6	18.7	0.8	4.8
Average	*4.7*	*12.6*	*15.1*	*29.2*	*1.5*	*3.8*

Table 5: Sociometric Gram Panchayat (GP)-Block Level Coordination and Referral Networks: Confirmed Density, Centralization and Average Degree Centrality

District (1 Block per District)	Confirmed Density (%)		Confirmed Centralization (%)		Avg. Degree Centrality	
	Baseline	Endline	Baseline	Endline	Baseline	Endline
**Banda**						
GP 2 + Block	7.1	7.4	12.7	34.3	4.4	4.7
GP 5 + Block	5.4	8.3	24.3	23.6	3.4	5.3
Average	6.3	7.9	18.5	29.0	3.9	5.0
**Hardoi**						

For peer review only - http://bmjopen.bmj.com/site/about/guidelines.xhtml

**District** (1 Block per District)	**Confirmed Density (%)**		**Confirmed Centralization (%)**		**Avg. Degree Centrality**	
GP 8 + Block	11.1	10.9	25.2	34.2	6.9	7.4
GP 9 + Block	13.7	10.1	32.5	26.0	8.5	6.9
Average	12.4	10.5	28.9	30.1	7.7	7.2
**Mirzapur**						
GP 13 + Block	3.4	8.1	9.8	23.8	2.1	5.2
GP 14 + Block	2.0	8.0	7.9	24.0	1.3	5.1
Average	2.7	8.1	8.9	23.9	1.7	5.2

For peer review only

Health services and emergency referral networks

The structures of the health services coordination and emergency referral networks were visualized for all 18 Gram Panchayats (villages) and for the six Gram Panchayat-Block dyads. We have selected one example from each Gram Panchayat-Block network to illustrate the diversity of changes that occurred in structure and network connectivity. The nodes are labelled by respondent, color-coded to reflect the type of respondent group, and shaped according to the location of the respondent's usual residence or work location. The nodes are sized by betweeness centrality with larger nodes signifying the increasing importance of that respondent in linking others that may not be directly connected to each other.

The structure and the relationships between different individuals and groups changed between baseline and after two years of UP CMP implementation. At baseline, the Mirzapur Gram Panchayat-Block health services and referral network had a "kite-like" structure with only two of the four respondent groups in the network (government health and social workers and the SHG structure) having relationships with no block level connections (**Figure 2a**). At one end, a triad of the three village health and social

For peer review only - http://bmjopen.bmj.com/site/about/guidelines.xhtml

workers (ASHA, ANM and AWW) were connected together. The AWW had a relationship with an SHG member but had no direct connections with the SHG federated structure. The SHG structures themselves (at the tail end of the kite-like structure) were not integrated. At endline (**Figure 2b**), the network structure expanded into a series of connected sets of relationships with the expansion of the number of participants and the inclusion of the traditional birth attendant (TBA) and RGMVP's Block level Internal Social Capital worker (ISC). The shift from the AWW, a role more focused on nutrition and child development, to the ASHA as the main connector to the SHG structure at two critical points, directly to an SHG member as well as the SHG Village Organisation's Swasthya Sakhii (VOSS), expanded the potential for better health services coordination. The SHG structure also became better integrated with the Village Organisation level functionaries linked together with the exception of the Village Organisation Swasthya Sakhi.

At baseline, the Banda Gram Panchayat-Block level health services and referral network was organized into two small worlds, with the SHG structure and the RGMVP SHG support structure in one cluster while the government health system and a few other key village respondents were in another separate cluster (**Figure 3a**). Within the SHG small world cluster, RGMVP was at the center of a centralized system resembling a "spoke and wheel" with members of the SHG structure at the village and block levels connecting to RGMVP but not to each other.

For peer review only

In the second cluster, the ASHA was a leading connector of village level respondents (AWW, the rural medical practitioner (RMP), an unqualified provider and an SHG member) to the broader government health system at the Block level (ANM supervisor, primary health center medical officer (PHM) and the village oriented ANM provider). By the endline, the two separate clusters formed one network (**Figure 3b**). The ASHA became the main connector between the government health providers and the SHG structure. The SHG Village Organisation members also increased coordination within their own network and RGMVP. RGMVP scaled back from their centralized role at baseline and moved to the periphery of the network while SHG enagagment increased.

In Hardoi, the structure remained similar since they started with one whole network, however an additional project, the Technical Support Unit (TSU), a supply side intervention working with the health system, was just starting during the endline collection period (**Figures 4a – 4b**). The ANM held the central position in the endline heath services and emergency referral coordination network and acted as an intermediary between the government health functionaries at the block level and the SHG federated structure. The ASHA also stood out as more direct connectivity was established with different members of the SHGs. Within the SHG platform itself, there were more channels of direct communication between different members and women in designated leadership positions.

For peer review only - http://bmjopen.bmj.com/site/about/guidelines.xhtml

Limitations

Our study has limitations related to the constraints of social network analysis, minimal prior research on coordination network between SHGs and government and limited duration of testing of the full effects of the lingages intervention. SNA has been mainly used for descriptive purposes and it has been less frequently used for evaluating interventions [33] especially in settings that aim to understand the connections between marginalized groups and the government. Therefore this study cannot be generalized. Sources of bias may include using purposive sampling to identify the respondnet roles in the villages and blocks. Additionally, there are no established criteria for evaluating networks [34] as SNA represents a relatively new multi-disciplinary methodology with limited empirical studies. There is evidence that density and network centrality constructs may influence partnership functioning that affects coordination, but their magnitudes and mechanisms of effect in public health are still largely unknown.[13]

Discussion

After two years of UPCMP project implementation, the health services coordination and emergency referral networks increased in their density but absolute levels remained low. This was a positive direction as higher density is considered an overall indicator of

For peer review only

cohesion and interaction within a network and is often associated with faster rates of information diffusion within a community.[11] As information is more quickly shared, processed and appropriated, it can lead to shared decision making.[35]

Our study demonstrated an increase in connections not only between individuals but also an expansion of relationships between groups. Poor and traditionally lower-caste SHGs developed skills to cross boundaries and forge relationships with health providers. Traditionally bound by societal and cultural expectations, it has been difficult to overcome social and structural barriers, which limit interactions among heterogenous groups. A strategy based on increasing diversity in relationships while actively working to reduce redundancy can lead to improved levels of bridging social capital in a network. Such an approach that connects essential groups can lead to better ways of coordinating and collaborating.[11] Therefore, SHGs with increasing skills and confidence in engaging with health services providers are creating in-roads to better health services for themselves and their communities.

As proxies for bridging social capital, the average degree centrality increased by creating new connections within the two years of UPCMP implementation. However, simply increasing the number of ties may not necessarily result in strong relationships, as noted by the weak ties theory.[36] Although it is common to surround ourselves with strong ties that include people similar to us in beliefs, values, and access to resources, it is through weak ties that we begin to diversify our networks and create avenues for

For peer review only - http://bmjopen.bmj.com/site/about/guidelines.xhtml

accessing more varied resources.[11] It is probable that building SHG leadership and coordination skills, which was a major UPCMP intervention, facilitated a pathway for SHGs to create weak ties with the health system. These patterns reflect opportunities for diverse stakeholders to engage in the planning and production of change.[37]

Our analysis showed that the ASHA occupied the central position in the network and her role expanded to coordinating services with the Village Organization Swasthya Sakhi and others in the SHG federation. The ASHA is a community health volunteer and her role evolved from an "activist" and advocate of her village to being part of the government health system and being accountable to the ANM, or nurse midwife as well as the Pradhan (leader) in the Village, Health, Nutrition and Sanitation Committee. As an important and recognized member of the community, the ASHA's modeling of cross-caste and class relationships can influence the governance dynamics as building collaborative networks are considered a more democratic means of developing public policy.[38-41]

Conclusions

The SHG platform and its federated structure was developed by RGMVP to scale-up and galvanize SHG development to reduce poverty, support women's empowerment and break caste based hierarchies to encourage comprehensive rural development. The endline social network analysis reveals an expansion of pathways to coordinate health services and emergency referrals for poor, illiterate, and low caste women through the

For peer review only - http://bmjopen.bmj.com/site/about/guidelines.xhtml

added voices of SHG members. The ability of women with multi-generational social and economic deprivations to broaden their exposure to social networks through SHG membership is a major step forward towards gaining self-confidence in participatory community development.

The increasing success of SHG federatations to forge linkages with the health system led to greater coordination for health services delivery while stimulating a more centralized structure of core village health workers. Collaborative processes that include individual empowerment, bridging social ties, and synergy can strengthen the capacity to solve problems.[42] These ideas feed into the concept of state-society syngery where a mobilized civil society and an active government can work together to build social capital and enhance each other's development efforts.[11,43]

If successful, these partnerships can serve as vehicles for transforming public health from a diverse collection of activities and organisations into an organized and accountable delivery system.[13] The challenge will be in maintaining these networks, so that they remain dynamic and offer new benefits to partners.[24] Future studies that include longitudinal data can provide deeper understanding of the mechanisms by which intersectoral partnerships and community mobilization lead to effective coordination networks to tackle health problems and their socioeconomic determinants.

For peer review only - http://bmjopen.bmj.com/site/about/guidelines.xhtml

Citations/References

[1] Microfinance Summit Campaign. Mapping Pathways out of Poverty: The State of the Microcredit Summit Campaign Repor, 2015.
Link: https://stateofthecampaign.org/read-the-full-2015-report/

[2] Leatherman S, Metcalfe M, Geissler K, Dunford C. Integrating microfinance and health strategies: examining the evidence to inform policy and practice. *Health Policy Plan* Feb 22 2011;27(2):85-101.
Link: https://doi.org/10.1093/heapol/czr014

[3] Ruducha J, Jadhav M. A review of organizational arrangements in microfinance and health programs. *J Glob Health Rep* 2018 Jul;25;2.
Link: http://www.joghr.org/Article/joghr-02-e2018024

[4] Messiana DN. Social Networking of Self-Help Groups in Tribal Villages of Srikakulam District of Andhra Pradesh – A critical Analysis (Doctoral Disseration), *Acharya NG Ranga Agricultural Unviersity,* 2012
Link: http://krishikosh.egranth.ac.in/displaybitstream?handle=1/67004

[5] Reddy CS, Manak S. Self-help groups: A keystone of microfinance in India-women empowerment and social security. *Andhra Pradesh Mahila Abhivruddhi Society (APMAS) India: Hyderabad* Oct 2005.
Link: http://www.arabic.microfinancegateway.org/sites/default/files/mfg-en-paper-self-help-groups-a-keystone-of-microfinance-in-india-women-empowerment-social-security-oct-2005.pdf

[6] Garikipati S. The impact of lending to women on household vulnerability and women's empowerment: evidence from India. *World Development* Dec 1 2008;36(12):2620-42.
Link: https://doi.org/10.1016/j.worlddev.2007.11.008

[7] Unifem. Progress of the World's Women 2000. Biennial Report, United Nations Development Fund for Women, New York, 2000.
Link: http://www.unwomen.org/en/digital-library/publications/2000/1/progress-of-the-world-s-women-2000

[8] IFAD. Enabling the Rural Poor to Overcome Poverty, 2003.

For peer review only - http://bmjopen.bmj.com/site/about/guidelines.xhtml

Link: https://www.ifad.org/documents/38714170/39309792/e_1.pdf/c75a166d-ecee-46af-abd3-0da951504970

[9] Anderson L. Self-Help Groups in Development: A Review of Evidence from South Asia and Sub-Saharan Africa. Evans School of Public Affairs, University of Washington. Prepared for the Strategy, Measurement and Evaluation Team of the Bill and Melinda Gates Foundation, 2014.
Link:
https://evans.uw.edu/sites/default/files/EPAR_283_SHG%20Evidence%20Review%20Brief_12.5.14_0.pdf

[10] Nowell B. Profiling capacity for coordination and systems change: The relative contribution of stakeholder relationships in interorganizational collaboratives. *Am J Community Psychol* Dec 1 2009;44(3-4):196-212.
Link: https://doi.org/10.1007/s10464-009-9276-2

[11] Varda DM. A network perspective on state-society synergy to increase community-level social capital. *Nonprofit Volunt Sect Q* Oct 2011;40(5):896-923.
Link: https://doi.org/10.1177/0899764010378171

[12] Putnam RD. The prosperous community. *Am Prospect* Mar 21 1993;4(13):35-42.
Link: http://staskulesh.com/wp-content/uploads/2012/11/prosperouscommunity.pdf

[13] Mays GP, Scutchfield FD. Improving public health system performance through multiorganizational partnerships. *Prev Chronic Dis* Nov 2010;7(6).
Link: https://www.ncbi.nlm.nih.gov/pmc/articles/PMC2995603/

[14] Gulati R, Gargiulo M. Where do interorganizational networks come from?. *Am J Psychol Mar* 1999;104(5):1439-93.
Link: https://doi.org/10.1086/210179

[15] Isett KR, Provan KG. The evolution of dyadic interorganizational relationships in a network of publicly funded nonprofit agencies. *J Public Adm Res Theory* Jan 1 2005;15(1):149-65.
Link: https://doi.org/10.1093/jopart/mui008

[16] Mandell MP, editor. Getting results through collaboration: Networks and network structures for public policy and management ABC-CLIO, 2001.

For peer review only - http://bmjopen.bmj.com/site/about/guidelines.xhtml

[17] Monge PR, Fulk J, Kalman, ME., Flanagin AJ, Parnassa C, Rumsey S. Production of collective action in alliance-based interorganizational communication and information systems. *Organization Science* 1998;9:411-433.
Link: https://doi.org/10.1287/orsc.9.3.411

[18] Westley F, Vredenburg H. Interorganizational collaboration and the preservation of global biodiversity. *Organization Science* 1997;8:381-403.
Link: https://doi.org/10.1287/orsc.8.4.381

[19] Witte JM, Reinicke W, Benner T. Networked governance: Developing a research agenda. Paper presented at the *Annual Convention of the International Studies Association Meetings*, New Orleans, LA, 2002.

[20] Jones PS. Urban regeneration's poisoned chalice: is there an impasse in (community) participation-based policy? *Urban Stud* 2003;40(3):581-601.
Link: https://doi.org/10.1080/0042098032000053932

[21] Karim L. Demystifying micro-credit: the Grameen Bank, NGOs, and neoliberalism in Bangladesh. *Cultural Dynamics* Mar 2008;20(1):5-29.
Link: https://doi.org/10.1177/0921374007088053

[22] Kothari U, Cooke B (editor). Power, knowledge and social control in participatory development. In *Participation: The New Tyrann*y? Zed Books, London2001;139-52.

[23] Registrar General I. Census of India 2011: provisional population totals-India data sheet. Office of the Registrar General Census Commissioner, India. *Indian Census Bureau* 2011.

[24] Provan KG, Veazie MA, Staten LK, Teufel-Shone NI. The Use of Network Analysis to Strengthen Community Partnerships. *Public Adm Rev* 2005;65:603–613.
Link: https://doi.org/10.1111/j.1540-6210.2005.00487.x

[25] Burt RS. Brokerage and closure: An introduction to social capital. Oxford University Press, 2005.

[26] Freeman LC. Centrality in social networks conceptual clarification. *Soc Networks* 1978;1(3):215-39.

For peer review only - http://bmjopen.bmj.com/site/about/guidelines.xhtml

Link: https://www.bebr.ufl.edu/sites/default/files/Centrality%20in%20Social%20Networks.pdf

[27] Hanneman R, Riddle M. Introduction to Social Network Analysis. *Riverside CA: University of California,* Oct 1 2005.
Link: http://faculty.ucr.edu/~hanneman/nettext/

[28] Scott J. Social Network Analysis. Sage, 2017

[29] Wasserman S, Faust K. Social network analysis: Methods and applications. Cambridge University Press, 1994.

[30] Team RC. R: A language and environment for statistical computing.
Link: www.r-project.org

[31] Borgatti SP, Everett MG, Freeman LC. UCINET for Windows: Software for social network analysis [computer software]. Natick, MA: Analytic Technologies, 2002.

[32] Borgatti SP. NetDraw Software for Network Visualization. Analytic Technologies: Lexington, KY, 2002.

[33] DeBruin A, McAuliffe E. Social Network Analysis as a methodological approach to explore health systems: A case study exploring support among senior managers/executives in a hospital network. *Int J Environ Res Public Health* 2018;15(3):511.
Link: https://doi.org/10.3390/ijerph15030511

[34] Balkundi P, Kilduff M. The ties that lead: A social network approach to leadership. *Leadersh Q* 2006;17:419–439.
Link: https://doi.org/10.1016/j.leaqua.2006.01.001

[35] Wensing M, Van Lieshout J, Koetsenruiter J, Reeves D. Information exchange networks for chronic illness care in primary care practices: an observational study. *Implement Sci* 2010;5(1):3.
Link: https://doi.org/10.1186/1748-5908-5-3

For peer review only - http://bmjopen.bmj.com/site/about/guidelines.xhtml

[36] Granovetter MS. The strength of weak ties. *In Social Networks* Jan 1 1977 (pp. 347-367) Academic Press.
Link: https://doi.org/10.1016/B978-0-12-442450-0.50025-0

[37] Butterfoss FD, Goodman RM, Wandersman A. Community coalitions for prevention and health promotion: Factors predicting satisfaction, participation, and planning. *Health Educ Q* 1996;23(1):65-79.
Link: https://doi.org/10.1177/109019819602300105

[38] Powell WW, Koput KW, Smith-Doerr L. Interorganizational collaboration and the locus of innovation: Networks of learning in biotechnology. *Adm Sci Q* 1996;116-45.
Link: https://www.jstor.org/stable/2393988

[39] Jennings Jr ET, Ewalt JA. Interorganizational coordination, administrative consolidation, and policy performance. *Public Adm Rev* 1998;417-28.
Link: https://www.jstor.org/stable/977551

[40] Milward HB, Provan KG. Measuring network structure. *Public Adm* 1998;76(2):387-407.
Link: https://doi.org/10.1111/1467-9299.00106

[41] Provan KG, Milward HB. A preliminary theory of interorganizational network effectiveness: A comparative study of four community mental health systems. *Adm Sci Q* 1995;1-33.
Link: https://www.jstor.org/stable/2393698

[42] Lasker RD, Weiss ES. Broadening participation in community problem solving: a multidisciplinary model to support collaborative practice and research. *J Urban Health* 2003;80(1):14-47.
Link: https://doi.org/10.1093/jurban/jtg014

[43] Evans P. Government action, social capital and development: reviewing the evidence on synergy. *World Development* 1996;24(6):1119-32.
Link: https://doi.org/10.1016/0305-750X(96)00021-6

For peer review only - http://bmjopen.bmj.com/site/about/guidelines.xhtml

FOOTNOTES

Contributers: Conceptualization and Project Design, JR; Methodology, JR and JP; Network Analysis, JP; Qualitative Analysis: JP, JR, DH; Interpretation: JR, JP, DH; Interviewer Training, JP, DH, JR; Field Investigation, DH, DA, SK, PSM, JP; Draft preparation, JR; Literature Review, JR; Revising and Editing, JR, DH, LI, JP, KNGL, DA.

Acknowledgements: The authors would like to thank Dileep Mavalankar for his leadership of the Uttar Pradesh Community Mobilization Project, Deborah Maine, formerly at Boston University Center for Global Health and Development (BU CGHD) who supported the study's new methods and ME Khan, formerly of the Population Council for his knowledge and open discussions and that led to many ideas about how to study rural health systems. Many staff members of the Rajiv Gandhi Mahila Vikas Pariyojana including Gyanendra Verma and Kapil Patil and the Public Health Foundation of India (Anuraag Chaturvedi, Sanjit Sarkar and Srinivasan Soundararajan) assisted in the process of implementing this research project. We also acknowledge the administrative support and data collection oversight provided by Ariel Falconer, formerly at BU CGHD for the baseline and Steven Crimaldi of BU CGHD, for the endline. Lastly, we would like to thank the study respondents who offered their time and information to make this research possible.

Funding: This work was supported by the Bill and Melinda Gates Foundation grant number OPP 1033910.

Data Availability: Additonal unpublished data are available from the corresponding author upon request.

Competing Interests: None declared.

Participant Consent: Consent form (approved by Boston University Ethics Review Board) was read to particiapnts and required a verbal approval.

Ethics Approval: The surveys were approved by the Institutional Review Board of Boston University Medical Campus**:** IRB Number H-32603.

Patient and Public Involvement: No patients or members of the public were involved in the development of research questions, the design of the study, or the development of outcome measures. No patients were asked to advise on interpretation or writing up of results.

Provenance and Peer Review: Not commissioned

For peer review only

FIGURE CAPTIONS:

Figure 1: Map of Three Study Districts in Uttar Pradesh, India

Figure 2a: Mirzapur GP-Block Coordination Network - Baseline

Figure 2b: Mirzapur GP-Block Coordination Network - Endline

Figure 3a: Banda GP-Block Coordination Network - Baseline

Figure 3b: Banda GP-Block Coordination Network - Endline

Figure 4a: Hardoi GP-Block Coordination Network - Baseline

Figure 4b: Hardoi GP-Block Coordination Network - Endline

For peer review only

For peer review only - http://bmjopen.bmj.com/site/about/guidelines.xhtml

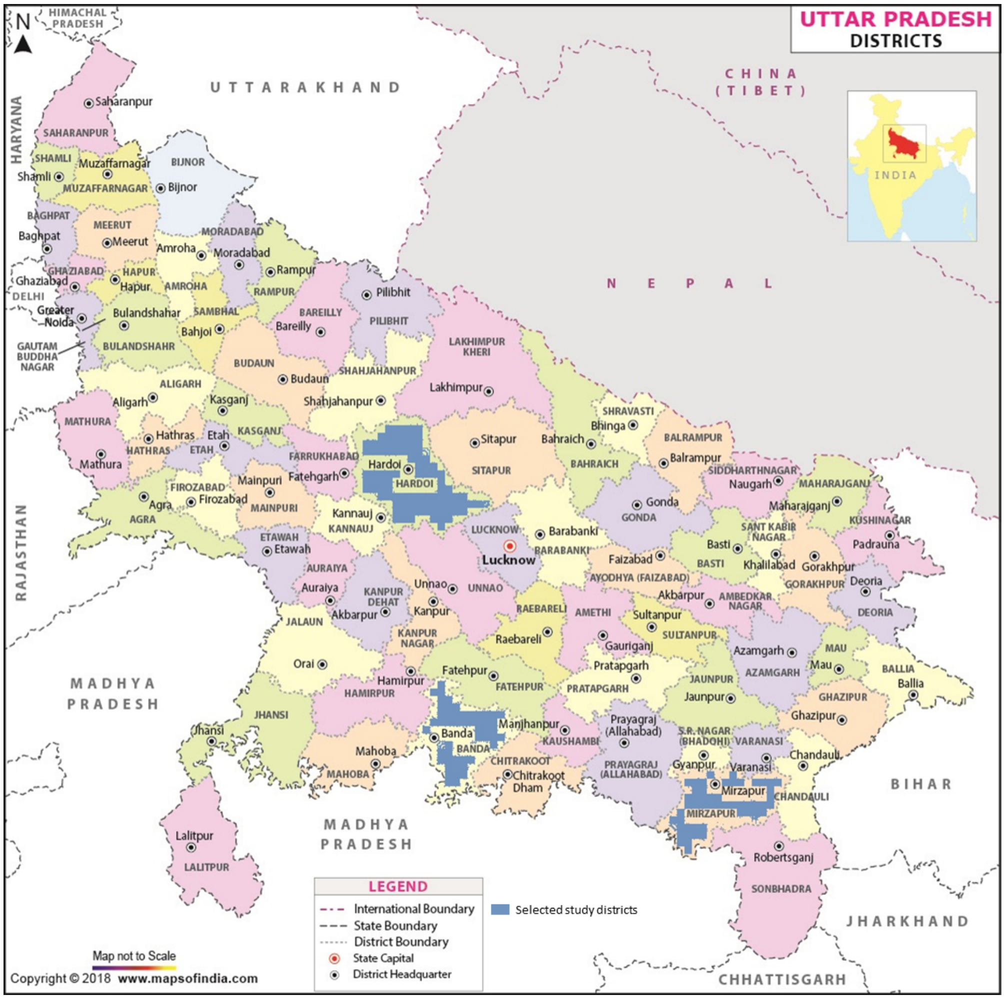

Figure 1: Map of Three Study Districts in Uttar Pradesh, India

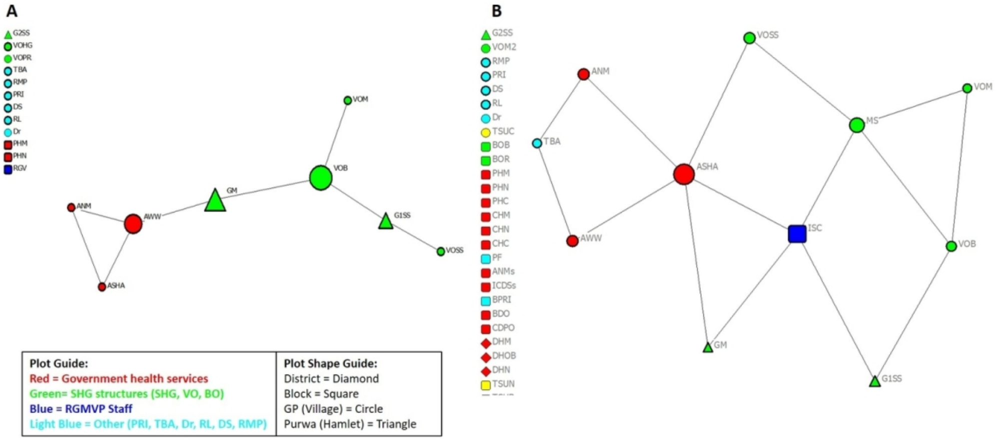

Figure 2a: Mirzapur GP-Block Coordination Network - Baseline
Figure 2b: Mirzapur GP-Block Coordination Network - Endline

62x28mm (600 x 600 DPI)

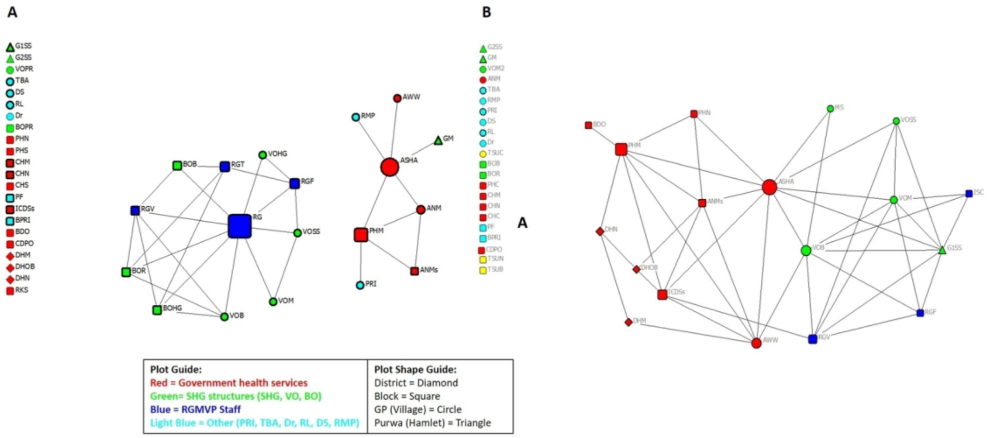

Figure 3a: Banda GP-Block Coordination Network - Baseline
Figure 3b: Banda GP-Block Coordination Network - Endline

71x32mm (600 x 600 DPI)

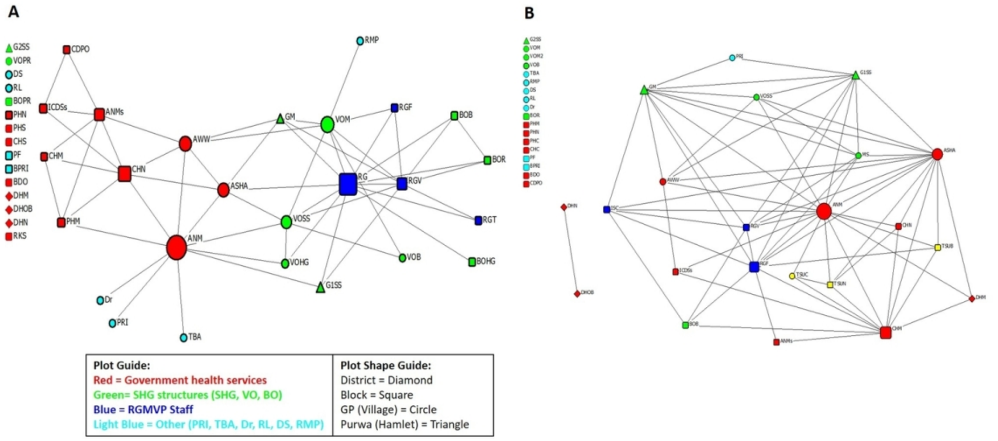

Figure 4a: Hardoi GP-Block Coordination Network - Baseline
Figure 4b: Hardoi GP-Block Coordination Network - Endline

64x29mm (600 x 600 DPI)

Reporting checklist for cross sectional study.

Based on the STROBE cross sectional guidelines.

Instructions to authors

Complete this checklist by entering the page numbers from your manuscript where readers will find each of the items listed below.

Your article may not currently address all the items on the checklist. Please modify your text to include the missing information. If you are certain that an item does not apply, please write "n/a" and provide a short explanation.

Upload your completed checklist as an extra file when you submit to a journal.

In your methods section, say that you used the STROBE cross sectional reporting guidelines, and cite them as:

von Elm E, Altman DG, Egger M, Pocock SJ, Gotzsche PC, Vandenbroucke JP. The Strengthening the Reporting of Observational Studies in Epidemiology (STROBE) Statement: guidelines for reporting observational studies.

		Reporting Item	Page Number
Title	#1a	Indicate the study's design with a commonly used term in the title or the abstract	1
Abstract	#1b	Provide in the abstract an informative and balanced summary of what was done and what was found	2-3
Background / rationale	#2	Explain the scientific background and rationale for the investigation being reported	4-6
Objectives	#3	State specific objectives, including any prespecified hypotheses	5 & 8
Study design	#4	Present key elements of study design early in the paper	8
Setting	#5	Describe the setting, locations, and relevant dates, including periods of recruitment, exposure, follow-up, and data collection	9 & 11
Eligibility criteria	#6a	Give the eligibility criteria, and the sources and methods of selection of participants.	9-11

For peer review only

	#7	Clearly define all outcomes, exposures, predictors, potential confounders, and effect modifiers. Give diagnostic criteria, if applicable	11-12
Data sources / measurement	#8	For each variable of interest give sources of data and details of methods of assessment (measurement). Describe comparability of assessment methods if there is more than one group. Give information separately for for exposed and unexposed groups if applicable.	12
Bias	#9	Describe any efforts to address potential sources of bias	25
Study size	#10	Explain how the study size was arrived at	11-12
Quantitative variables	#11	Explain how quantitative variables were handled in the analyses. If applicable, describe which groupings were chosen, and why	9-11
Statistical methods	#12a	Describe all statistical methods, including those used to control for confounding	12 & 15
	#12b	Describe any methods used to examine subgroups and interactions	14, 17-18
	#12c	Explain how missing data were addressed	N/A
	#12d	If applicable, describe analytical methods taking account of sampling strategy	N/A
	#12e	Describe any sensitivity analyses	N/A
Participants	#13a	Report numbers of individuals at each stage of study—eg numbers potentially eligible, examined for eligibility, confirmed eligible, included in the study, completing follow-up, and analysed. Give information separately for for exposed and unexposed groups if applicable.	N/A
	#13b	Give reasons for non-participation at each stage	N/A
	#13c	Consider use of a flow diagram	N/A
Descriptive data	#14a	Give characteristics of study participants (eg demographic, clinical, social) and information on exposures and potential confounders. Give information separately for exposed and unexposed groups if applicable.	12-14

For peer review only

	#14b	Indicate number of participants with missing data for each variable of interest	N/A
Outcome data	#15	Report numbers of outcome events or summary measures. Give information separately for exposed and unexposed groups if applicable.	15-17
Main results	#16a	Give unadjusted estimates and, if applicable, confounder-adjusted estimates and their precision (eg, 95% confidence interval). Make clear which confounders were adjusted for and why they were included	N/A
	#16b	Report category boundaries when continuous variables were categorized	N/A
	#16c	If relevant, consider translating estimates of relative risk into absolute risk for a meaningful time period	N/A
Other analyses	#17	Report other analyses done—e.g., analyses of subgroups and interactions, and sensitivity analyses	N/A
Key results	#18	Summarise key results with reference to study objectives	22-26
Limitations	#19	Discuss limitations of the study, taking into account sources of potential bias or imprecision. Discuss both direction and magnitude of any potential bias.	22
Interpretation	#20	Give a cautious overall interpretation considering objectives, limitations, multiplicity of analyses, results from similar studies, and other relevant evidence.	22-26
Generalisability	#21	Discuss the generalisability (external validity) of the study results	3 & 22
Funding	#22	Give the source of funding and the role of the funders for the present study and, if applicable, for the original study on which the present article is based	32

The STROBE checklist is distributed under the terms of the Creative Commons Attribution License CC-BY. This checklist can be completed online using https://www.goodreports.org/, a tool made by the EQUATOR Network in collaboration with Penelope.ai

For peer review only

Measuring Coordination between Women's Self-Help Groups and Local Health Systems in Rural India: A Social Network Analysis

Journal:	*BMJ Open*
Manuscript ID	bmjopen-2019-028943.R2
Article Type:	Research
Date Submitted by the Author:	12-Jun-2019
Complete List of Authors:	Ruducha, Jenny; Braintree Global Health<br>Hariharan, Divya; Institute for Financial Management and Research<br>Potter, James; Harvard T.H. Chan School of Public Health, Global Health and Population<br>Ahmad, Danish; University of Canberra, Centre for Research and Action in Public Health; Public Health Foundation of India, Indian Institute of Public Health<br>Kumar, Sampath; Rajiv Gandhi Mahila Vikas Pariyojana<br>Mohanan, P.S.; Rajiv Gandhi Mahila Vikas Pariyojana<br>Irani, Laili; Population Council<br>Long, Katelyn ; Boston University School of Public Health
**Primary Subject Heading**:	Global health
Secondary Subject Heading:	Public health, Sociology, Health services research
Keywords:	Social Network Analysis, Health Services Coordination Network, Multisectoral Coordination, Demand Side Intervention, Bridging Social Capital, Self Help Groups

SCHOLARONE™
Manuscripts

Measuring Coordination between Women's Self-Help Groups and Local Health Systems in Rural India: A Social Network Analysis

Jenny Ruducha[1], Divya Hariharan[2], James Potter[3], Danish Ahmad[4], Sampath Kumar[5], P.S. Mohanan[5], Laili Irani[6] and Katelyn N.G. Long[7]

1. Braintree Global Health, Lead Partner, Cambridge, MA USA; jenny@braintreeglobalhealth.org
2. Institute for Financial Management and Research, Chennai, India; divya.hariharan@ifmr.ac.in
3. Department of Global Health and Population, Harvard T.H. Chan School of Public Health, Harvard University, Boston, MA; jpotter@g.harvard.edu
4. Centre for Research and Action, Faculty of Health, University of Canberra, Bruce, Canberra, ACT 2601, Canberra; Public Health Foundation of India and Indian Institute of Public Health, Gandhinagar, India; danish.ahmad@canberra.edu.au
5. Rajiv Gandhi Mahila Vikas Pariyojana, Raibareli, Uttar Pradesh, India; sampath97@gmail.com; psmohanan@rgmvp.org
6. Population Council, New Delhi, India; laili.ir@gmail.com
7. School of Public Health, Boston University, Boston, MA; knlong@bu.edu

Correspondence: Dr. Jenny Ruducha, 4559 West 1st Ave, Vancouver, BC, V6R1H7, Canada; jenny@braintreeglobalhealth.org; Tel: 617-480-0158

ABSTRACT WORD COUNT: 299
ARTICLE WORD COUNT: 4,058

KEYWORDS: Social Network Analysis, Health Services Coordination Network, Multisectoral Coordination, Demand Side Intervention, Bridging Social Capital, Self Help Groups

For peer review only

Measuring Coordination between Women's Self-Help Groups and Local Health Systems in Rural India: A Social Network Analysis

Abstract

Objectives To assess how the health coordination and emergency referral networks between women's Self Help Groups (SHGs) and local health systems have changed over the course of a two-year Learning Phase of the Uttar Pradesh Community Mobilization Project (UPCMP), India.

Design A pre-test, post-test program evaluation using social network survey to analyze changes in network structure and connectivity between key individuals and groups.

Setting The study was conducted in 18 villages located in 3 districts in Uttar Pradesh, India.

Intervention To improve linkages and coordination between SHGs and government health providers by building capacity in leadership, management and community mobilization skills of the SHG federation.

Participants A purposeful sampling that met inclusion criteria. 316 respondents at baseline and 280 respondents at endline including: SHG members, village and block level government health workers, and other key members of the community (traditional birth attendants, drug sellers, unqualified rural medical providers, pradhans or elected village heads, and religious leaders).

For peer review only - http://bmjopen.bmj.com/site/about/guidelines.xhtml

Main outcome measures Social network analysis measured degree centrality, density, and centralization to assess changes in health services coordination networks at the village and block levels.

Results The health services coordination and emergency referral networks increased in density and the number of connections between respondents as measured by average degree centrality have increased, along with more diversity of interaction between groups. The network expanded relationships at the village and block levels reflecting the rise of bridging social capital. The Accredited Social Health Activist (ASHA), a village health worker, occupied the central position in the network and her role expanded to sharing information and coordinating services with the SHG members.

Conclusions The creation of new partnerships between traditionally underrepresented communities and local government can serve as vehicle for building social capital that can lead to a more accountable and accessible community health delivery system.

Strengths and limitations of this study

- Original data to study and measure multisectoral coordination intervention between women's SHGs and local health systems

- A detailed examination of health coordination and emergency referral networks in rural India across 18 villages and 3 districts

For peer review only

- Contributes to literature as studies focusing on how economically marginalized women engage through SHGs in coordinating with government departments are not common

- Limitations of social network analysis affects the ability to assess causality and generalizability

- Limited duration of the linkages intervention during the Learning Phase may reduce ability to detect major changes, as a capacity building interventions take time

Introduction

Background and Study objective

Microfinance institutions comprised of Self-Help Groups (SHGs) are increasingly recognized as promising avenues for expanding health and social services to vulnerable populations.[1-3] In India, the concept of women's SHGs has evolved over the past three decades. The basic SHG structure remains defined as informal groups of 10-20 women from similar socio-economic backgrounds living in close proximity.[4] During the 1980s, the objective was to engage women in collective savings activities and provide access to credit. By the early 1990s, the official SHG and Bank Linkage Programme in India was led by the National Agricultural Bank for Rural Development (NABARD) and focused on loans for livelihood activities. To improve scalability, by early 2000s, the SHG model

For peer review only

grew into a key government programme providing financial access to the poor and addressing issues of social justice to improve the welfare of its members. [5]

The evidence for supporting women's SHGs continues to grow as research demonstrates that increasing poor women's access to working capital can result in improvements in education and health [2,6] and that SHGs tend to use their savings and credit for the family's well-being. Enhancing a woman's agency sets into motion new abilities to "exercise bargaining power as well as develop a sense of self-worth, a belief in one's ability to secure desired changes, and the right to control one's life".[7] As SHGs build social capital, they can also be instrumental in addressing deficits in government health systems.[8-9]

Building on the strengths of SHGs, a five-year Uttar Pradesh Community Mobilization Project (UPCMP) was established in India in 2012 to implement a package of interventions to improve the practice of healthy maternal and child behaviors and coordinate with the local government health system to improve maternal and newborn health. The project was based on activating an SHG model developed by Rajiv Gandhi Mahila Vikas Pariyojana (RGMVP), a non-governmental organisation, to expand SHGs into federated networks of Village Organisations and Block Organisations. Through capacity building and leadership training, poor and lower caste women were encouraged to access financial products, and advocate for government health services and entitlements. The objective of our study was to assess how health services

For peer review only

coordination and emergency referral networks between SHGs and local health systems along with other key stakeholders changed over the course of a two-year Learning Phase of the project using social network analysis.

Relevance of coordination networks

Coordination among community institutions is achieved through partnerships that improve responses to public and social issues [10] and are built around norms of reciprocity and trustworthiness.[11] The effectiveness of social networks is dependent on the density of community connections and the vibrancy of associations[12] to expand the relationships between diverse groups and to obtain the full range of knowledge, skills, and resources that the community needs to solve complex problems. Bridging social ties are most effective between people and organisations from typically under-represented communities and groups with expertise that can provide access to schemes and services (such as frontline workers, and doctors).[13]

SHG's role in coordination networks

SHG platforms have reached 57.9% of villages in India resulting in 4.8 million credit-linked groups in 2010, which demonstrates the broad potential for empowering communities to demand accountability from government functionaries.[1] Many social service models, such as the UPCMP, aim to promote coordination with government services to expand the exchange of resources and generate social capital.[14-18] When community networks such as SHGs create linkages with government programs and

For peer review only - http://bmjopen.bmj.com/site/about/guidelines.xhtml

providers, more resources are available and together, these groups can tackle issues that no one group can resolve by itself.[19]

Participatory policies and community initiatives have been well studied and critiqued.[20-22] However, studies focusing on how economically marginalized women engage through SHGs in coordinating with government departments are difficult to find in the published literature. Our study aims to contribute to this research gap by examining the efforts of SHGs in coordinating the delivery of health services with the local government health system, in order to build and sustain effective networks that enable the flow of resources to the poorest communities.

Methods

Setting

Uttar Pradesh (UP), at approximately 200 million population, [23] is one of the largest states in India constituting 16.5% of the country's total population. Uttar Pradesh Community Mobilization Project (UPCMP) began in 2012 with a Learning Phase in 100 administrative villages, called gram panchayats, located in ten blocks within eight districts with a goal to scale-up the intervention to 120 blocks over the five year project period. During the Learning Phase of the project, we selected one block from three districts that represented different parts of the state (Hardoi, Mirzapur, and Banda) (**Figure 1**). In each block, we then selected 6 out of the 10 Gram Panchayats, for a total of 18 Gram Panchayats in our analysis.

For peer review only - http://bmjopen.bmj.com/site/about/guidelines.xhtml

Linkages intervention

The linkages strategy aimed to improve coordination between SHGs and government health and social services by expanding the leadership, management and community mobilization skills of the SHG federation at the Village Organisation and Block Organisation levels (Box 1). The interventions focused on: 1) facilitation of regular meetings of Village Organisation members with local government health workers and between the SHG Block Organisation with block level health functionaries (Box 1); 2) exchange of lists of pregnant and recently delivered women to promote early entry into pre-natal and post-natal care; and 3) identification and dissemination of information about entitlements and emergency health facilities.

Box 1: Structure and Roles of Federated Self Help Groups

At Community/Neighborhood Level, 10-20 women from particularly vulnerable and marginalised households are organised into Self-Help Groups. These SHGs meet regularly for the purpose of addressing common problems through mutual-support. In the case of RGMVP, SHGs are responsible for promoting savings among groups, ensuring credit access from banks, and driving community-based social and behaviour change interventions associated with maternal, newborn and child health. As SHGs form in a village, two members from each SHG are voted into a Village Organisation, whose members represent on average about 150-250 SHG women. Subsequently, two members from each Village Organisation across many villages are elected to the Block Organisation at the block level representing 5,000-7,000 women.

At the Village level, Village Organisation members' main focus was to establish functional relationships between SHG federation members and the three local government health worker cadres designated as "AAAs": the Accredited Social Health Activist (ASHA), a community health worker who gets paid based on her ability to mobilize pregnant and recently delivered women to seek recommended health services and promote institutional deliveries; the Auxillary Nurse Midwife (ANM), a trained midwife who organizes monthly health clinics in each village and supervises the ASHA; and the Anganwadi Worker (AWW), a local nutrition worker who operates a day care center and distributes supplementary food for eligible children and pregnant and lactating women.

At the Block level, Village Organization members are voted into the Block Organisation. Their roles were to develop relationships with the supervisors of the village level health workers, medical staff working at the primary health care centres and other block level government functionaries and elected office holders.

For peer review only - http://bmjopen.bmj.com/site/about/guidelines.xhtml

Study design

We developed a pre-test, post-test social network survey. The study objective was to assess how the health coordination and emergency referral networks between women's SHGs and local health systems changed over the course of the two-year Learning Phase of the UPCMP. The survey instruments were based on a validated survey design structure [24] and questions were developed and then pre-tested in a social and cultural setting similar to the study population to capture aspects of village and block level connections that would be relevant for assessing the program. One of the main survey questions asked every respondent whether they coordinated health services, including emergency referrals, with every other respondent type in the survey (Table1).

Study sample

The data were collected through a purposeful sampling methodology that included SHG members and those participating at the federated Village Organisation and Block Organisation levels, government and private health workers, RGMVP staff and other key stakeholders. Block level respondents were only interviewed about their relationship with respondents from two of the six Gram Panchayats to reduce the length of the interviews for block level respondents.

Certain roles are unique in a village or block, such as the ASHA, ANM, Pradhan or village leader. For those roles, the inclusion criteria were that the respondent agree to the interview, be over age 18, and be the person responsible for that role at its respective

For peer review only - http://bmjopen.bmj.com/site/about/guidelines.xhtml

geographic level. For roles that had multiple potential representatives at a given level, survey staff made a list of all potential respondents by consulting with staff at health facilities within each Block, RGMVP program staff, and local stakeholders in each village, and attempted to contact them in a random order. The first potential respondent that was successfully contacted was interviewed. The total sample was 596, with 316 respondents in the baseline and 280 in the endline for response rates of 94% and 82%, respectively.

Table 1 presents the complete list of respondents along with their role designations, the acronyms used in SNA visual plot construction, village or block location, and a brief summary of their respective roles. The respondents were grouped into four broad categories corresponding to their affiliation: SHG Structure, RGMVP Staff, Government Health & Nutrition, and "Other" key stakeholders. This approach incorporates elements of both relational and positional models for examining networks of relations with the network structure.[25]

Table 1: Study Respondents Defined

Acronym (used in SNA plots)	Respondent	Definition
**SHG Structure**		
GM	SHG member	Part of SHG group, involved in saving and borrowing, weekly SHG meetings
G1SS & G2SS	SHG Swasthya Sakhi (two per village)	SHG member who has received special training in health
VOSS	Village Organisation Swasthya Sakhi	SHG leader at village level trained in health and linkages strategy to coordinate health activities
VOM & VOM2	Village Organisation member (two per village)	Leader from 2 village VOs trained in linkages strategy to coordinate health activities
VOB	Village Organisation office bearer	SHG member who holds a post at the federation of SHGs at the village level called Village Organisation

For peer review only - http://bmjopen.bmj.com/site/about/guidelines.xhtml

Acronym (used in SNA plots)	Respondent	Definition
MS	Meeting Sakhi	SHG member who organizes weekly SHG meetings
BOB	Block Organisation office bearer	SHG and Village Organisation member who holds a post at the federation level called Block Organisation
BOR	Block Organisation representative from VO	A member of the Bock Organisation (BO) representing her village organisation
BOPR	Block Organisation Poverty Reduction Committee Member	A dedicated committee in the Block Organisation responsible for development strategies on mobilizing women from poor households into SHGs
**RGMVP Staff**		
RG	RGMVP	Full time field staff who works for the NGO (RGMVP) at block level
RGF	RGMVP Field Officer	Full time field staff who acts as a link between RGMVP and SHGs
RGV	RGMVP Volunteer (Community Volunteer)	A part-time volunteer from the community, who supports the full time field staff in implementation activities
RGT	RGMVP Trainer	RGMVP professional trainer who conducts field trainings for SHGs
ISC	Internal Social Capital	Nominated SHG member who is trained to support health implementation in villages by facilitating discussions in various SHG meetings
**Government Health & Nutrition**		
ASHA	Accredited Social Health Activist	Community health volunteer paid honorarium to promote basic preventative maternal and child services and is supervised by ANM
ANM	Auxiliary Nurse Midwife	Government trained community health worker who conducts monthly outreach clinics providing Maternal and Child care. ANM supervises ASHA and works with AWW
AWW	Anganwadi Worker	Government community worker providing food supplements to young children, adolescent girls, and lactating women along with preschool child education services in government operated village creches
ANMs/LHV	Auxiliary Nurse Midwife Supervisor/ Lady health visitor	ANMs who have been promoted to oversee six sub-centers covering a population of 30,000
ICDSs	Integrated Child Development Scheme Supervisor	Government worker appointed who supervises 25 village level creches called Anganwadi Centers (AWCs) where the AWW works
CDPO	Community Development Program Officer	Government worker in-charge of the ICDS Project at block level who oversees the ICDS supervisor at block level
BDO	Block Development Officer	Government social worker appointed under the ICDS scheme and entrusted with the overall responsibility of the ICDS at block level.
PHM	Primary Health Center Medical Officer	Government Medical Doctor & primary administrator of Primary Health Center (PHC)
PHN	Primary Health Center Nurse	Government Nurse providing nursing services in PHC
PHC	PHC staff	Approximately 13 Government staff appointed to administer primary care services at PHC
PHS	PHC Other	Extra support staff appointed at high delivery load PHCs
CHN	Community Health Center Nurse	Government Nurse appointed at Community Health Centers (CHCs) which constitute the secondary level of health care
CHM	Community Health Center Medical Officer	Government Medical officer who works at CHC to provide clinical services
CHC	CHC Staff	The total strength of staff (approx. 46) appointed by the government to administer specialist and referral care services at CHC
CMO	Chief Medical Officer-in-Charge	Government Medical Doctor who works as the Program director at the District level
CHS	CHC Other	Extra staff appointed at high patient load CHCs
DHN	District Hospital Nurse	Government Nurse working in 300 bed District Hospital for providing comprehensive secondary health care
DHM	District Hospital Medical Officer	Government Medical officer who works at District hospital to provide clinical services
DHOB	District hospital OB/GYN	Government Medical Specialist appointed at District hospital to provide specialist Obstetrics and Gynecology care for women
RKS	Rogi Kalyan Samiti	A patient welfare committee set up at government hospital to ensure accountability and protection of patients' rights

For peer review only - http://bmjopen.bmj.com/site/about/guidelines.xhtml

Acronym (used in SNA plots)	Respondent	Definition
**Other Stakeholders**		
PRI	Pradhan or Village Head	Elected head of a village-level constitutional body of local self-government called the panchayat who acts as focal point of contact between government officers and the village community
RL	Religious Leader	Leader of a religion recognized at the community (village) level
RMP	Rural Medical Practitioner (unqualified)	An unqualified medical practitioner who is not formally trained in providing health care services in villages. RMPs are culturally recognized and often first point of health care in communities
Dr	Medical Doctor (qualified)	Formally trained & accredited Medical Doctor that provides health care
PF	Private Health Facility Provider	Accredited medical doctor providing health services in a private health facility
DS	Drug Shop Owner	Owner of a private chemist shop in villages who dispenses drugs and health advice
BPRI	Block Panchayat Raj Institution	The middle tier of the system of local self-government in India operating at administrative level of block that links villages with Districts

* Technical Support Unit (TSU) project workers: TSUC – TSU Community Specialist; TSUN – TSU Nurse Mentor; TSUB – TSU Block Coordinator

Data collection

The baseline data were collected between November 2013 - January 2014 and endline surveys were adminstered between October - November 2015. Surveys were developed in CSPro 6.0 and adminstered using electronic tablets. During both data collection periods, the survey team spent two weeks in each of the three survey districts. Each team had a supervisor that monitored data quality and a UPCMP project representative that provided logistical support.

Analytic structure and network measures

In consideration of the models to guide network measurement and analysis at the individual and whole network level, our work fits into Mays and Scutchfield's (2010) categorization including: degree centrality, density, and centralization.[13] These measures are used to assess changes in health services coordination networks within the Gram Panchayat level and between key players comprising Gram Panchayat -Block

For peer review only - http://bmjopen.bmj.com/site/about/guidelines.xhtml

relationships. *Degree centrality* measures the number of connections or ties that each respondent maintains [26] and our analysis is based on a mutual confirmation process. In other words, if one person acknowledges a relationship and the other person does not, that relationship is dropped from the network. Overall, 31% of ties remained after the confirmation process. The high loss of unconfirmed ties signals a weak level of connectivity whereas ties confirmed by both parties have a higher probability of producing collaborative relationships.[26] In India, the caste system exerts barriers to relationship formation and contributes to a reduction in reciprocity of ties. *Density is* often used as a measure of social capital [11] and is defined as a ratio of existing relationships or ties in comparison to the potential number of linkages.[27] *Centralization* is an expression of how tightly the network structure is organized around its most central point. The general procedure is to calculate the differences between the centrality scores of the most central point and those of all other points to generate a ratio of the actual sum of differences to the maximum possible sum of differences.[27]

Data analysis

Social network analysis methods have been used to study the structural makeup of cooperation that can lead to stronger collaborative relationships.[28-29] The analysis utilized a combination of two software tools: "R" Version 3 (www.r-project.org),[30] and UCINET Version 6.[31] The plot visualization was developed by using NetDraw.[32]

Patient and public involvement

For peer review only - http://bmjopen.bmj.com/site/about/guidelines.xhtml

No patients or members of the public were involved in the development of research questions, the design of the study, or the development of outcome measures. Also, no patients were asked to advise on interpretation or writing up of results. The study was approved by the Institutional Review Board of Boston University Medical Campus: IRB Number H-32603. A consent form (approved by Boston University Ethics Review Board) was read to participants and required a verbal approval.

Results

Descriptive

The characteristics of study respondents for the two study periods are presented in **Table 2**. Most demographic indicators were balanced across baseline and endline rounds using a chi-square test, except for caste ($p<0.001$). For this indicator, Scheduled Caste and Scheduled Tribe accounted for a greater share of respondents in the baseline compared with the endline, while Other Backward Caste representation was greater in the endline. The median age of the respondents was approximately 40 years. About a quarter to a third of all respondents were not educated. In both rounds of the survey, more than 50% of SHG-level respondents reported having no formal education, while all government health workers reported some education and approximately one third reported post-graduate studies. Four district level respondent roles were added in the endline.

For peer review only - http://bmjopen.bmj.com/site/about/guidelines.xhtml

Table 2: Comparison of Respondent Background Information

Characteristic	UP CMP Baseline 2013 (Banda, Mirzapur, Hardoi) N (%)	UP CMP Endline 2015 (Banda, Mirzapur, Hardoi) N (%)
**Median Age**	41	40
**Education**		
- No Education	100 (32)	74 (26)
- Some Primary (up to 8th standard)	63 (20)	74 26)
- Some Secondary (up to 11th standard)	40 (13)	40 (14)
- Secondary Completed (12th standard)	2 (1)	3 (1)
- Post-graduate (at least some college completed)	111 (35)	89 (32)
**Caste[1*]**		
- Scheduled Caste / Scheduled Tribe	172 (55)	106 (38)
- Other Backward Castes	71 (22)	112 (40)
- General Caste	71 (22)	62 (22)
**Affiliation**		
- SHG Structure	119 (38)	98 (35)
- RGMVP Staff	23 (7)	24 (9)
- Gov Health	77 (24)	77 (28)
- Other	97 (30)	81 (29)
**Location**		
- Purwa (Hamlet)	34 (11)	33 (12)
- Gram Panchayat (Village)	220 (70)	203 (73)
- Block	62 (20)	34 (12)
- District	0	10 (4)
**TOTAL**	**316**	**280**

The classification of castes is formalized by the Government of India into these categories and we used standard definitions to create these categories. Some of these lower caste designations enable caste groups to receive specific government benefits and subsidies. Respondents were asked to self-identify into caste categories in the survey.
* Chi Square test: $p<0.001$

Respondents were also asked whether they had friends or neighbors who were SHG members, and these results are presented **in Table 3**. The percentage of SHGs in the federated structure that knew other SHG members between the baseline and endline increased significantly from 87% to 100%. The majority of RGMVP staff respondents knew SHG members in their Gram Panchayat or village. Only about one third of government health workers knew an SHG member, while almost two thirds of

For peer review only - http://bmjopen.bmj.com/site/about/guidelines.xhtml

respondents from the “Other” category (including the Pradhan (local leader), Drug Shop Owner, Traditional Birth Attendant etc.) knew an SHG member.

Table 3: Respondent with Friends or Neighbors who are SHG Members by “Affiliation” (row percentages)

	UP CMP – 2013 (Banda, Mirzapur, Hardoi) N=316			UP CMP – 2015 (Banda, Mirzapur, Hardoi) N=280		
**Affiliation**	**No (%)**	**Yes (%)**	**Don’t Know (%)**	**No (%)**	**Yes (%)**	**Don’t Know (%)**
**SHG Structure***	16 (13)	103 (87)	0 (0)	0 (0)	98 (100)	0 (0)
**RGMVP Staff**	4 (17)	18 (78)	1 (4)	1 (4)	23 (96)	0 (0)
**Gov Health**	45 (58)	23 (30)	9 (12)	46 (60)	26 (34)	5 (6)
**Other**	33 (34)	58 (60)	6 (6)	22 (27)	53 (65)	6 (7)
**Total**	95 (31)	202 (64)	16 (5)	69 (25)	200 (71)	11 (4)

* Fischer’s Exact Test: p-value <0.0001. All other rows insignificant.

Social Network Analysis

A fixed set of Gram Panchayat and block level providers and other key community members involved in health care delivery were included in the analysis. Network measures for density, centralization and average degree centrality are presented individually for all 18 villages (6 per district) as well as the average scores across each district for the baseline and endline (**Table 4**). However, since block level respondents were asked about relationships with only two Gram Panchayats, we confirmed whole Gram Panchayat-block relationships for these two Gram Panchayat-block dyads in each of the three blocks along with results for baseline and endline (**Table 5**).

Network metrics

For peer review only - http://bmjopen.bmj.com/site/about/guidelines.xhtml

Density of the overall village health services and referral networks in 18 Gram Panchayats (villages) were low but increased from baseline to endline in two out of three districts: Banda (7.5% to 10.1%) and Mirzapur (4.7% to 12.6%). Hardoi had a higher density at baseline compared to the other two blocks and had a slight reduction at endline (13.6% to 11.4%). While the average patterns of density change in blocks during the two year intervention period increased, variability was noted beween individual villages in each of the blocks related to their baseline characteristics, and the degree of change that occurred between baseline and endline. Although Mirzapur's density was the lowest in the Gram Panchayats measured, they had the greatest growth, as noted by tripling of their scores during the two year UPCMP intervention. Hardoi had a reduction in density to half of Gram Panchayats but remained within a close range to the other two blocks at endline.

The Gram Panchayat-block level health services and referral networks take into account the relationships between two villages and block level respondents within each block **(Table 5)**. The baseline densities were lower overall than village level networks but increased after two years of project implementation, although the level of increase was less than in the village level networks. Similar to village level network patterns, Hardoi's Gram Panchayat-block level health services and emergency referral network remained at the highest level in comparison to the other two blocks in both baseline and endline, but decreased slightly in the endline (12.4 to 10.5).

For peer review only - http://bmjopen.bmj.com/site/about/guidelines.xhtml

The centralization measures increased two to three-fold during the same two year intervention period, with the highest level of increase in Mirzapur Gram Panchayats, which doubled on average (15.1% to 29.2%). Within the overall average increase in all blocks, Gram Panchayat-level variation was noted. This included a decrease in centralization in two Banda villages and four Hardoi villages. Centralization in the Gram Panchayat-Block networks also increased in all three blocks with the largest gains in Mirzapur (8.9% to 23.9%), while Banda increased from 18.5% to 29.0% and Hardoi from 28.9% to 30.1%.

Mirzapur and Banda had the highest increase in average degree centrality but endline results suggest similar levels of provider, SHG, key health provider, and community contacts in all three blocks with 3.1 to 3.6 contacts per person. Mirzapur started at the lowest level with only 1.5 confirmed contacts compared to Banda with 2.3 and Hardoi at 4.1, with variability at the individual village level ranging from 0.8 to 6.5 across the three blocks. All Gram Panchyat-block level measures had higher levels of contacts at baseline compared to Gram Panchayat levels with Hardoi reporting a high of 7.7 contacts. Endline average degree centrality measures in Gram Panchayat-block dyads in Banda, Mirzapur and Hardoi were 5.0, 5.2 and 7.2, respectively.

Table 4: Sociometric Gram Panchayat Level Coordination and Referral Networks: Confirmed Density, Centralization and Average Degree Centrality

**District** (1 Block per District)	**Confirmed Density (%)**		**Confirmed Centralization (%)**		**Avg. Degree Centrality**	
	Baseline	Endline	Baseline	Endline	Baseline	Endline

For peer review only - http://bmjopen.bmj.com/site/about/guidelines.xhtml

**District** (1 Block per District)	**Confirmed Density (%)**		**Confirmed Centralization (%)**		**Avg. Degree Centrality**	
**Banda Gram Panchayats**						
1	10.0	7.5	17.8	13.3	3.0	2.3
2	5.8	8.3	15.1	33.8	1.8	2.5
3	10.8	16.7	31.1	39.1	3.3	5.0
4	4.2	5.8	16.9	15.1	1.3	1.8
5	7.5	13.3	20.4	28.4	2.3	4.0
6	6.7	9.2	14.2	18.7	2.0	2.8
Average	*7.5*	*10.1*	*19.3*	*24.7*	*2.3*	*3.1*
**Hardoi Gram Panchayats**						
7	11.7	4.4	23.1	8.6	3.5	1.4
8	19.2	19.1	36.4	32.8	5.8	6.1
9	21.7	15.4	26.7	23.4	6.5	4.9
10	6.7	8.1	14.2	37.9	2.0	2.6
11	11.7	6.6	16.0	19.5	3.5	2.1
12	10.8	14.7	31.1	30.9	3.3	4.7
Average	*13.6*	*11.4*	*24.6*	*25.5*	*4.1*	*3.6*
**Mirzapur Gram Panchayats**						
13	5.8	11.7	15.1	30.2	1.8	3.5
14	2.5	8.3	11.6	33.8	0.8	2.5
15	5.8	12.5	15.1	22.2	1.8	3.8
16	3.3	19.2	10.7	50.7	1.0	5.8
17	8.3	8.3	26.7	19.6	2.5	2.5
18	2.5	15.8	11.6	18.7	0.8	4.8
Average	*4.7*	*12.6*	*15.1*	*29.2*	*1.5*	*3.8*

Table 5: Sociometric Gram Panchayat (GP)-Block Level Coordination and Referral Networks: Confirmed Density, Centralization and Average Degree Centrality

**District** (1 Block per District)	**Confirmed Density (%)**		**Confirmed Centralization (%)**		**Avg. Degree Centrality**	
	Baseline	Endline	Baseline	Endline	Baseline	Endline
**Banda**						
GP 2 + Block	7.1	7.4	12.7	34.3	4.4	4.7
GP 5 + Block	5.4	8.3	24.3	23.6	3.4	5.3
Average	6.3	7.9	18.5	29.0	3.9	5.0
**Hardoi**						

For peer review only

**District** (1 Block per District)	**Confirmed Density (%)**		**Confirmed Centralization (%)**		**Avg. Degree Centrality**	
GP 8 + Block	11.1	10.9	25.2	34.2	6.9	7.4
GP 9 + Block	13.7	10.1	32.5	26.0	8.5	6.9
Average	12.4	10.5	28.9	30.1	7.7	7.2
**Mirzapur**						
GP 13 + Block	3.4	8.1	9.8	23.8	2.1	5.2
GP 14 + Block	2.0	8.0	7.9	24.0	1.3	5.1
Average	2.7	8.1	8.9	23.9	1.7	5.2

Health services and emergency referral networks

The structures of the health services coordination and emergency referral networks were visualized for all 18 Gram Panchayats (villages) and for the six Gram Panchayat-Block dyads. We have selected one example from each Gram Panchayat-Block network to illustrate the diversity of changes that occurred in structure and network connectivity. The nodes are labelled by respondent, color-coded to reflect the type of respondent group, and shaped according to the location of the respondent's usual residence or work location. The nodes are sized by betweeness centrality with larger nodes signifying the increasing importance of that respondent in linking others that may not be directly connected to each other.

The structure and the relationships between different individuals and groups changed between baseline and after two years of UPCMP implementation. At baseline, the Mirzapur Gram Panchayat-Block health services and referral network had a "kite-like" structure with only two of the four respondent groups in the network (government health and social workers and the SHG structure) having relationships with no block level connections (**Figure 2a**). At one end, a triad of the three village health and social

For peer review only

workers (ASHA, ANM and AWW) were connected together. The AWW had a relationship with an SHG member but had no direct connections with the SHG federated structure. The SHG structures themselves (at the tail end of the kite-like structure) were not integrated. At endline (**Figure 2b**), the network structure expanded into a series of connected sets of relationships with the expansion of the number of participants and the inclusion of the traditional birth attendant (TBA) and RGMVP's Block level Internal Social Capital worker (ISC). The shift from the AWW, a role more focused on nutrition and child development, to the ASHA as the main connector to the SHG structure at two critical points, directly to an SHG member as well as the SHG Village Organisation's Swasthya Sakhii (VOSS), expanded the potential for better health services coordination. The SHG structure also became better integrated with the Village Organisation level functionaries linked together with the exception of the Village Organisation Swasthya Sakhi.

At baseline, the Banda Gram Panchayat-Block level health services and referral network was organized into two small worlds, with the SHG structure and the RGMVP SHG support structure in one cluster while the government health system and a few other key village respondents were in another separate cluster (**Figure 3a**). Within the SHG small world cluster, RGMVP was at the center of a centralized system resembling a "spoke and wheel" with members of the SHG structure at the village and block levels connecting to RGMVP but not to each other.

For peer review only - http://bmjopen.bmj.com/site/about/guidelines.xhtml

In the second cluster, the ASHA was a leading connector of village level respondents (AWW, the rural medical practitioner (RMP), an unqualified provider and an SHG member) to the broader government health system at the Block level (ANM supervisor, primary health center medical officer (PHM) and the village oriented ANM provider). By the endline, the two separate clusters formed one network (**Figure 3b**). The ASHA became the main connector between the government health providers and the SHG structure. The SHG Village Organisation members also increased coordination within their own network and RGMVP. RGMVP scaled back from their centralized role at baseline and moved to the periphery of the network while SHG enagagment increased.

In Hardoi, the structure remained similar since they started with one whole network, however an additional project, the Technical Support Unit (TSU), a supply side intervention working with the health system, was just starting during the endline collection period (**Figures 4a – 4b**). The ANM held the central position in the endline heath services and emergency referral coordination network and acted as an intermediary between the government health functionaries at the block level and the SHG federated structure. The ASHA also stood out as more direct connectivity was established with different members of the SHGs. Within the SHG platform itself, there were more channels of direct communication between different members and women in designated leadership positions.

For peer review only - http://bmjopen.bmj.com/site/about/guidelines.xhtml

Discussion

After two years of UPCMP project implementation, the health services coordination and emergency referral networks increased in their density but absolute levels remained low. This was a positive direction as higher density is considered an overall indicator of cohesion and interaction within a network and is often associated with faster rates of information diffusion within a community.[11] As information is more quickly shared, processed and appropriated, it can lead to shared decision making.[33]

However, our study results have limitations related to the constraints of social network analysis, minimal prior research on coordination networks between SHGs and government, and limited duration of testing the full effects of the linkages intervention. SNA has been mainly used for descriptive purposes and has been less frequently used for evaluating interventions [34] and therefore needs further testing to determine broader generalizability. Sources of bias may include using purposive sampling to identify the respondent roles in the villages and blocks. Additionally, there are no established criteria for evaluating networks [35] as SNA represents a relatively new multi-disciplinary methodology with limited empirical studies. While there is evidence in the literature that the constructs of density and network centrality may influence partnership functioning and coordination, their magnitudes and mechanisms of effect in public health are still largely unknown.[13] Our study provides more empirical evidence to further explore the potential of SNA to measure coordination networks.

For peer review only

Our study demonstrated an increase in connections not only between individuals but also an expansion of relationships between groups. Poor and traditionally lower-caste SHGs developed skills to cross boundaries and forge relationships with health providers. Traditionally bound by societal and cultural expectations, it has been difficult to overcome social and structural barriers, which limit interactions among heterogenous groups. A strategy based on increasing diversity in relationships while actively working to reduce redundancy can lead to improved levels of bridging social capital in a network. Such an approach that connects essential groups can lead to better ways of coordinating and collaborating.[11] Therefore, SHGs with increasing skills and confidence in engaging with health services providers are creating in-roads to better health services for themselves and their communities.

As proxies for bridging social capital, the average degree centrality increased by creating new connections within the two years of UPCMP implementation. However, simply increasing the number of ties may not necessarily result in strong relationships, as noted by the weak ties theory.[36] Although it is common to surround ourselves with strong ties that include people similar to us in beliefs, values, and access to resources, it is through weak ties that we begin to diversify our networks and create avenues for accessing more varied resources.[11] It is probable that building SHG leadership and coordination skills, which was a major UPCMP intervention, facilitated a pathway for

For peer review only - http://bmjopen.bmj.com/site/about/guidelines.xhtml

SHGs to create weak ties with the health system. These patterns reflect opportunities for diverse stakeholders to engage in the planning and production of change.[37]

Our analysis showed that the ASHA occupied the central position in the network and her role expanded to coordinating services with the Village Organization Swasthya Sakhi and others in the SHG federation. The ASHA is a community health volunteer and her role evolved from an "activist" and advocate of her village to being part of the government health system and being accountable to the ANM or nurse midwife as well as the Pradhan (leader) in the Village Health, Nutrition and Sanitation Committee. As an important and recognized member of the community, the ASHA's modeling of cross-caste and class relationships can influence the governance dynamics as building collaborative networks are considered a more democratic means of developing public policy.[38-41]

Conclusions

The SHG platform and its federated structure was developed by RGMVP to scale-up and galvanize SHG development to reduce poverty, support women's empowerment, and break caste based hierarchies to encourage comprehensive rural development. The endline social network analysis reveals an expansion of pathways to coordinate health services and emergency referrals for poor, illiterate, and low caste women through the added voices of SHG members. The ability of women with multi-generational social and economic deprivations to broaden their exposure to social networks through SHG

For peer review only - http://bmjopen.bmj.com/site/about/guidelines.xhtml

membership is a major step towards gaining self-confidence in participatory community development.

The increasing success of SHG federatations to forge linkages with the health system led to greater coordination for health services delivery while stimulating a more centralized structure of core village health workers. Collaborative processes that include individual empowerment, bridging social ties, and synergy can strengthen the capacity to solve problems.[42] These ideas feed into the concept of state-society syngery where a mobilized civil society and an active government can work together to build social capital and enhance each other's development efforts.[11,43]

If successful, these partnerships can serve as vehicles for transforming public health from a diverse collection of activities and organisations into an organized and accountable delivery system.[13] The challenge will be in maintaining these networks, so that they remain dynamic and offer new benefits to partners.[24] Future studies that include longitudinal data can provide deeper understanding of the mechanisms by which intersectoral partnerships and community mobilization lead to effective coordination networks to tackle health problems and their socioeconomic determinants.

For peer review only - http://bmjopen.bmj.com/site/about/guidelines.xhtml

Citations/References

[1] Microfinance Summit Campaign. Mapping Pathways out of Poverty: The State of the Microcredit Summit Campaign Repor, 2015.
Link: https://stateofthecampaign.org/read-the-full-2015-report/

[2] Leatherman S, Metcalfe M, Geissler K, Dunford C. Integrating microfinance and health strategies: examining the evidence to inform policy and practice. *Health Policy Plan* Feb 22 2011;27(2):85-101.
Link: https://doi.org/10.1093/heapol/czr014

[3] Ruducha J, Jadhav M. A review of organizational arrangements in microfinance and health programs. *J Glob Health Rep* 2018 Jul;25;2.
Link: http://www.joghr.org/Article/joghr-02-e2018024

[4] Messiana DN. Social Networking of Self-Help Groups in Tribal Villages of Srikakulam District of Andhra Pradesh – A critical Analysis (Doctoral Disseration), *Acharya NG Ranga Agricultural Unvierisity,* 2012
Link: http://krishikosh.egranth.ac.in/displaybitstream?handle=1/67004

[5] Reddy CS, Manak S. Self-help groups: A keystone of microfinance in India-women empowerment and social security. *Andhra Pradesh Mahila Abhivruddhi Society (APMAS) India: Hyderabad* Oct 2005.
Link: http://www.arabic.microfinancegateway.org/sites/default/files/mfg-en-paper-self-help-groups-a-keystone-of-microfinance-in-india-women-empowerment-social-security-oct-2005.pdf

[6] Garikipati S. The impact of lending to women on household vulnerability and women's empowerment: evidence from India. *World Development* Dec 1 2008;36(12):2620-42.
Link: https://doi.org/10.1016/j.worlddev.2007.11.008

[7] Unifem. Progress of the World's Women 2000. Biennial Report, United Nations Development Fund for Women, New York, 2000.
Link: http://www.unwomen.org/en/digital-library/publications/2000/1/progress-of-the-world-s-women-2000

For peer review only - http://bmjopen.bmj.com/site/about/guidelines.xhtml

[8] IFAD. Enabling the Rural Poor to Overcome Poverty, 2003.
Link: https://www.ifad.org/documents/38714170/39309792/e_1.pdf/c75a166d-ecee-46af-abd3-0da951504970

[9] Anderson L. Self-Help Groups in Development: A Review of Evidence from South Asia and Sub-Saharan Africa. Evans School of Public Affairs, University of Washington. Prepared for the Strategy, Measurement and Evaluation Team of the Bill and Melinda Gates Foundation, 2014.
Link: https://evans.uw.edu/sites/default/files/EPAR_283_SHG%20Evidence%20Review%20Brief_12.5.14_0.pdf

[10] Nowell B. Profiling capacity for coordination and systems change: The relative contribution of stakeholder relationships in interorganizational collaboratives. *Am J Community Psychol* Dec 1 2009;44(3-4):196-212.
Link: https://doi.org/10.1007/s10464-009-9276-2

[11] Varda DM. A network perspective on state-society synergy to increase community-level social capital. *Nonprofit Volunt Sect Q* Oct 2011;40(5):896-923.
Link: https://doi.org/10.1177/0899764010378171

[12] Putnam RD. The prosperous community. *Am Prospect* Mar 21 1993;4(13):35-42.
Link: http://staskulesh.com/wp-content/uploads/2012/11/prosperouscommunity.pdf

[13] Mays GP, Scutchfield FD. Improving public health system performance through multiorganizational partnerships. *Prev Chronic Dis* Nov 2010;7(6).
Link: https://www.ncbi.nlm.nih.gov/pmc/articles/PMC2995603/

[14] Gulati R, Gargiulo M. Where do interorganizational networks come from?. *Am J Psychol Mar* 1999;104(5):1439-93.
Link: https://doi.org/10.1086/210179

[15] Isett KR, Provan KG. The evolution of dyadic interorganizational relationships in a network of publicly funded nonprofit agencies. *J Public Adm Res Theory* Jan 1 2005;15(1):149-65.
Link: https://doi.org/10.1093/jopart/mui008

For peer review only - http://bmjopen.bmj.com/site/about/guidelines.xhtml

[16] Mandell MP, editor. Getting results through collaboration: Networks and network structures for public policy and management ABC-CLIO, 2001.

[17] Monge PR, Fulk J, Kalman, ME., Flanagin AJ, Parnassa C, Rumsey S. Production of collective action in alliance-based interorganizational communication and information systems. *Organization Science* 1998;9:411-433.
Link: https://doi.org/10.1287/orsc.9.3.411

[18] Westley F, Vredenburg H. Interorganizational collaboration and the preservation of global biodiversity. *Organization Science* 1997;8:381-403.
Link: https://doi.org/10.1287/orsc.8.4.381

[19] Witte JM, Reinicke W, Benner T. Networked governance: Developing a research agenda. Paper presented at the *Annual Convention of the International Studies Association Meetings*, New Orleans, LA, 2002.

[20] Jones PS. Urban regeneration's poisoned chalice: is there an impasse in (community) participation-based policy? *Urban Stud* 2003;40(3):581-601.
Link: https://doi.org/10.1080/0042098032000053932

[21] Karim L. Demystifying micro-credit: the Grameen Bank, NGOs, and neoliberalism in Bangladesh. *Cultural Dynamics* Mar 2008;20(1):5-29.
Link: https://doi.org/10.1177/0921374007088053

[22] Kothari U, Cooke B (editor). Power, knowledge and social control in participatory development. In *Participation: The New Tyrann*y? Zed Books, London2001;139-52.

[23] Registrar General I. Census of India 2011: provisional population totals-India data sheet. Office of the Registrar General Census Commissioner, India. *Indian Census Bureau* 2011.

[24] Provan KG, Veazie MA, Staten LK, Teufel-Shone NI. The Use of Network Analysis to Strengthen Community Partnerships. *Public Adm Rev* 2005;65:603–613.
Link: https://doi.org/10.1111/j.1540-6210.2005.00487.x

[25] Burt RS. Brokerage and closure: An introduction to social capital. Oxford University Press, 2005.

For peer review only - http://bmjopen.bmj.com/site/about/guidelines.xhtml

[26] Freeman LC. Centrality in social networks conceptual clarification. *Soc Networks* 1978;1(3):215-39.
Link: https://www.bebr.ufl.edu/sites/default/files/Centrality%20in%20Social%20Networks.pdf

[27] Hanneman R, Riddle M. Introduction to Social Network Analysis. *Riverside CA: University of California,* Oct 1 2005.
Link: http://faculty.ucr.edu/~hanneman/nettext/

[28] Scott J. Social Network Analysis. Sage, 2017

[29] Wasserman S, Faust K. Social network analysis: Methods and applications. Cambridge University Press, 1994.

[30] Team RC. R: A language and environment for statistical computing.
Link: www.r-project.org

[31] Borgatti SP, Everett MG, Freeman LC. UCINET for Windows: Software for social network analysis [computer software]. Natick, MA: Analytic Technologies, 2002.

[32] Borgatti SP. NetDraw Software for Network Visualization. Analytic Technologies: Lexington, KY, 2002.

[33] Wensing M, Van Lieshout J, Koetsenruiter J, Reeves D. Information exchange networks for chronic illness care in primary care practices: an observational study. *Implement Sci* 2010;5(1):3.
Link: https://doi.org/10.1186/1748-5908-5-3

[34] DeBruin A, McAuliffe E. Social Network Analysis as a methodological approach to explore health systems: A case study exploring support among senior managers/executives in a hospital network. *Int J Environ Res Public Health* 2018;15(3):511.
Link: https://doi.org/10.3390/ijerph15030511

[35] Balkundi P, Kilduff M. The ties that lead: A social network approach to leadership. *Leadersh Q* 2006;17:419–439.
Link: https://doi.org/10.1016/j.leaqua.2006.01.001

For peer review only - http://bmjopen.bmj.com/site/about/guidelines.xhtml

[36] Granovetter MS. The strength of weak ties. *In Social Networks* Jan 1 1977 (pp. 347-367) Academic Press.
Link: https://doi.org/10.1016/B978-0-12-442450-0.50025-0

[37] Butterfoss FD, Goodman RM, Wandersman A. Community coalitions for prevention and health promotion: Factors predicting satisfaction, participation, and planning. *Health Educ Q* 1996;23(1):65-79.
Link: https://doi.org/10.1177/109019819602300105

[38] Powell WW, Koput KW, Smith-Doerr L. Interorganizational collaboration and the locus of innovation: Networks of learning in biotechnology. *Adm Sci Q* 1996;116-45.
Link: https://www.jstor.org/stable/2393988

[39] Jennings Jr ET, Ewalt JA. Interorganizational coordination, administrative consolidation, and policy performance. *Public Adm Rev* 1998;417-28.
Link: https://www.jstor.org/stable/977551

[40] Milward HB, Provan KG. Measuring network structure. *Public Adm* 1998;76(2):387-407.
Link: https://doi.org/10.1111/1467-9299.00106

[41] Provan KG, Milward HB. A preliminary theory of interorganizational network effectiveness: A comparative study of four community mental health systems. *Adm Sci Q* 1995;1-33.
Link: https://www.jstor.org/stable/2393698

[42] Lasker RD, Weiss ES. Broadening participation in community problem solving: a multidisciplinary model to support collaborative practice and research. *J Urban Health* 2003;80(1):14-47.
Link: https://doi.org/10.1093/jurban/jtg014

[43] Evans P. Government action, social capital and development: reviewing the evidence on synergy. *World Development* 1996;24(6):1119-32.
Link: https://doi.org/10.1016/0305-750X(96)00021-6

For peer review only - http://bmjopen.bmj.com/site/about/guidelines.xhtml

FOOTNOTES

Contributers: Conceptualization and Project Design, JR; Methodology, JR and JP; Network Analysis, JP; Qualitative Analysis: JP, JR, DH; Interpretation: JR, JP, DH; Interviewer Training, JP, DH, JR; Field Investigation, DH, DA, SK, PSM, JP; Draft preparation, JR; Literature Review, JR; Revising and Editing, JR, DH, LI, JP, KNGL, DA.

Acknowledgements: The authors would like to thank Dileep Mavalankar for his leadership of the Uttar Pradesh Community Mobilization Project, Deborah Maine, formerly at Boston University Center for Global Health and Development (BU CGHD) who supported the study's new methods and ME Khan, formerly of the Population Council for his knowledge and open discussions and that led to many ideas about how to study rural health systems. Many staff members of the Rajiv Gandhi Mahila Vikas Pariyojana including Gyanendra Verma and Kapil Patil and the Public Health Foundation of India (Anuraag Chaturvedi, Sanjit Sarkar and Srinivasan Soundararajan) assisted in the process of implementing this research project. We also acknowledge the administrative support and data collection oversight provided by Ariel Falconer, formerly at BU CGHD for the baseline and Steven Crimaldi of BU CGHD, for the endline. Lastly, we would like to thank the study respondents who offered their time and information to make this research possible.

Funding: This work was supported by the Bill and Melinda Gates Foundation grant number OPP 1033910.

Data Availability: Additonal unpublished data are available from the corresponding author upon request.

Competing Interests: None declared.

Participant Consent: Consent form (approved by Boston University Ethics Review Board) was read to particiapnts and required a verbal approval.

Ethics Approval: The surveys were approved by the Institutional Review Board of Boston University Medical Campus**:** IRB Number H-32603.

Patient and Public Involvement: No patients or members of the public were involved in the development of research questions, the design of the study, or the development of outcome measures. No patients were asked to advise on interpretation or writing up of results.

Provenance and Peer Review: Not commissioned

For peer review only

FIGURE CAPTIONS:

Figure 1: Map of Three Study Districts in Uttar Pradesh, India

Figure 2a: Mirzapur GP-Block Coordination Network - Baseline

Figure 2b: Mirzapur GP-Block Coordination Network - Endline

Figure 3a: Banda GP-Block Coordination Network - Baseline

Figure 3b: Banda GP-Block Coordination Network - Endline

Figure 4a: Hardoi GP-Block Coordination Network - Baseline

Figure 4b: Hardoi GP-Block Coordination Network - Endline

For peer review only

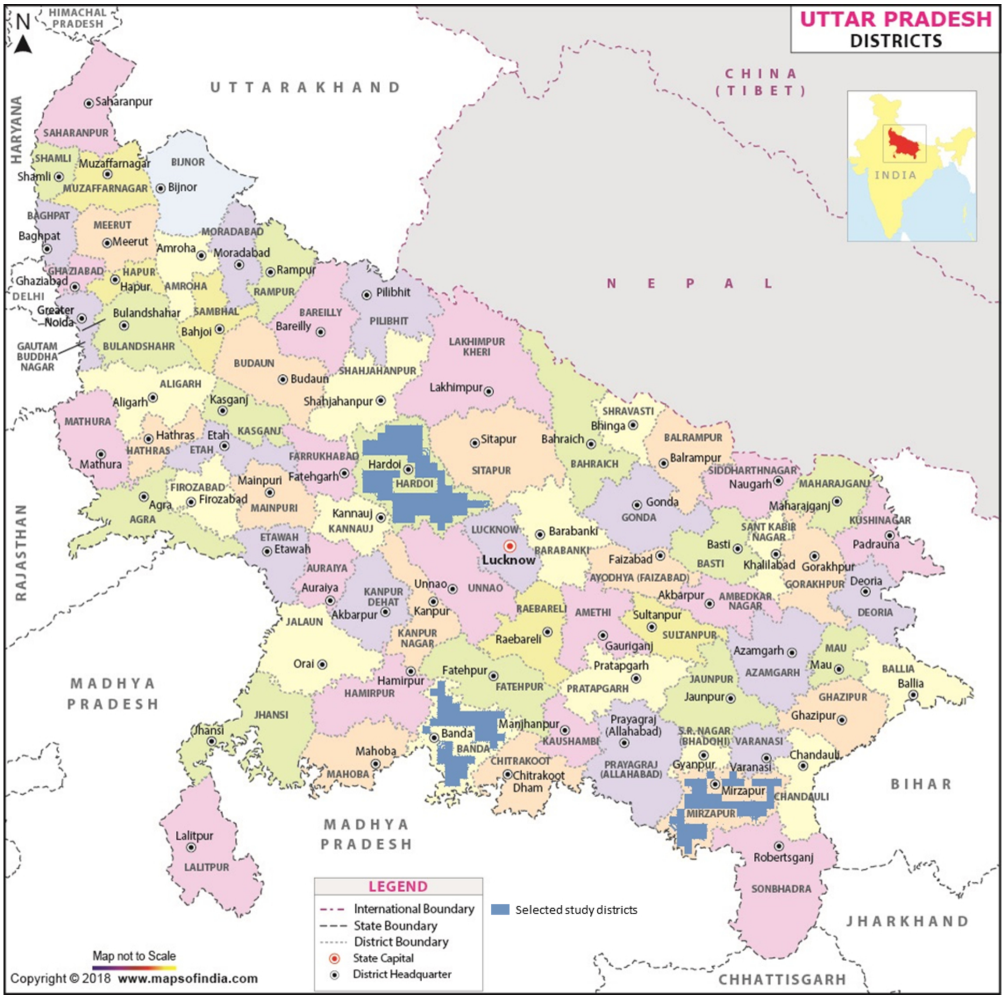

Figure 1: Map of Three Study Districts in Uttar Pradesh, India

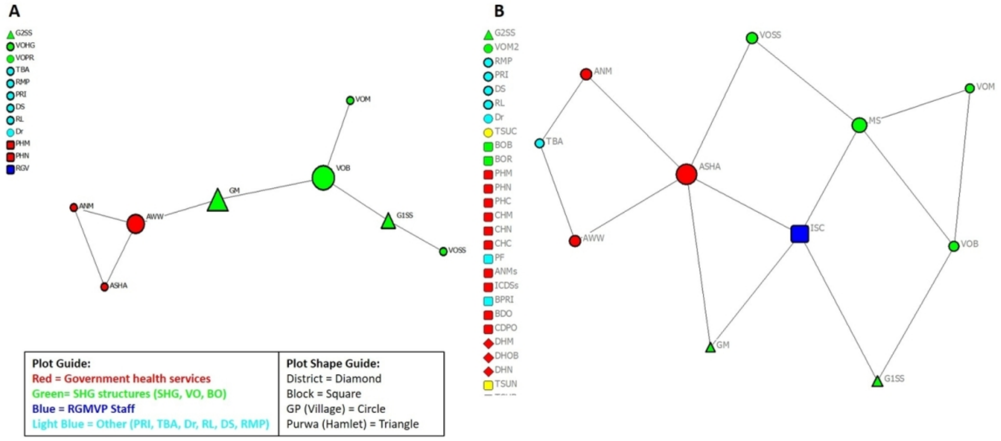

Figure 2a: Mirzapur GP-Block Coordination Network - Baseline
Figure 2b: Mirzapur GP-Block Coordination Network - Endline

62x28mm (600 x 600 DPI)

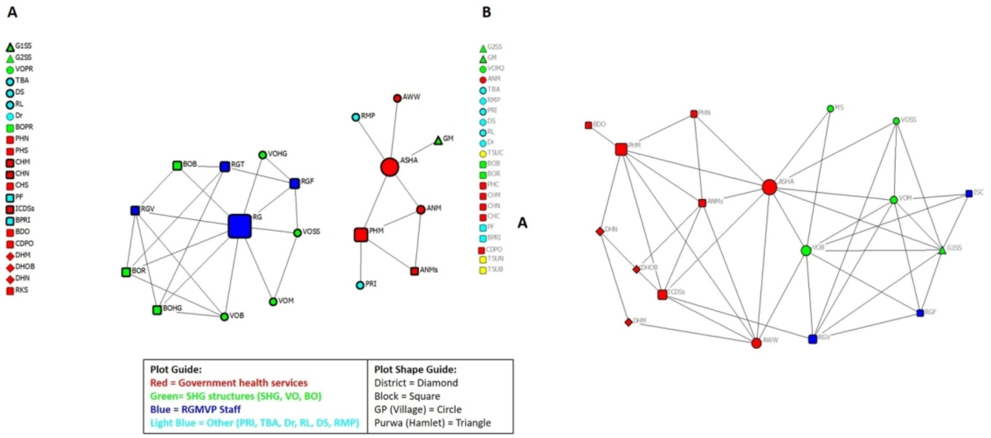

Figure 3a: Banda GP-Block Coordination Network - Baseline
Figure 3b: Banda GP-Block Coordination Network - Endline

71x32mm (600 x 600 DPI)

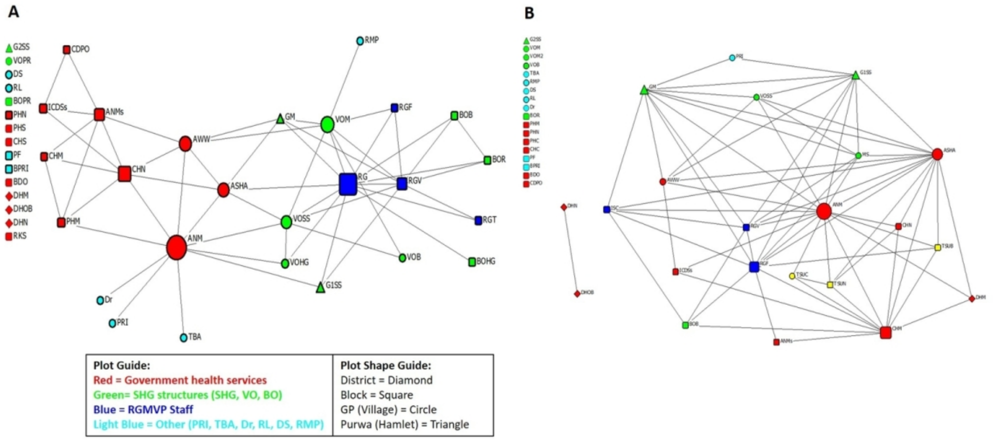

Figure 4a: Hardoi GP-Block Coordination Network - Baseline
Figure 4b: Hardoi GP-Block Coordination Network - Endline

64x29mm (600 x 600 DPI)

Reporting checklist for cross sectional study.

Based on the STROBE cross sectional guidelines.

Instructions to authors

Complete this checklist by entering the page numbers from your manuscript where readers will find each of the items listed below.

Your article may not currently address all the items on the checklist. Please modify your text to include the missing information. If you are certain that an item does not apply, please write "n/a" and provide a short explanation.

Upload your completed checklist as an extra file when you submit to a journal.

In your methods section, say that you used the STROBE cross sectional reporting guidelines, and cite them as:

von Elm E, Altman DG, Egger M, Pocock SJ, Gotzsche PC, Vandenbroucke JP. The Strengthening the Reporting of Observational Studies in Epidemiology (STROBE) Statement: guidelines for reporting observational studies.

		Reporting Item	Page Number
Title	#1a	Indicate the study’s design with a commonly used term in the title or the abstract	1
Abstract	#1b	Provide in the abstract an informative and balanced summary of what was done and what was found	2-3
Background / rationale	#2	Explain the scientific background and rationale for the investigation being reported	4-6
Objectives	#3	State specific objectives, including any prespecified hypotheses	5 & 8
Study design	#4	Present key elements of study design early in the paper	8
Setting	#5	Describe the setting, locations, and relevant dates, including periods of recruitment, exposure, follow-up, and data collection	9 & 11
Eligibility criteria	#6a	Give the eligibility criteria, and the sources and methods of selection of participants.	9-11

For peer review only

	#7	Clearly define all outcomes, exposures, predictors, potential confounders, and effect modifiers. Give diagnostic criteria, if applicable	11-12
Data sources / measurement	#8	For each variable of interest give sources of data and details of methods of assessment (measurement). Describe comparability of assessment methods if there is more than one group. Give information separately for for exposed and unexposed groups if applicable.	12
Bias	#9	Describe any efforts to address potential sources of bias	25
Study size	#10	Explain how the study size was arrived at	11-12
Quantitative variables	#11	Explain how quantitative variables were handled in the analyses. If applicable, describe which groupings were chosen, and why	9-11
Statistical methods	#12a	Describe all statistical methods, including those used to control for confounding	12 & 15
	#12b	Describe any methods used to examine subgroups and interactions	14, 17-18
	#12c	Explain how missing data were addressed	N/A
	#12d	If applicable, describe analytical methods taking account of sampling strategy	N/A
	#12e	Describe any sensitivity analyses	N/A
Participants	#13a	Report numbers of individuals at each stage of study—eg numbers potentially eligible, examined for eligibility, confirmed eligible, included in the study, completing follow-up, and analysed. Give information separately for for exposed and unexposed groups if applicable.	N/A
	#13b	Give reasons for non-participation at each stage	N/A
	#13c	Consider use of a flow diagram	N/A
Descriptive data	#14a	Give characteristics of study participants (eg demographic, clinical, social) and information on exposures and potential confounders. Give information separately for exposed and unexposed groups if applicable.	12-14

For peer review only

	#14b	Indicate number of participants with missing data for each variable of interest	N/A
Outcome data	#15	Report numbers of outcome events or summary measures. Give information separately for exposed and unexposed groups if applicable.	15-17
Main results	#16a	Give unadjusted estimates and, if applicable, confounder-adjusted estimates and their precision (eg, 95% confidence interval). Make clear which confounders were adjusted for and why they were included	N/A
	#16b	Report category boundaries when continuous variables were categorized	N/A
	#16c	If relevant, consider translating estimates of relative risk into absolute risk for a meaningful time period	N/A
Other analyses	#17	Report other analyses done—e.g., analyses of subgroups and interactions, and sensitivity analyses	N/A
Key results	#18	Summarise key results with reference to study objectives	22-26
Limitations	#19	Discuss limitations of the study, taking into account sources of potential bias or imprecision. Discuss both direction and magnitude of any potential bias.	22
Interpretation	#20	Give a cautious overall interpretation considering objectives, limitations, multiplicity of analyses, results from similar studies, and other relevant evidence.	22-26
Generalisability	#21	Discuss the generalisability (external validity) of the study results	3 & 22
Funding	#22	Give the source of funding and the role of the funders for the present study and, if applicable, for the original study on which the present article is based	32

The STROBE checklist is distributed under the terms of the Creative Commons Attribution License CC-BY. This checklist can be completed online using https://www.goodreports.org/, a tool made by the EQUATOR Network in collaboration with Penelope.ai

For peer review only

BMJ Open

Measuring Coordination between Women's Self-Help Groups and Local Health Systems in Rural India: A Social Network Analysis

Journal:	*BMJ Open*
Manuscript ID	bmjopen-2019-028943.R3
Article Type:	Research
Date Submitted by the Author:	05-Jul-2019
Complete List of Authors:	Ruducha, Jenny; Braintree Global Health<br>Hariharan, Divya; Institute for Financial Management and Research<br>Potter, James; Harvard T.H. Chan School of Public Health, Global Health and Population<br>Ahmad, Danish; University of Canberra, Centre for Research and Action in Public Health; Public Health Foundation of India, Indian Institute of Public Health<br>Kumar, Sampath; Rajiv Gandhi Mahila Vikas Pariyojana<br>Mohanan, P.S.; Rajiv Gandhi Mahila Vikas Pariyojana<br>Irani, Laili; Population Council<br>Long, Katelyn ; Boston University School of Public Health
**Primary Subject Heading**:	Global health
Secondary Subject Heading:	Public health, Sociology, Health services research
Keywords:	Social Network Analysis, Health Services Coordination Network, Multisectoral Coordination, Demand Side Intervention, Bridging Social Capital, Self Help Groups

SCHOLARONE™
Manuscripts

Measuring Coordination between Women's Self-Help Groups and Local Health Systems in Rural India: A Social Network Analysis

Jenny Ruducha[1], Divya Hariharan[2], James Potter[3], Danish Ahmad[4], Sampath Kumar[5], P.S. Mohanan[5], Laili Irani[6] and Katelyn N.G. Long[7]

1. Braintree Global Health, Lead Partner, Cambridge, MA USA; jenny@braintreeglobalhealth.org
2. Institute for Financial Management and Research, Chennai, India; divya.hariharan@ifmr.ac.in
3. Department of Global Health and Population, Harvard T.H. Chan School of Public Health, Harvard University, Boston, MA; jpotter@g.harvard.edu
4. Centre for Research and Action, Faculty of Health, University of Canberra, Bruce, Canberra, ACT 2601, Canberra; Public Health Foundation of India and Indian Institute of Public Health, Gandhinagar, India; danish.ahmad@canberra.edu.au
5. Rajiv Gandhi Mahila Vikas Pariyojana, Raibareli, Uttar Pradesh, India; sampath97@gmail.com; psmohanan@rgmvp.org
6. Population Council, New Delhi, India; laili.ir@gmail.com
7. School of Public Health, Boston University, Boston, MA; knlong@bu.edu

Correspondence: Dr. Jenny Ruducha, 4559 West 1st Ave, Vancouver, BC, V6R1H7, Canada; jenny@braintreeglobalhealth.org; Tel: 617-480-0158

ABSTRACT WORD COUNT: 299
ARTICLE WORD COUNT: 4,058

KEYWORDS: Social Network Analysis, Health Services Coordination Network, Multisectoral Coordination, Demand Side Intervention, Bridging Social Capital, Self Help Groups

For peer review only

Measuring Coordination between Women's Self-Help Groups and Local Health Systems in Rural India: A Social Network Analysis

Abstract

Objectives To assess how the health coordination and emergency referral networks between women's Self Help Groups (SHGs) and local health systems have changed over the course of a two-year Learning Phase of the Uttar Pradesh Community Mobilization Project (UPCMP), India.

Design A pre-test, post-test program evaluation using social network survey to analyze changes in network structure and connectivity between key individuals and groups.

Setting The study was conducted in 18 villages located in 3 districts in Uttar Pradesh, India.

Intervention To improve linkages and coordination between SHGs and government health providers by building capacity in leadership, management and community mobilization skills of the SHG federation.

Participants A purposeful sampling that met inclusion criteria. 316 respondents at baseline and 280 respondents at endline including: SHG members, village and block level government health workers, and other key members of the community (traditional birth attendants, drug sellers, unqualified rural medical providers, pradhans or elected village heads, and religious leaders).

For peer review only - http://bmjopen.bmj.com/site/about/guidelines.xhtml

Main outcome measures Social network analysis measured degree centrality, density, and centralization to assess changes in health services coordination networks at the village and block levels.

Results The health services coordination and emergency referral networks increased in density and the number of connections between respondents as measured by average degree centrality have increased, along with more diversity of interaction between groups. The network expanded relationships at the village and block levels reflecting the rise of bridging social capital. The Accredited Social Health Activist (ASHA), a village health worker, occupied the central position in the network and her role expanded to sharing information and coordinating services with the SHG members.

Conclusions The creation of new partnerships between traditionally underrepresented communities and local government can serve as vehicle for building social capital that can lead to a more accountable and accessible community health delivery system.

Strengths and limitations of this study

- Original data to study and measure multisectoral coordination intervention between women's SHGs and local health systems

- A detailed examination of health coordination and emergency referral networks in rural India across 18 villages and 3 districts

For peer review only

- Contributes to literature as studies focusing on how economically marginalized women engage through SHGs in coordinating with government departments are not common

- Limitations of social network analysis affects the ability to assess causality and generalizability

- Limited duration of the linkages intervention during the Learning Phase may reduce ability to detect major changes, as a capacity building interventions take time

Introduction

Background and Study objective

Microfinance institutions comprised of Self-Help Groups (SHGs) are increasingly recognized as promising avenues for expanding health and social services to vulnerable populations.[1-3] In India, the concept of women's SHGs has evolved over the past three decades. The basic SHG structure remains defined as informal groups of 10-20 women from similar socio-economic backgrounds living in close proximity.[4] During the 1980s, the objective was to engage women in collective savings activities and provide access to credit. By the early 1990s, the official SHG and Bank Linkage Programme in India was led by the National Agricultural Bank for Rural Development (NABARD) and focused on loans for livelihood activities. To improve scalability, by early 2000s, the SHG model

For peer review only

grew into a key government programme providing financial access to the poor and addressing issues of social justice to improve the welfare of its members. [5]

The evidence for supporting women's SHGs continues to grow as research demonstrates that increasing poor women's access to working capital can result in improvements in education and health [2,6] and that SHGs tend to use their savings and credit for the family's well-being. Enhancing a woman's agency sets into motion new abilities to "exercise bargaining power as well as develop a sense of self-worth, a belief in one's ability to secure desired changes, and the right to control one's life".[7] As SHGs build social capital, they can also be instrumental in addressing deficits in government health systems.[8-9]

Building on the strengths of SHGs, a five-year Uttar Pradesh Community Mobilization Project (UPCMP) was established in India in 2012 to implement a package of interventions to improve the practice of healthy maternal and child behaviors and coordinate with the local government health system to improve maternal and newborn health. The project was based on activating an SHG model developed by Rajiv Gandhi Mahila Vikas Pariyojana (RGMVP), a non-governmental organisation, to expand SHGs into federated networks of Village Organisations and Block Organisations. Through capacity building and leadership training, poor and lower caste women were encouraged to access financial products, and advocate for government health services and entitlements. The objective of our study was to assess how health services

For peer review only - http://bmjopen.bmj.com/site/about/guidelines.xhtml

coordination and emergency referral networks between SHGs and local health systems along with other key stakeholders changed over the course of a two-year Learning Phase of the project using social network analysis.

Relevance of coordination networks

Coordination among community institutions is achieved through partnerships that improve responses to public and social issues [10] and are built around norms of reciprocity and trustworthiness.[11] The effectiveness of social networks is dependent on the density of community connections and the vibrancy of associations[12] to expand the relationships between diverse groups and to obtain the full range of knowledge, skills, and resources that the community needs to solve complex problems. Bridging social ties are most effective between people and organisations from typically under-represented communities and groups with expertise that can provide access to schemes and services (such as frontline workers, and doctors).[13]

SHG's role in coordination networks

SHG platforms have reached 57.9% of villages in India resulting in 4.8 million credit-linked groups in 2010, which demonstrates the broad potential for empowering communities to demand accountability from government functionaries.[1] Many social service models, such as the UPCMP, aim to promote coordination with government services to expand the exchange of resources and generate social capital.[14-18] When community networks such as SHGs create linkages with government programs and

For peer review only - http://bmjopen.bmj.com/site/about/guidelines.xhtml

providers, more resources are available and together, these groups can tackle issues that no one group can resolve by itself.[19]

Participatory policies and community initiatives have been well studied and critiqued.[20-22] However, studies focusing on how economically marginalized women engage through SHGs in coordinating with government departments are difficult to find in the published literature. Our study aims to contribute to this research gap by examining the efforts of SHGs in coordinating the delivery of health services with the local government health system, in order to build and sustain effective networks that enable the flow of resources to the poorest communities.

Methods

Setting

Uttar Pradesh (UP), at approximately 200 million population, [23] is one of the largest states in India constituting 16.5% of the country's total population. Uttar Pradesh Community Mobilization Project (UPCMP) began in 2012 with a Learning Phase in 100 administrative villages, called gram panchayats, located in ten blocks within eight districts with a goal to scale-up the intervention to 120 blocks over the five year project period. During the Learning Phase of the project, we selected one block from three districts that represented different parts of the state (Hardoi, Mirzapur, and Banda) (**Figure 1**). In each block, we then selected 6 out of the 10 Gram Panchayats, for a total of 18 Gram Panchayats in our analysis. The study was approved by the Institutional Review Board of Boston University Medical Campus: IRB Number H-32603. A consent

For peer review only - http://bmjopen.bmj.com/site/about/guidelines.xhtml

form (approved by Boston University Ethics Review Board) was read to participants and required a verbal approval.

Linkages intervention

The linkages strategy aimed to improve coordination between SHGs and government health and social services by expanding the leadership, management and community mobilization skills of the SHG federation at the Village Organisation and Block Organisation levels (Box 1). The interventions focused on: 1) facilitation of regular meetings of Village Organisation members with local government health workers and between the SHG Block Organisation with block level health functionaries (Box 1); 2) exchange of lists of pregnant and recently delivered women to promote early entry into pre-natal and post-natal care; and 3) identification and dissemination of information about entitlements and emergency health facilities.

Box 1: Structure and Roles of Federated Self Help Groups

At Community/Neighborhood Level, 10-20 women from particularly vulnerable and marginalised households are organised into Self-Help Groups. These SHGs meet regularly for the purpose of addressing common problems through mutual-support. In the case of RGMVP, SHGs are responsible for promoting savings among groups, ensuring credit access from banks, and driving community-based social and behaviour change interventions associated with maternal, newborn and child health. As SHGs form in a village, two members from each SHG are voted into a Village Organisation, whose members represent on average about 150-250 SHG women. Subsequently, two members from each Village Organisation across many villages are elected to the Block Organisation at the block level representing 5,000-7,000 women.

At the Village level, Village Organisation members' main focus was to establish functional relationships between SHG federation members and the three local government health worker cadres designated as "AAAs": the Accredited Social Health Activist (ASHA), a community health worker who gets paid based on her ability to mobilize pregnant and recently delivered women to seek recommended health services and promote institutional deliveries; the Auxillary Nurse Midwife (ANM), a trained midwife who organizes monthly health clinics in each village and supervises the ASHA; and the Anganwadi Worker (AWW), a local nutrition worker who operates a day care center and distributes supplementary food for eligible children and pregnant and lactating women.

At the Block level, Village Organization members are voted into the Block Organisation. Their roles were to develop relationships with the supervisors of the village level health workers, medical staff working at the primary health care centres and other block level government functionaries and elected office holders.

For peer review only - http://bmjopen.bmj.com/site/about/guidelines.xhtml

Study design

We developed a pre-test, post-test social network survey. The study objective was to assess how the health coordination and emergency referral networks between women's SHGs and local health systems changed over the course of the two-year Learning Phase of the UPCMP. The survey instruments were based on a validated survey design structure [24] and questions were developed and then pre-tested in a social and cultural setting similar to the study population to capture aspects of village and block level connections that would be relevant for assessing the program. One of the main survey questions asked every respondent whether they coordinated health services, including emergency referrals, with every other respondent type in the survey (Table1).

Study sample

The data were collected through a purposeful sampling methodology that included SHG members and those participating at the federated Village Organisation and Block Organisation levels, government and private health workers, RGMVP staff and other key stakeholders. Block level respondents were only interviewed about their relationship with respondents from two of the six Gram Panchayats to reduce the length of the interviews for block level respondents.

Certain roles are unique in a village or block, such as the ASHA, ANM, Pradhan or village leader. For those roles, the inclusion criteria were that the respondent agree to the

For peer review only - http://bmjopen.bmj.com/site/about/guidelines.xhtml

interview, be over age 18, and be the person responsible for that role at its respective geographic level. For roles that had multiple potential representatives at a given level, survey staff made a list of all potential respondents by consulting with staff at health facilities within each Block, RGMVP program staff, and local stakeholders in each village, and attempted to contact them in a random order. The first potential respondent that was successfully contacted was interviewed. The total sample was 596, with 316 respondents in the baseline and 280 in the endline for response rates of 94% and 82%, respectively.

Table 1 presents the complete list of respondents along with their role designations, the acronyms used in SNA visual plot construction, village or block location, and a brief summary of their respective roles. The respondents were grouped into four broad categories corresponding to their affiliation: SHG Structure, RGMVP Staff, Government Health & Nutrition, and "Other" key stakeholders. This approach incorporates elements of both relational and positional models for examining networks of relations with the network structure.[25]

Table 1: Study Respondents Defined

Acronym (used in SNA plots)	Respondent	Definition
**SHG Structure**		
GM	SHG member	Part of SHG group, involved in saving and borrowing, weekly SHG meetings
G1SS & G2SS	SHG Swasthya Sakhi (two per village)	SHG member who has received special training in health
VOSS	Village Organisation Swasthya Sakhi	SHG leader at village level trained in health and linkages strategy to coordinate health activities

For peer review only - http://bmjopen.bmj.com/site/about/guidelines.xhtml

Acronym (used in SNA plots)	Respondent	Definition
VOM & VOM2	Village Organisation member (two per village)	Leader from 2 village VOs trained in linkages strategy to coordinate health activities
VOB	Village Organisation office bearer	SHG member who holds a post at the federation of SHGs at the village level called Village Organisation
MS	Meeting Sakhi	SHG member who organizes weekly SHG meetings
BOB	Block Organisation office bearer	SHG and Village Organisation member who holds a post at the federation level called Block Organisation
BOR	Block Organisation representative from VO	A member of the Bock Organisation (BO) representing her village organisation
BOPR	Block Organisation Poverty Reduction Committee Member	A dedicated committee in the Block Organisation responsible for development strategies on mobilizing women from poor households into SHGs
**RGMVP Staff**		
RG	RGMVP	Full time field staff who works for the NGO (RGMVP) at block level
RGF	RGMVP Field Officer	Full time field staff who acts as a link between RGMVP and SHGs
RGV	RGMVP Volunteer (Community Volunteer)	A part-time volunteer from the community, who supports the full time field staff in implementation activities
RGT	RGMVP Trainer	RGMVP professional trainer who conducts field trainings for SHGs
ISC	Internal Social Capital	Nominated SHG member who is trained to support health implementation in villages by facilitating discussions in various SHG meetings
**Government Health & Nutrition**		
ASHA	Accredited Social Health Activist	Community health volunteer paid honorarium to promote basic preventative maternal and child services and is supervised by ANM
ANM	Auxiliary Nurse Midwife	Government trained community health worker who conducts monthly outreach clinics providing Maternal and Child care. ANM supervises ASHA and works with AWW
AWW	Anganwadi Worker	Government community worker providing food supplements to young children, adolescent girls, and lactating women along with preschool child education services in government operated village creches
ANMs/LHV	Auxiliary Nurse Midwife Supervisor/ Lady health visitor	ANMs who have been promoted to oversee six sub-centers covering a population of 30,000
ICDSs	Integrated Child Development Scheme Supervisor	Government worker appointed who supervises 25 village level creches called Anganwadi Centers (AWCs) where the AWW works
CDPO	Community Development Program Officer	Government worker in-charge of the ICDS Project at block level who oversees the ICDS supervisor at block level
BDO	Block Development Officer	Government social worker appointed under the ICDS scheme and entrusted with the overall responsibility of the ICDS at block level.
PHM	Primary Health Center Medical Officer	Government Medical Doctor & primary administrator of Primary Health Center (PHC)
PHN	Primary Health Center Nurse	Government Nurse providing nursing services in PHC
PHC	PHC staff	Approximately 13 Government staff appointed to administer primary care services at PHC
PHS	PHC Other	Extra support staff appointed at high delivery load PHCs
CHN	Community Health Center Nurse	Government Nurse appointed at Community Health Centers (CHCs) which constitute the secondary level of health care
CHM	Community Health Center Medical Officer	Government Medical officer who works at CHC to provide clinical services
CHC	CHC Staff	The total strength of staff (approx. 46) appointed by the government to administer specialist and referral care services at CHC
CMO	Chief Medical Officer-in-Charge	Government Medical Doctor who works as the Program director at the District level
CHS	CHC Other	Extra staff appointed at high patient load CHCs
DHN	District Hospital Nurse	Government Nurse working in 300 bed District Hospital for providing comprehensive secondary health care

For peer review only - http://bmjopen.bmj.com/site/about/guidelines.xhtml

Acronym (used in SNA plots)	Respondent	Definition
DHM	District Hospital Medical Officer	Government Medical officer who works at District hospital to provide clinical services
DHOB	District hospital OB/GYN	Government Medical Specialist appointed at District hospital to provide specialist Obstetrics and Gynecology care for women
RKS	Rogi Kalyan Samiti	A patient welfare committee set up at government hospital to ensure accountability and protection of patients' rights
**Other Stakeholders**		
PRI	Pradhan or Village Head	Elected head of a village-level constitutional body of local self-government called the panchayat who acts as focal point of contact between government officers and the village community
RL	Religious Leader	Leader of a religion recognized at the community (village) level
RMP	Rural Medical Practitioner (unqualified)	An unqualified medical practitioner who is not formally trained in providing health care services in villages. RMPs are culturally recognized and often first point of health care in communities
Dr	Medical Doctor (qualified)	Formally trained & accredited Medical Doctor that provides health care
PF	Private Health Facility Provider	Accredited medical doctor providing health services in a private health facility
DS	Drug Shop Owner	Owner of a private chemist shop in villages who dispenses drugs and health advice
BPRI	Block Panchayat Raj Institution	The middle tier of the system of local self-government in India operating at administrative level of block that links villages with Districts

* Technical Support Unit (TSU) project workers: TSUC – TSU Community Specialist; TSUN – TSU Nurse Mentor; TSUB – TSU Block Coordinator

For peer review only

Data collection

The baseline data were collected between November 2013 - January 2014 and endline surveys were adminstered between October - November 2015. Surveys were developed in CSPro 6.0 and adminstered using electronic tablets. During both data collection periods, the survey team spent two weeks in each of the three survey districts. Each team had a supervisor that monitored data quality and a UPCMP project representative that provided logistical support.

Analytic structure and network measures

In consideration of the models to guide network measurement and analysis at the individual and whole network level, our work fits into Mays and Scutchfield's (2010) categorization including: degree centrality, density, and centralization.[13] These

For peer review only - http://bmjopen.bmj.com/site/about/guidelines.xhtml

measures are used to assess changes in health services coordination networks within the Gram Panchayat level and between key players comprising Gram Panchayat -Block relationships. *Degree centrality* measures the number of connections or ties that each respondent maintains [26] and our analysis is based on a mutual confirmation process. In other words, if one person acknowledges a relationship and the other person does not, that relationship is dropped from the network. Overall, 31% of ties remained after the confirmation process. The high loss of unconfirmed ties signals a weak level of connectivity whereas ties confirmed by both parties have a higher probability of producing collaborative relationships.[26] In India, the caste system exerts barriers to relationship formation and contributes to a reduction in reciprocity of ties. *Density is* often used as a measure of social capital [11] and is defined as a ratio of existing relationships or ties in comparison to the potential number of linkages.[27] *Centralization* is an expression of how tightly the network structure is organized around its most central point. The general procedure is to calculate the differences between the centrality scores of the most central point and those of all other points to generate a ratio of the actual sum of differences to the maximum possible sum of differences.[27]

Data analysis

Social network analysis methods have been used to study the structural makeup of cooperation that can lead to stronger collaborative relationships.[28-29] The analysis utilized a combination of two software tools: "R" Version 3 (www.r-project.org),[30] and UCINET Version 6.[31] The plot visualization was developed by using NetDraw.[32]

For peer review only - http://bmjopen.bmj.com/site/about/guidelines.xhtml

Patient and public involvement

No patients or members of the public were involved in the development of research questions, the design of the study, or the development of outcome measures. Also, no patients were asked to advise on interpretation or writing up of results.

Results

Descriptive

The characteristics of study respondents for the two study periods are presented in **Table 2**. Most demographic indicators were balanced across baseline and endline rounds using a chi-square test, except for caste (p<0.001). For this indicator, Scheduled Caste and Scheduled Tribe accounted for a greater share of respondents in the baseline compared with the endline, while Other Backward Caste representation was greater in the endline. The median age of the respondents was approximately 40 years. About a quarter to a third of all respondents were not educated. In both rounds of the survey, more than 50% of SHG-level respondents reported having no formal education, while all government health workers reported some education and approximately one third reported post-graduate studies. Four district level respondent roles were added in the endline.

Table 2: Comparison of Respondent Background Information

Characteristic	UP CMP Baseline 2013 (Banda, Mirzapur, Hardoi) N (%)	UP CMP Endline 2015 (Banda, Mirzapur, Hardoi) N (%)

For peer review only - http://bmjopen.bmj.com/site/about/guidelines.xhtml

Median Age	41	40
**Education**		
- No Education	100 (32)	74 (26)
- Some Primary (up to 8th standard)	63 (20)	74 26)
- Some Secondary (up to 11th standard)	40 (13)	40 (14)
- Secondary Completed (12th standard)	2 (1)	3 (1)
- Post-graduate (at least some college completed)	111 (35)	89 (32)
**Caste[1*]**		
- Scheduled Caste / Scheduled Tribe	172 (55)	106 (38)
- Other Backward Castes	71 (22)	112 (40)
- General Caste	71 (22)	62 (22)
**Affiliation**		
- SHG Structure	119 (38)	98 (35)
- RGMVP Staff	23 (7)	24 (9)
- Gov Health	77 (24)	77 (28)
- Other	97 (30)	81 (29)
**Location**		
- Purwa (Hamlet)	34 (11)	33 (12)
- Gram Panchayat (Village)	220 (70)	203 (73)
- Block	62 (20)	34 (12)
- District	0	10 (4)
**TOTAL**	**316**	**280**

The classification of castes is formalized by the Government of India into these categories and we used standard definitions to create these categories. Some of these lower caste designations enable caste groups to receive specific government benefits and subsidies. Respondents were asked to self-identify into caste categories in the survey.
* Chi Square test: $p<0.001$

Respondents were also asked whether they had friends or neighbors who were SHG members, and these results are presented **in Table 3**. The percentage of SHGs in the federated structure that knew other SHG members between the baseline and endline increased significantly from 87% to 100%. The majority of RGMVP staff respondents knew SHG members in their Gram Panchayat or village. Only about one third of government health workers knew an SHG member, while almost two thirds of respondents from the “Other” category (including the Pradhan (local leader), Drug Shop Owner, Traditional Birth Attendant etc.) knew an SHG member.

For peer review only - http://bmjopen.bmj.com/site/about/guidelines.xhtml

Table 3: Respondent with Friends or Neighbors who are SHG Members by "Affiliation" (row percentages)

	UP CMP – 2013 (Banda, Mirzapur, Hardoi) N=316			UP CMP – 2015 (Banda, Mirzapur, Hardoi) N=280		
**Affiliation**	**No (%)**	**Yes (%)**	**Don't Know (%)**	**No (%)**	**Yes (%)**	**Don't Know (%)**
**SHG Structure***	16 (13)	103 (87)	0 (0)	0 (0)	98 (100)	0 (0)
**RGMVP Staff**	4 (17)	18 (78)	1 (4)	1 (4)	23 (96)	0 (0)
**Gov Health**	45 (58)	23 (30)	9 (12)	46 (60)	26 (34)	5 (6)
**Other**	33 (34)	58 (60)	6 (6)	22 (27)	53 (65)	6 (7)
**Total**	95 (31)	202 (64)	16 (5)	69 (25)	200 (71)	11 (4)

* Fischer's Exact Test: p-value <0.0001. All other rows insignificant.

Social Network Analysis

A fixed set of Gram Panchayat and block level providers and other key community members involved in health care delivery were included in the analysis. Network measures for density, centralization and average degree centrality are presented individually for all 18 villages (6 per district) as well as the average scores across each district for the baseline and endline (**Table 4**). However, since block level respondents were asked about relationships with only two Gram Panchayats, we confirmed whole Gram Panchayat-block relationships for these two Gram Panchayat-block dyads in each of the three blocks along with results for baseline and endline (**Table 5**).

Network metrics

Density of the overall village health services and referral networks in 18 Gram Panchayats (villages) were low but increased from baseline to endline in two out of three districts: Banda (7.5% to 10.1%) and Mirzapur (4.7% to 12.6%). Hardoi had a

For peer review only - http://bmjopen.bmj.com/site/about/guidelines.xhtml

higher density at baseline compared to the other two blocks and had a slight reduction at endline (13.6% to 11.4%). While the average patterns of density change in blocks during the two year intervention period increased, variability was noted beween individual villages in each of the blocks related to their baseline characteristics, and the degree of change that occurred between baseline and endline. Although Mirzapur's density was the lowest in the Gram Panchayats measured, they had the greatest growth, as noted by tripling of their scores during the two year UPCMP intervention. Hardoi had a reduction in density to half of Gram Panchayats but remained within a close range to the other two blocks at endline.

The Gram Panchayat-block level health services and referral networks take into account the relationships between two villages and block level respondents within each block **(Table 5)**. The baseline densities were lower overall than village level networks but increased after two years of project implementation, although the level of increase was less than in the village level networks. Similar to village level network patterns, Hardoi's Gram Panchayat-block level health services and emergency referral network remained at the highest level in comparison to the other two blocks in both baseline and endline, but decreased slightly in the endline (12.4 to 10.5).

The centralization measures increased two to three-fold during the same two year intervention period, with the highest level of increase in Mirzapur Gram Panchayats, which doubled on average (15.1% to 29.2%). Within the overall average increase in all

For peer review only - http://bmjopen.bmj.com/site/about/guidelines.xhtml

blocks, Gram Panchayat-level variation was noted. This included a decrease in centralization in two Banda villages and four Hardoi villages. Centralization in the Gram Panchayat-Block networks also increased in all three blocks with the largest gains in Mirzapur (8.9% to 23.9%), while Banda increased from 18.5% to 29.0% and Hardoi from 28.9% to 30.1%.

Mirzapur and Banda had the highest increase in average degree centrality but endline results suggest similar levels of provider, SHG, key health provider, and community contacts in all three blocks with 3.1 to 3.6 contacts per person. Mirzapur started at the lowest level with only 1.5 confirmed contacts compared to Banda with 2.3 and Hardoi at 4.1, with variability at the individual village level ranging from 0.8 to 6.5 across the three blocks. All Gram Panchyat-block level measures had higher levels of contacts at baseline compared to Gram Panchayat levels with Hardoi reporting a high of 7.7 contacts. Endline average degree centrality measures in Gram Panchayat-block dyads in Banda, Mirzapur and Hardoi were 5.0, 5.2 and 7.2, respectively.

Table 4: Sociometric Gram Panchayat Level Coordination and Referral Networks: Confirmed Density, Centralization and Average Degree Centrality

**District** (1 Block per District)	**Confirmed Density (%)**		**Confirmed Centralization (%)**		**Avg. Degree Centrality**	
	Baseline	Endline	Baseline	Endline	Baseline	Endline
**Banda Gram Panchayats**						
1	10.0	7.5	17.8	13.3	3.0	2.3
2	5.8	8.3	15.1	33.8	1.8	2.5
3	10.8	16.7	31.1	39.1	3.3	5.0

For peer review only - http://bmjopen.bmj.com/site/about/guidelines.xhtml

**District** (1 Block per District)	**Confirmed Density (%)**		**Confirmed Centralization (%)**		**Avg. Degree Centrality**	
4	4.2	5.8	16.9	15.1	1.3	1.8
5	7.5	13.3	20.4	28.4	2.3	4.0
6	6.7	9.2	14.2	18.7	2.0	2.8
Average	*7.5*	*10.1*	*19.3*	*24.7*	*2.3*	*3.1*
**Hardoi Gram Panchayats**						
7	11.7	4.4	23.1	8.6	3.5	1.4
8	19.2	19.1	36.4	32.8	5.8	6.1
9	21.7	15.4	26.7	23.4	6.5	4.9
10	6.7	8.1	14.2	37.9	2.0	2.6
11	11.7	6.6	16.0	19.5	3.5	2.1
12	10.8	14.7	31.1	30.9	3.3	4.7
Average	*13.6*	*11.4*	*24.6*	*25.5*	*4.1*	*3.6*
**Mirzapur Gram Panchayats**						
13	5.8	11.7	15.1	30.2	1.8	3.5
14	2.5	8.3	11.6	33.8	0.8	2.5
15	5.8	12.5	15.1	22.2	1.8	3.8
16	3.3	19.2	10.7	50.7	1.0	5.8
17	8.3	8.3	26.7	19.6	2.5	2.5
18	2.5	15.8	11.6	18.7	0.8	4.8
Average	*4.7*	*12.6*	*15.1*	*29.2*	*1.5*	*3.8*

Table 5: Sociometric Gram Panchayat (GP)-Block Level Coordination and Referral Networks: Confirmed Density, Centralization and Average Degree Centrality

**District** (1 Block per District)	**Confirmed Density (%)**		**Confirmed Centralization (%)**		**Avg. Degree Centrality**	
	Baseline	Endline	Baseline	Endline	Baseline	Endline
**Banda**						
GP 2 + Block	7.1	7.4	12.7	34.3	4.4	4.7
GP 5 + Block	5.4	8.3	24.3	23.6	3.4	5.3
Average	6.3	7.9	18.5	29.0	3.9	5.0
**Hardoi**						
GP 8 + Block	11.1	10.9	25.2	34.2	6.9	7.4
GP 9 + Block	13.7	10.1	32.5	26.0	8.5	6.9
Average	12.4	10.5	28.9	30.1	7.7	7.2
**Mirzapur**						
GP 13 + Block	3.4	8.1	9.8	23.8	2.1	5.2

For peer review only - http://bmjopen.bmj.com/site/about/guidelines.xhtml

District (1 Block per District)	Confirmed Density (%)		Confirmed Centralization (%)		Avg. Degree Centrality	
GP 14 + Block	2.0	8.0	7.9	24.0	1.3	5.1
Average	2.7	8.1	8.9	23.9	1.7	5.2

Health services and emergency referral networks

The structures of the health services coordination and emergency referral networks were visualized for all 18 Gram Panchayats (villages) and for the six Gram Panchayat-Block dyads. We have selected one example from each Gram Panchayat-Block network to illustrate the diversity of changes that occurred in structure and network connectivity. The nodes are labelled by respondent, color-coded to reflect the type of respondent group, and shaped according to the location of the respondent's usual residence or work location. The nodes are sized by betweeness centrality with larger nodes signifying the increasing importance of that respondent in linking others that may not be directly connected to each other.

The structure and the relationships between different individuals and groups changed between baseline and after two years of UPCMP implementation. At baseline, the Mirzapur Gram Panchayat-Block health services and referral network had a "kite-like" structure with only two of the four respondent groups in the network (government health and social workers and the SHG structure) having relationships with no block level connections (**Figure 2a**). At one end, a triad of the three village health and social workers (ASHA, ANM and AWW) were connected together. The AWW had a relationship with an SHG member but had no direct connections with the SHG federated

For peer review only - http://bmjopen.bmj.com/site/about/guidelines.xhtml

structure. The SHG structures themselves (at the tail end of the kite-like structure) were not integrated. At endline (**Figure 2b**), the network structure expanded into a series of connected sets of relationships with the expansion of the number of participants and the inclusion of the traditional birth attendant (TBA) and RGMVP's Block level Internal Social Capital worker (ISC). The shift from the AWW, a role more focused on nutrition and child development, to the ASHA as the main connector to the SHG structure at two critical points, directly to an SHG member as well as the SHG Village Organisation's Swasthya Sakhii (VOSS), expanded the potential for better health services coordination. The SHG structure also became better integrated with the Village Organisation level functionaries linked together with the exception of the Village Organisation Swasthya Sakhi.

At baseline, the Banda Gram Panchayat-Block level health services and referral network was organized into two small worlds, with the SHG structure and the RGMVP SHG support structure in one cluster while the government health system and a few other key village respondents were in another separate cluster (**Figure 3a**). Within the SHG small world cluster, RGMVP was at the center of a centralized system resembling a "spoke and wheel" with members of the SHG structure at the village and block levels connecting to RGMVP but not to each other.

In the second cluster, the ASHA was a leading connector of village level respondents (AWW, the rural medical practitioner (RMP), an unqualified provider and an SHG

For peer review only

member) to the broader government health system at the Block level (ANM supervisor, primary health center medical officer (PHM) and the village oriented ANM provider). By the endline, the two separate clusters formed one network (**Figure 3b**). The ASHA became the main connector between the government health providers and the SHG structure. The SHG Village Organisation members also increased coordination within their own network and RGMVP. RGMVP scaled back from their centralized role at baseline and moved to the periphery of the network while SHG enagagment increased.

In Hardoi, the structure remained similar since they started with one whole network, however an additional project, the Technical Support Unit (TSU), a supply side intervention working with the health system, was just starting during the endline collection period (**Figures 4a – 4b**). The ANM held the central position in the endline heath services and emergency referral coordination network and acted as an intermediary between the government health functionaries at the block level and the SHG federated structure. The ASHA also stood out as more direct connectivity was established with different members of the SHGs. Within the SHG platform itself, there were more channels of direct communication between different members and women in designated leadership positions.

Discussion

For peer review only

After two years of UPCMP project implementation, the health services coordination and emergency referral networks increased in their density but absolute levels remained low. This was a positive direction as higher density is considered an overall indicator of cohesion and interaction within a network and is often associated with faster rates of information diffusion within a community.[11] As information is more quickly shared, processed and appropriated, it can lead to shared decision making.[33]

However, our study results have limitations related to the constraints of social network analysis, minimal prior research on coordination networks between SHGs and government, and limited duration of testing the full effects of the linkages intervention. SNA has been mainly used for descriptive purposes and has been less frequently used for evaluating interventions [34] and therefore needs further testing to determine broader generalizability. Sources of bias may include using purposive sampling to identify the respondent roles in the villages and blocks. Additionally, there are no established criteria for evaluating networks [35] as SNA represents a relatively new multi-disciplinary methodology with limited empirical studies. While there is evidence in the literature that the constructs of density and network centrality may influence partnership functioning and coordination, their magnitudes and mechanisms of effect in public health are still largely unknown.[13] Our study provides more empirical evidence to further explore the potential of SNA to measure coordination networks.

For peer review only - http://bmjopen.bmj.com/site/about/guidelines.xhtml

Our study demonstrated an increase in connections not only between individuals but also an expansion of relationships between groups. Poor and traditionally lower-caste SHGs developed skills to cross boundaries and forge relationships with health providers. Traditionally bound by societal and cultural expectations, it has been difficult to overcome social and structural barriers, which limit interactions among heterogenous groups. A strategy based on increasing diversity in relationships while actively working to reduce redundancy can lead to improved levels of bridging social capital in a network. Such an approach that connects essential groups can lead to better ways of coordinating and collaborating.[11] Therefore, SHGs with increasing skills and confidence in engaging with health services providers are creating in-roads to better health services for themselves and their communities.

As proxies for bridging social capital, the average degree centrality increased by creating new connections within the two years of UPCMP implementation. However, simply increasing the number of ties may not necessarily result in strong relationships, as noted by the weak ties theory.[36] Although it is common to surround ourselves with strong ties that include people similar to us in beliefs, values, and access to resources, it is through weak ties that we begin to diversify our networks and create avenues for accessing more varied resources.[11] It is probable that building SHG leadership and coordination skills, which was a major UPCMP intervention, facilitated a pathway for SHGs to create weak ties with the health system. These patterns reflect opportunities for diverse stakeholders to engage in the planning and production of change.[37]

For peer review only - http://bmjopen.bmj.com/site/about/guidelines.xhtml

Our analysis showed that the ASHA occupied the central position in the network and her role expanded to coordinating services with the Village Organization Swasthya Sakhi and others in the SHG federation. The ASHA is a community health volunteer and her role evolved from an "activist" and advocate of her village to being part of the government health system and being accountable to the ANM or nurse midwife as well as the Pradhan (leader) in the Village Health, Nutrition and Sanitation Committee. As an important and recognized member of the community, the ASHA's modeling of cross-caste and class relationships can influence the governance dynamics as building collaborative networks are considered a more democratic means of developing public policy.[38-41]

Conclusions

The SHG platform and its federated structure was developed by RGMVP to scale-up and galvanize SHG development to reduce poverty, support women's empowerment, and break caste based hierarchies to encourage comprehensive rural development. The endline social network analysis reveals an expansion of pathways to coordinate health services and emergency referrals for poor, illiterate, and low caste women through the added voices of SHG members. The ability of women with multi-generational social and economic deprivations to broaden their exposure to social networks through SHG membership is a major step towards gaining self-confidence in participatory community development.

For peer review only - http://bmjopen.bmj.com/site/about/guidelines.xhtml

The increasing success of SHG federatations to forge linkages with the health system led to greater coordination for health services delivery while stimulating a more centralized structure of core village health workers. Collaborative processes that include individual empowerment, bridging social ties, and synergy can strengthen the capacity to solve problems.[42] These ideas feed into the concept of state-society syngery where a mobilized civil society and an active government can work together to build social capital and enhance each other's development efforts.[11,43]

If successful, these partnerships can serve as vehicles for transforming public health from a diverse collection of activities and organisations into an organized and accountable delivery system.[13] The challenge will be in maintaining these networks, so that they remain dynamic and offer new benefits to partners.[24] Future studies that include longitudinal data can provide deeper understanding of the mechanisms by which intersectoral partnerships and community mobilization lead to effective coordination networks to tackle health problems and their socioeconomic determinants.

Citations/References

[1] Microfinance Summit Campaign. Mapping Pathways out of Poverty: The State of the Microcredit Summit Campaign Repor, 2015.

For peer review only - http://bmjopen.bmj.com/site/about/guidelines.xhtml

Link: https://stateofthecampaign.org/read-the-full-2015-report/

[2] Leatherman S, Metcalfe M, Geissler K, Dunford C. Integrating microfinance and health strategies: examining the evidence to inform policy and practice. *Health Policy Plan* Feb 22 2011;27(2):85-101.
Link: https://doi.org/10.1093/heapol/czr014

[3] Ruducha J, Jadhav M. A review of organizational arrangements in microfinance and health programs. *J Glob Health Rep* 2018 Jul;25;2.
Link: http://www.joghr.org/Article/joghr-02-e2018024

[4] Messiana DN. Social Networking of Self-Help Groups in Tribal Villages of Srikakulam District of Andhra Pradesh – A critical Analysis (Doctoral Disseration), *Acharya NG Ranga Agricultural Unviersity,* 2012
Link: http://krishikosh.egranth.ac.in/displaybitstream?handle=1/67004

[5] Reddy CS, Manak S. Self-help groups: A keystone of microfinance in India-women empowerment and social security. *Andhra Pradesh Mahila Abhivruddhi Society (APMAS) India: Hyderabad* Oct 2005.
Link: http://www.arabic.microfinancegateway.org/sites/default/files/mfg-en-paper-self-help-groups-a-keystone-of-microfinance-in-india-women-empowerment-social-security-oct-2005.pdf

[6] Garikipati S. The impact of lending to women on household vulnerability and women's empowerment: evidence from India. *World Development* Dec 1 2008;36(12):2620-42.
Link: https://doi.org/10.1016/j.worlddev.2007.11.008

[7] Unifem. Progress of the World's Women 2000. Biennial Report, United Nations Development Fund for Women, New York, 2000.
Link: http://www.unwomen.org/en/digital-library/publications/2000/1/progress-of-the-world-s-women-2000

[8] IFAD. Enabling the Rural Poor to Overcome Poverty, 2003.
Link: https://www.ifad.org/documents/38714170/39309792/e_1.pdf/c75a166d-ecee-46af-abd3-0da951504970

For peer review only - http://bmjopen.bmj.com/site/about/guidelines.xhtml

[9] Anderson L. Self-Help Groups in Development: A Review of Evidence from South Asia and Sub-Saharan Africa. Evans School of Public Affairs, University of Washington. Prepared for the Strategy, Measurement and Evaluation Team of the Bill and Melinda Gates Foundation, 2014.
Link: https://evans.uw.edu/sites/default/files/EPAR_283_SHG%20Evidence%20Review%20Brief_12.5.14_0.pdf

[10] Nowell B. Profiling capacity for coordination and systems change: The relative contribution of stakeholder relationships in interorganizational collaboratives. *Am J Community Psychol* Dec 1 2009;44(3-4):196-212.
Link: https://doi.org/10.1007/s10464-009-9276-2

[11] Varda DM. A network perspective on state-society synergy to increase community-level social capital. *Nonprofit Volunt Sect Q* Oct 2011;40(5):896-923.
Link: https://doi.org/10.1177/0899764010378171

[12] Putnam RD. The prosperous community. *Am Prospect* Mar 21 1993;4(13):35-42.
Link: http://staskulesh.com/wp-content/uploads/2012/11/prosperouscommunity.pdf

[13] Mays GP, Scutchfield FD. Improving public health system performance through multiorganizational partnerships. *Prev Chronic Dis* Nov 2010;7(6).
Link: https://www.ncbi.nlm.nih.gov/pmc/articles/PMC2995603/

[14] Gulati R, Gargiulo M. Where do interorganizational networks come from?. *Am J Psychol Mar* 1999;104(5):1439-93.
Link: https://doi.org/10.1086/210179

[15] Isett KR, Provan KG. The evolution of dyadic interorganizational relationships in a network of publicly funded nonprofit agencies. *J Public Adm Res Theory* Jan 1 2005;15(1):149-65.
Link: https://doi.org/10.1093/jopart/mui008

[16] Mandell MP, editor. Getting results through collaboration: Networks and network structures for public policy and management ABC-CLIO, 2001.

For peer review only - http://bmjopen.bmj.com/site/about/guidelines.xhtml

[17] Monge PR, Fulk J, Kalman, ME., Flanagin AJ, Parnassa C, Rumsey S. Production of collective action in alliance-based interorganizational communication and information systems. *Organization Science* 1998;9:411-433.
Link: https://doi.org/10.1287/orsc.9.3.411

[18] Westley F, Vredenburg H. Interorganizational collaboration and the preservation of global biodiversity. *Organization Science* 1997;8:381-403.
Link: https://doi.org/10.1287/orsc.8.4.381

[19] Witte JM, Reinicke W, Benner T. Networked governance: Developing a research agenda. Paper presented at the *Annual Convention of the International Studies Association Meetings*, New Orleans, LA, 2002.

[20] Jones PS. Urban regeneration's poisoned chalice: is there an impasse in (community) participation-based policy? *Urban Stud* 2003;40(3):581-601.
Link: https://doi.org/10.1080/0042098032000053932

[21] Karim L. Demystifying micro-credit: the Grameen Bank, NGOs, and neoliberalism in Bangladesh. *Cultural Dynamics* Mar 2008;20(1):5-29.
Link: https://doi.org/10.1177/0921374007088053

[22] Kothari U, Cooke B (editor). Power, knowledge and social control in participatory development. In *Participation: The New Tyrann*y? Zed Books, London2001;139-52.

[23] Registrar General I. Census of India 2011: provisional population totals-India data sheet. Office of the Registrar General Census Commissioner, India. *Indian Census Bureau* 2011.

[24] Provan KG, Veazie MA, Staten LK, Teufel-Shone NI. The Use of Network Analysis to Strengthen Community Partnerships. *Public Adm Rev* 2005;65:603–613.
Link: https://doi.org/10.1111/j.1540-6210.2005.00487.x

[25] Burt RS. Brokerage and closure: An introduction to social capital. Oxford University Press, 2005.

[26] Freeman LC. Centrality in social networks conceptual clarification. *Soc Networks* 1978;1(3):215-39.

For peer review only - http://bmjopen.bmj.com/site/about/guidelines.xhtml

Link: https://www.bebr.ufl.edu/sites/default/files/Centrality%20in%20Social%20Networks.pdf

[27] Hanneman R, Riddle M. Introduction to Social Network Analysis. *Riverside CA: University of California,* Oct 1 2005.
Link: http://faculty.ucr.edu/~hanneman/nettext/

[28] Scott J. Social Network Analysis. Sage, 2017

[29] Wasserman S, Faust K. Social network analysis: Methods and applications. Cambridge University Press, 1994.

[30] Team RC. R: A language and environment for statistical computing.
Link: www.r-project.org

[31] Borgatti SP, Everett MG, Freeman LC. UCINET for Windows: Software for social network analysis [computer software]. Natick, MA: Analytic Technologies, 2002.

[32] Borgatti SP. NetDraw Software for Network Visualization. Analytic Technologies: Lexington, KY, 2002.

[33] Wensing M, Van Lieshout J, Koetsenruiter J, Reeves D. Information exchange networks for chronic illness care in primary care practices: an observational study. *Implement Sci* 2010;5(1):3.
Link: https://doi.org/10.1186/1748-5908-5-3

[34] DeBruin A, McAuliffe E. Social Network Analysis as a methodological approach to explore health systems: A case study exploring support among senior managers/executives in a hospital network. *Int J Environ Res Public Health* 2018;15(3):511.
Link: https://doi.org/10.3390/ijerph15030511

[35] Balkundi P, Kilduff M. The ties that lead: A social network approach to leadership. *Leadersh Q* 2006;17:419–439.
Link: https://doi.org/10.1016/j.leaqua.2006.01.001

For peer review only

[36] Granovetter MS. The strength of weak ties. *In Social Networks* Jan 1 1977 (pp. 347-367) Academic Press.
Link: https://doi.org/10.1016/B978-0-12-442450-0.50025-0

[37] Butterfoss FD, Goodman RM, Wandersman A. Community coalitions for prevention and health promotion: Factors predicting satisfaction, participation, and planning. *Health Educ Q* 1996;23(1):65-79.
Link: https://doi.org/10.1177/109019819602300105

[38] Powell WW, Koput KW, Smith-Doerr L. Interorganizational collaboration and the locus of innovation: Networks of learning in biotechnology. *Adm Sci Q* 1996;116-45.
Link: https://www.jstor.org/stable/2393988

[39] Jennings Jr ET, Ewalt JA. Interorganizational coordination, administrative consolidation, and policy performance. *Public Adm Rev* 1998;417-28.
Link: https://www.jstor.org/stable/977551

[40] Milward HB, Provan KG. Measuring network structure. *Public Adm* 1998;76(2):387-407.
Link: https://doi.org/10.1111/1467-9299.00106

[41] Provan KG, Milward HB. A preliminary theory of interorganizational network effectiveness: A comparative study of four community mental health systems. *Adm Sci Q* 1995;1-33.
Link: https://www.jstor.org/stable/2393698

[42] Lasker RD, Weiss ES. Broadening participation in community problem solving: a multidisciplinary model to support collaborative practice and research. *J Urban Health* 2003;80(1):14-47.
Link: https://doi.org/10.1093/jurban/jtg014

[43] Evans P. Government action, social capital and development: reviewing the evidence on synergy. *World Development* 1996;24(6):1119-32.
Link: https://doi.org/10.1016/0305-750X(96)00021-6

For peer review only - http://bmjopen.bmj.com/site/about/guidelines.xhtml

FOOTNOTES

Contributers: Conceptualization and Project Design, JR; Methodology, JR and JP; Network Analysis, JP; Qualitative Analysis: JP, JR, DH; Interpretation: JR, JP, DH; Interviewer Training, JP, DH, JR; Field Investigation, DH, DA, SK, PSM, JP; Draft preparation, JR; Literature Review, JR; Revising and Editing, JR, DH, LI, JP, KNGL, DA.

Acknowledgements: The authors would like to thank Dileep Mavalankar for his leadership of the Uttar Pradesh Community Mobilization Project, Deborah Maine, formerly at Boston University Center for Global Health and Development (BU CGHD) who supported the study's new methods and ME Khan, formerly of the Population Council for his knowledge and open discussions and that led to many ideas about how to study rural health systems. Many staff members of the Rajiv Gandhi Mahila Vikas Pariyojana including Gyanendra Verma and Kapil Patil and the Public Health Foundation of India (Anuraag Chaturvedi, Sanjit Sarkar and Srinivasan Soundararajan) assisted in the process of implementing this research project. We also acknowledge the administrative support and data collection oversight provided by Ariel Falconer, formerly at BU CGHD for the baseline and Steven Crimaldi of BU CGHD, for the endline. Lastly, we would like to thank the study respondents who offered their time and information to make this research possible.

Funding: This work was supported by the Bill and Melinda Gates Foundation grant number OPP 1033910.

Data Availability: Additonal unpublished data are available from the corresponding author upon request.

Competing Interests: None declared.

Participant Consent: Consent form (approved by Boston University Ethics Review Board) was read to particiapnts and required a verbal approval.

Ethics Approval: The surveys were approved by the Institutional Review Board of Boston University Medical Campus**:** IRB Number H-32603.

Patient and Public Involvement: No patients or members of the public were involved in the development of research questions, the design of the study, or the development of outcome measures. No patients were asked to advise on interpretation or writing up of results.

Provenance and Peer Review: Not commissioned

For peer review only

FIGURE CAPTIONS:

Figure 1: Map of Three Study Districts in Uttar Pradesh, India (Source: https://mapsofindia.com; permission was granted by Compare Infobase, Ltd., New Delhi, India to reproduce the map with adjusted blue shading to designate study districts)

Figure 2a: Mirzapur GP-Block Coordination Network - Baseline

Figure 2b: Mirzapur GP-Block Coordination Network - Endline

Figure 3a: Banda GP-Block Coordination Network - Baseline

Figure 3b: Banda GP-Block Coordination Network - Endline

Figure 4a: Hardoi GP-Block Coordination Network - Baseline

Figure 4b: Hardoi GP-Block Coordination Network - Endline

For peer review only

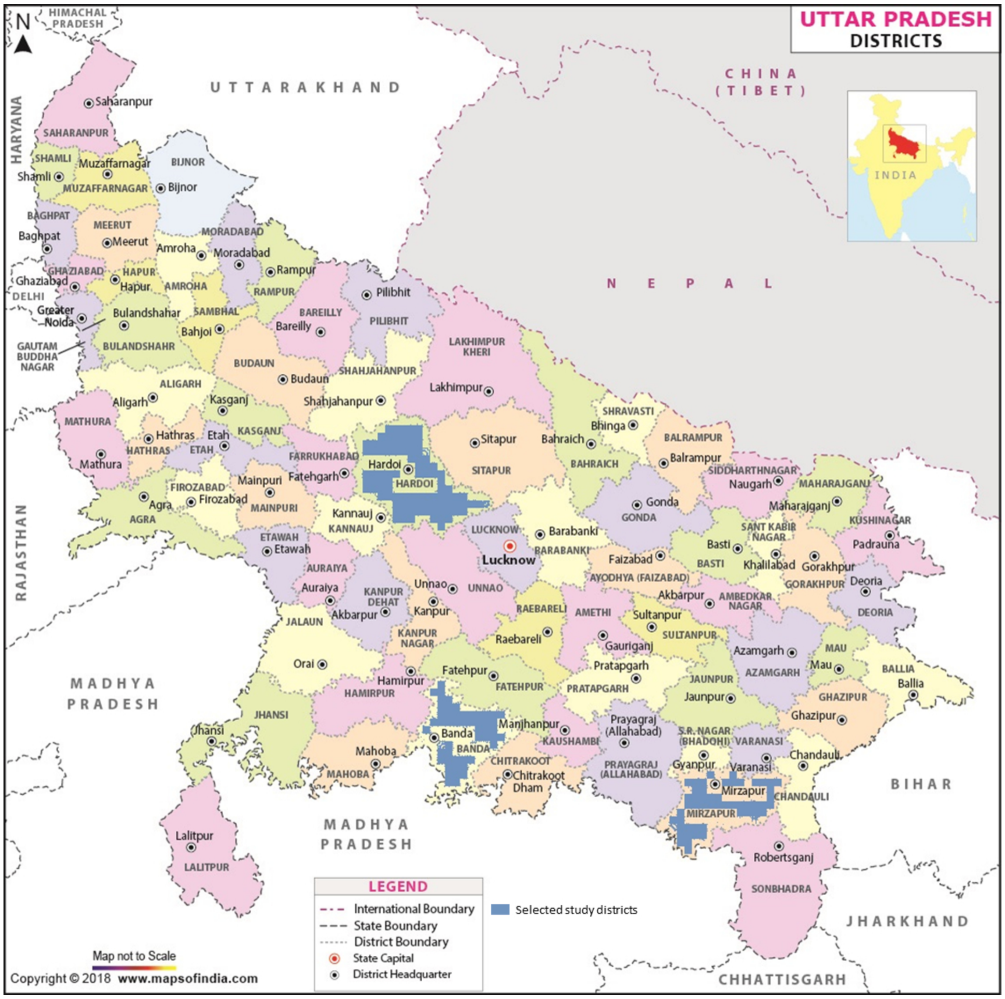

Figure 1: Map of Three Study Districts in Uttar Pradesh, India

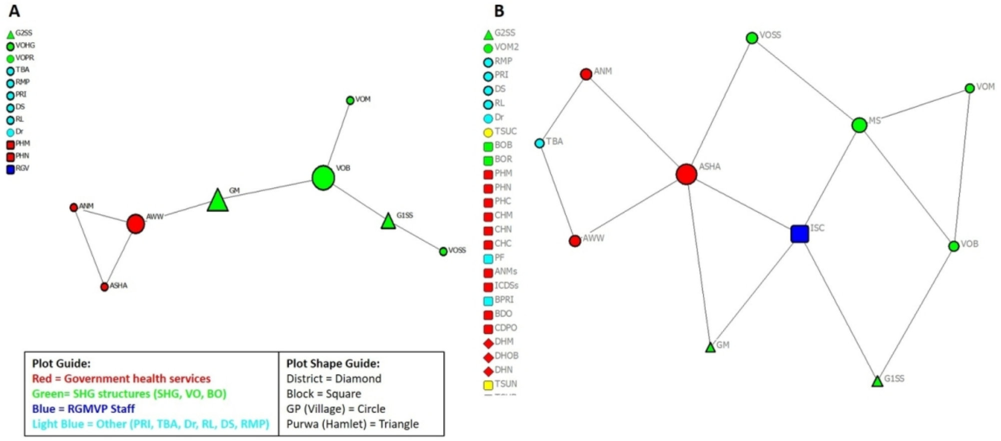

Figure 2a: Mirzapur GP-Block Coordination Network - Baseline
Figure 2b: Mirzapur GP-Block Coordination Network - Endline

62x28mm (600 x 600 DPI)

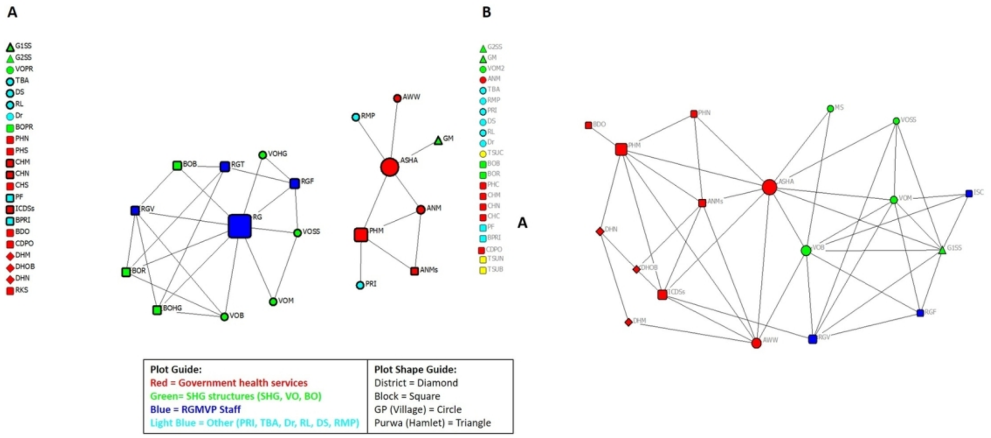

Figure 3a: Banda GP-Block Coordination Network - Baseline
Figure 3b: Banda GP-Block Coordination Network - Endline

71x32mm (600 x 600 DPI)

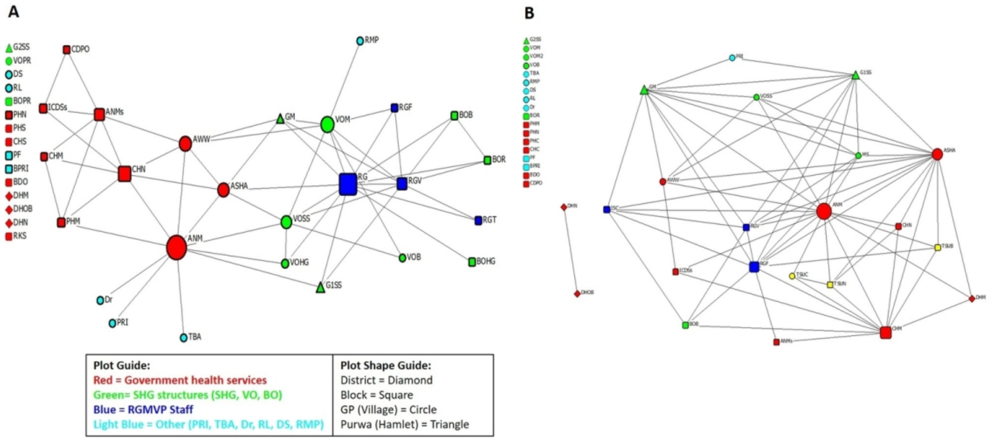

Figure 4a: Hardoi GP-Block Coordination Network - Baseline
Figure 4b: Hardoi GP-Block Coordination Network - Endline

64x29mm (600 x 600 DPI)

Reporting checklist for cross sectional study.

Based on the STROBE cross sectional guidelines.

Instructions to authors

Complete this checklist by entering the page numbers from your manuscript where readers will find each of the items listed below.

Your article may not currently address all the items on the checklist. Please modify your text to include the missing information. If you are certain that an item does not apply, please write "n/a" and provide a short explanation.

Upload your completed checklist as an extra file when you submit to a journal.

In your methods section, say that you used the STROBE cross sectional reporting guidelines, and cite them as:

von Elm E, Altman DG, Egger M, Pocock SJ, Gotzsche PC, Vandenbroucke JP. The Strengthening the Reporting of Observational Studies in Epidemiology (STROBE) Statement: guidelines for reporting observational studies.

		Reporting Item	Page Number
Title	#1a	Indicate the study's design with a commonly used term in the title or the abstract	1
Abstract	#1b	Provide in the abstract an informative and balanced summary of what was done and what was found	2-3
Background / rationale	#2	Explain the scientific background and rationale for the investigation being reported	4-6
Objectives	#3	State specific objectives, including any prespecified hypotheses	5 & 8
Study design	#4	Present key elements of study design early in the paper	8
Setting	#5	Describe the setting, locations, and relevant dates, including periods of recruitment, exposure, follow-up, and data collection	9 & 11
Eligibility criteria	#6a	Give the eligibility criteria, and the sources and methods of selection of participants.	9-11

For peer review only

	#7	Clearly define all outcomes, exposures, predictors, potential confounders, and effect modifiers. Give diagnostic criteria, if applicable	11-12
Data sources / measurement	#8	For each variable of interest give sources of data and details of methods of assessment (measurement). Describe comparability of assessment methods if there is more than one group. Give information separately for for exposed and unexposed groups if applicable.	12
Bias	#9	Describe any efforts to address potential sources of bias	25
Study size	#10	Explain how the study size was arrived at	11-12
Quantitative variables	#11	Explain how quantitative variables were handled in the analyses. If applicable, describe which groupings were chosen, and why	9-11
Statistical methods	#12a	Describe all statistical methods, including those used to control for confounding	12 & 15
	#12b	Describe any methods used to examine subgroups and interactions	14, 17-18
	#12c	Explain how missing data were addressed	N/A
	#12d	If applicable, describe analytical methods taking account of sampling strategy	N/A
	#12e	Describe any sensitivity analyses	N/A
Participants	#13a	Report numbers of individuals at each stage of study—eg numbers potentially eligible, examined for eligibility, confirmed eligible, included in the study, completing follow-up, and analysed. Give information separately for for exposed and unexposed groups if applicable.	N/A
	#13b	Give reasons for non-participation at each stage	N/A
	#13c	Consider use of a flow diagram	N/A
Descriptive data	#14a	Give characteristics of study participants (eg demographic, clinical, social) and information on exposures and potential confounders. Give information separately for exposed and unexposed groups if applicable.	12-14

For peer review only

	#14b	Indicate number of participants with missing data for each variable of interest	N/A
Outcome data	#15	Report numbers of outcome events or summary measures. Give information separately for exposed and unexposed groups if applicable.	15-17
Main results	#16a	Give unadjusted estimates and, if applicable, confounder-adjusted estimates and their precision (eg, 95% confidence interval). Make clear which confounders were adjusted for and why they were included	N/A
	#16b	Report category boundaries when continuous variables were categorized	N/A
	#16c	If relevant, consider translating estimates of relative risk into absolute risk for a meaningful time period	N/A
Other analyses	#17	Report other analyses done—e.g., analyses of subgroups and interactions, and sensitivity analyses	N/A
Key results	#18	Summarise key results with reference to study objectives	22-26
Limitations	#19	Discuss limitations of the study, taking into account sources of potential bias or imprecision. Discuss both direction and magnitude of any potential bias.	22
Interpretation	#20	Give a cautious overall interpretation considering objectives, limitations, multiplicity of analyses, results from similar studies, and other relevant evidence.	22-26
Generalisability	#21	Discuss the generalisability (external validity) of the study results	3 & 22
Funding	#22	Give the source of funding and the role of the funders for the present study and, if applicable, for the original study on which the present article is based	32

The STROBE checklist is distributed under the terms of the Creative Commons Attribution License CC-BY. This checklist can be completed online using https://www.goodreports.org/, a tool made by the EQUATOR Network in collaboration with Penelope.ai

For peer review only
